# Selenium Dibromide Click Chemistry: The Efficient Synthesis of Novel Selenabicyclo[3.3.1]nonene-2 and -nonane Derivatives

**DOI:** 10.3390/ijms242417485

**Published:** 2023-12-14

**Authors:** Maxim V. Musalov, Svetlana V. Amosova, Vladimir A. Potapov

**Affiliations:** A. E. Favorsky Irkutsk Institute of Chemistry, Siberian Division of The Russian Academy of Sciences, 1 Favorsky Str., Irkutsk 664033, Russia; amosova@irioch.irk.ru (S.V.A.); v.a.potapov@mail.ru (V.A.P.)

**Keywords:** amines, selenium dibromide, alcohols, amination, 9-selenabicyclo[3.3.1]nonene-2

## Abstract

Highly efficient and convenient methods for the preparation of 35 novel derivatives of 9-selenabicyclo[3.3.1]nonane and 9-selenabicyclo[3.3.1]nonene-2 in high yields based on the adduct of the transannular addition of SeBr_2_ to 1,5-cyclooctadiene were developed. The methods for the amination of the adduct made it possible to obtain both diamino selenabicyclo[3.3.1]nonane derivatives and their dihydrobromide salts in one step in 88–98% yields. The methods meet the criteria of click chemistry. Compounds with high glutathione peroxidase mimetic activity were found among water-soluble dihydrobromide salts. The selective reaction of 2,6-dibromo-9-selenabicyclo[3.3.1]nonane with acetonitrile to form 6-bromo-9-selenabicyclo[3.3.1]nonene-2 was discovered. The latter compound served as a promising starting material to give rise to the new class of selenabicyclo[3.3.1]nonene-2 derivatives, e.g., 6-alkoxy-9-selenabicyclo[3.3.1]nonenes were obtained in 94–99% yields.

## 1. Introduction

After the discovery of an essential biological role of selenium, the organic chemistry and biochemistry of this element began to develop rapidly. A number of organoselenium species were found in the human body, including selenoamino acids and selenium-containing enzymes, which were identified as important biocatalysts [1,2,3,4,5,6,7,8,9].

The selenium-containing enzyme glutathione peroxidase (GPx) plays a key role as a biocatalyst in the effective reduction of harmful reactive oxygen species in the human body [1,2,3,4,5,6,7,8]. Many types of biological activity of organoselenium compounds are closely related to their ability to scavenge these species including peroxides in the body [1,2,3,4,5,6,7,8,9,10,11,12,13,14,15].

Currently, the interest of scientists in the biochemistry of selenium is growing with the discovery of new organoselenium compounds, exhibiting various biological activities [16,17,18,19,20,21,22,23,24,25,26,27,28,29,30,31,32,33,34,35,36,37], including anti-HIV [16,17,18], anticancer [19,20,21,22,23,24,25,26,27,28], antibacterial [29,30,31] and glutathione peroxidase mimetic properties [32,33,34,35,36,37].

**Figure 1:**
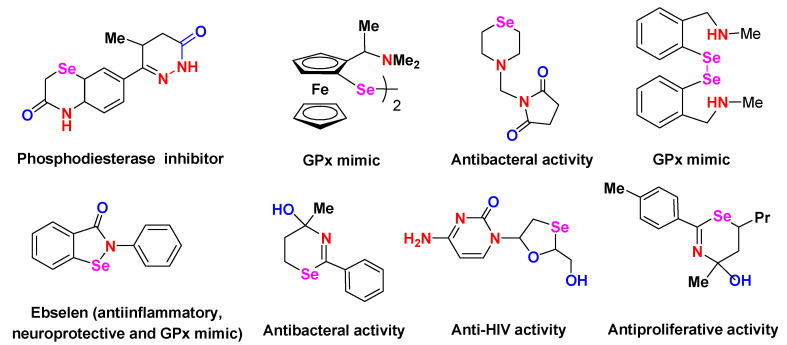
Examples of biologically active functionalized organoselenium compounds with nitrogen-containing groups: ebselen [38,39,40,41,42,43,44] and compounds with antibacterial [11], anti-HIV [16], and antiproliferative [11] activities, phosphodiesterase inhibitor [11], and GPx mimics [32].

The presence of nitrogen-containing groups in organoselenium compounds is favorable for the manifestation of biological activity (Figure 1). Many Se/N heterocycles exhibit various kinds of biological activity [16,17,18,19,20,21,22,23,24,25,26,27,28,29,30,31,32,33,34,35,36,37,38,39,40,41,42,43,44]. Some examples of functionalized organoselenium compounds with N-containing substituents, exhibiting biological activity, including GPx mimetic properties, are presented in Figure 1 [17,18,19,20,21,32].

Ebselen is the first organoselenium drug with anti-inflammatory, neuroprotective and GPx mimetic properties [38,39,40,41,42,43,44]. Ebselen exhibits antiviral activity against SARS-CoV-2, and this Se/N heterocycle has undergone clinical trials in COVID-19 patients [42,43,44]. A number of organoselenium compounds show antiviral activity [11,12,13,14,15,16,17,18,42,43,44].

Adamantane derivatives, containing amino groups, are active against various types of viruses. Special attention to these derivatives was made in a review on antiviral drugs [45]. Adamantane derivatives and their analogs continue to be intensively explored.

**Figure 2:**
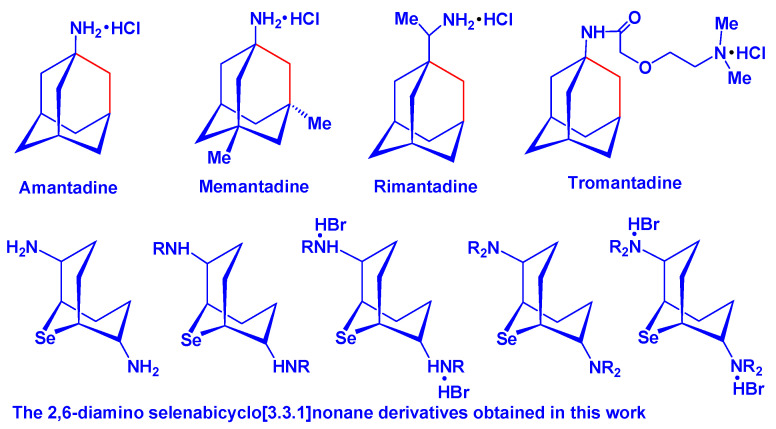
Adamantane drugs and the 2,6-diamino selenabicyclo[3.3.1]nonane derivatives obtained in this work.

A comprehensive review on the medicinal chemistry of adamantane derivatives demonstrated great potential of this class of compounds for the discovery of new drugs [46]. The high lipophilicity of the adamantane derivatives ensures the easy penetration of the drug through the blood–brain barrier [45,46]. In addition, due to this property, the amantadine derivatives can exhibit antispasmodic properties. The first adamantane derivative to be used as a medicine was amantadine (Figure 2). It was originally used as an antiviral drug effective against the influenza A2 virus. Its antiparkinsonian effect was later discovered [45,46]. Memantine is an NMDA antagonist used in the treatment of Alzheimer’s disease, but is also being tested in clinical trials as a possible therapy for a number of other conditions, including HIV-associated dementia and multiple sclerosis (Figure 2) [46]. Rimantadine is a drug active against influenza viruses and it used for the treatment and prevention of influenza (Figure 2). Tromantadine is active against Herpes viruses (Figure 2). Other active adamantane derivatives that are active ingredients in drugs include dopamantine, vildagliptin and carmantadine [45,46]. It has been established that adamantane analogues containing oxygen atoms in the tricyclic fragment of adamantane (oxaadamantanes) inhibit the replication of SARS-CoV-2 viruses [46].

One of the aims of this research is to synthesize new heterocyclic compounds, containing selenium atoms and amino groups in the form of hydrobromides, that can be structurally close to adamantane drugs (Figure 2). The presence of the selenium atom may impart a new beneficial effect to this lipophilic scaffold (e.g., GPx mimetic properties) along with possible antiviral activity.

An important direction in the field of organoselenium chemistry is the intensive development of the chemistry of selenium dichloride and dibromide—new bifunctional electrophilic reagents that make it possible to carry out new reactions and synthesize new classes of organoselenium compounds [47,48,49,50,51,52,53,54,55,56,57,58]. It is known that these reagents are disproportionate in solutions and can not be isolated in pure form [59]. In 2003, we first demonstrated the possibility of the selective synthesis of organoselenium compounds using selenium dihalides [47,48]. These reagents, generated from elemental selenium and immediately involved in situ in further reactions, can be successfully used to obtain various types of organoselenium compounds, including selenium heterocycles [47,48,49,50,51,52,53,54,55,56,57,58]. The possibility of the generation of selenium dibromide from elemental selenium and bromine was shown for the first time [47,48]. New cyclization, annulation, annulation/functionalization and selenocyclofunctionalization reactions using selenium dichloride and dibromide were recently developed [48,56,57,58].

We obtained the adduct of the transannular addition of selenium dichloride to 1,5-cyclooctadiene, which was used in collaborative studies with researchers at the Scripps Research Institute to estimate the anchimeric assistance effect of the selenium atom compared with that of the sulfur atom [56]. The rates of nucleophilic substitution reactions in 2,6-dichloro-9-thia- and 2,6-dichloro-9-selenabicyclo[3.3.1]nonanes were measured in these studies. It was found that the anchimeric assistance effect of the selenium atom is approximately two orders of magnitude greater than the effect of the anchimeric assistance of sulfur [56].

Sharpless, one of the founders of click chemistry [60], and coworkers used 2,6-dichloro-9-thiabicyclo[3.3.1]nonane in several studies for the preparation of various 9-thiabicyclo[3.3.1]nonane derivatives and considered this compound “a privileged, bivalent scaffold for the display of nucleophilic components” [61] and the click chemistry reagent, which provided the efficient and selective synthesis of the target products including compounds with biological activity [61,62,63]. Inter alia, fragmentable oligocationic compounds, which can be used for drug delivery and gene delivery into the cells, were obtained based on these compounds [63]. Selenium analogs, 2,6-dihalo-9-selenabicyclo[3.3.1]nonanes, show higher reactivity compared to 2,6-dichloro-9-thiabicyclo[3.3.1]nonane and can be also considered as the click chemistry reagents.

In connection with the coronavirus epidemic, safe vaccination and the reduction or elimination of undesirable post-vaccination reactions of the body are of particular importance. Compounds with high GPx mimetic activity can be used as metabolic correctors on the vaccination process, which reduce or eliminate the pathological reactions of the body including oxidative stress during the vaccination and post-vaccination period.

We recently synthesized 2,6-dipyridinium-9-selenabicyclo[3.3.1]nonane dibromide, which was found to be a prospective drug for metabolic correction in the vaccination and post-vaccination period [64]. This is the result of the joint research with the Irkutsk Anti-Plague Research Institute of Siberia and the Far East. The introduction of this compound into the body of experimental animals significantly reduces the development of pathological reactions [64]. It is worth noting that 2,6-dipyridinium-9-selenabicyclo[3.3.1]nonane dibromide and other selenabicyclo[3.3.1]nonane derivatives do not exhibit toxicity [64]. Thus, it is relevant to search for new organoselenium compounds with high GPx mimetic activity, which can also be used as metabolic correctors in the vaccination process, in the series of selenabicyclo[3.3.1]nonane derivatives.

## 2. Results and Discussion

The aim of this research is to develop efficient syntheses of novel functionalized (especially diamino) derivatives of 9-selenabicyclo[3.3.1]nonane, which are promising candidates for the manifestation of GPx mimetic activity. The use of click chemistry principles is an important feature of this work. Prospective metabolic correctors of the vaccination process and possible antiviral agents (Figure 2) can be found among 9-selenabicyclo[3.3.1]nonane derivatives with high GPx activity. Like the adamantane framework, the structure of 9-selenabicyclo[3.3.1]nonane has high lipophilic properties, and diamino derivatives of 9-selenabicyclo[3.3.1]nonane can be considered structurally close to adamantane antiviral drugs (Figure 2) [45,46]. Additionally, high GPx activity can import additional beneficial effects to these products.

**Scheme 1:**
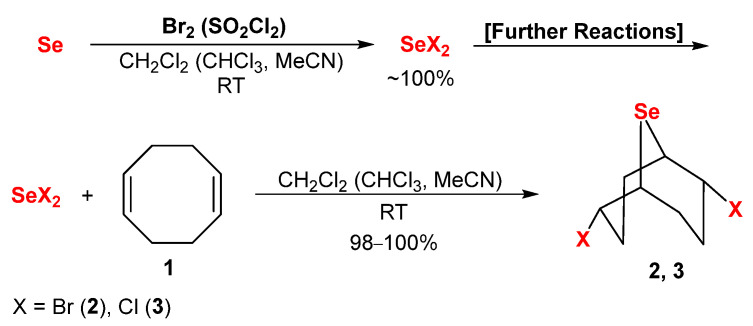
Synthesis of 2,6-dihalo-9-selenabicyclo[3.3.1]nonanes **2** and **3**.

Earlier, we developed the transannular addition of selenium dihalides to cyclooctadiene **1**, which proceeded with high selectivity and afforded 2,6-dibromo- and 2,6-dichloro-9-selenabicyclo[3.3.1]nonanes **2** and **3** in near-quantitative yields (Scheme 1) [56,57].

To study the amination reaction of compound **2**, various amines were chosen: diethylamine, diallylamine, morpholine, piperidine, pyrrolidine, allylamine, isopropylamine, diisopropylamine, butylamine, isobutylamine, tert-butylamine, benzylamine, phenylamine and 2-vinyloxyethylamine. We began our study of the amination with the reaction of compound **2** with secondary amines, which usually gives monoalkylated products.

**Scheme 2:**
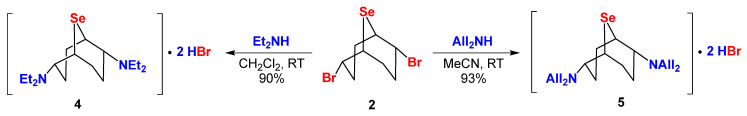
The synthesis of compounds **4** and **5**.

The reaction of diethylamine with compound **2** proceeded smoothly at room temperature in methylene chloride to give product **4** in 90% yield in the form of dihydrobromide (Scheme 2).

The reaction of unsaturated diallylamine with compound **2** in methylene chloride was sluggish, but when the reaction was carried out in acetonitrile, product **5** was obtained in 93% yield (Scheme 2). Thus, diallylamine is less nucleophilic than diethylamine and reacts efficiently in acetonitrile, which is a more polar solvent compared to methylene chloride. Products **4** and **5** were formed as precipitates, which easily separated from the reaction mixture by filtration.

**Scheme 3:**
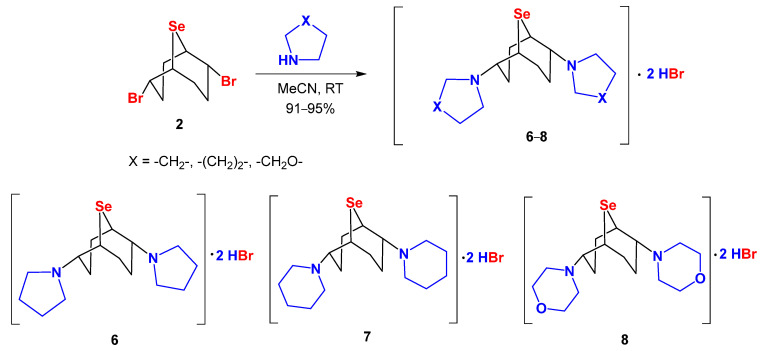
The synthesis of the products **6**–**8** from cyclic amines and compound **2**.

The morpholine, piperidine and pyrrolidine cycles are present in many drugs and can be considered the pharmacophore groups. Morpholine, piperidine and pyrrolidine were successfully involved in the nucleophilic substitution reaction with compound **2** to form corresponding dihydrobromide products **6**–**8** in 91–95% yields (Scheme 3).

**Scheme 4:**
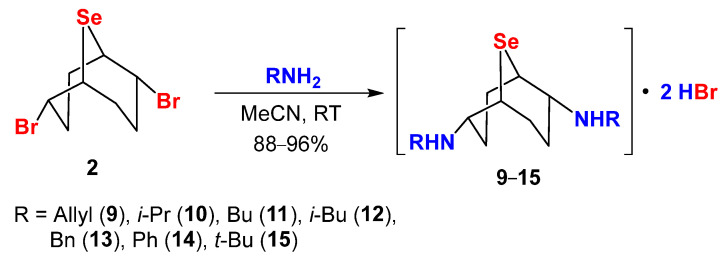
The synthesis of compounds **9**–**15** from primary amines and compound **2**.

Primary amines usually react with two equivalents of alkyl halide and it is difficult to carry out the monoalkylation. However, we succeeded in the development of the alkylation of compound **2** with two equivalents of primary amines with the formation of products with secondary amine groups in the form of dihydrobromide. In this case, it was necessary to use more acetonitrile (compared to the reactions in Scheme 2 and Scheme 3) to prevent “crosslinking”, i.e., the reaction of one molecule of primary amine with two molecules of compound **2**. Various primary amines such as allylamine, isopropylamine, butylamine, isobutylamine, tert-butylamine, benzylamine and phenylamine were involved in the reaction with compound **2,** affording corresponding diamino dihydrobromide derivatives **9**–**15** in high yields (Scheme 4).

The main part of the primary amines gave the products **9**–**14** in 91–96% yields. In the case of tert-butylamine, the yield of the product **15** was slightly lower (88%).

The products in the form of dihydrobromide salts (Scheme 2, Scheme 3 and Scheme 4) precipitated from the reaction mixture and can be easy isolated by filtration. It should be emphasized that these products did not require additional purification. The compounds obtained were generally white, beige, yellowish or slightly orange-tinted powders.

**Scheme 5:**
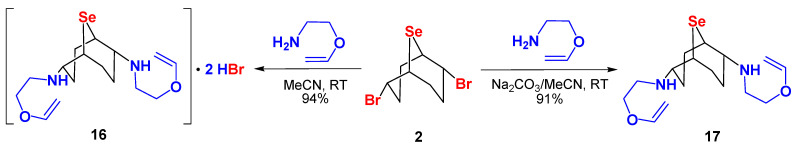
The synthesis of compounds **16** and **17**.

Compounds, containing vinyloxy groups, are valuable intermediates for organic synthesis, capable of electrophilic addition and polymerization. The amine functionalized with the vinyloxy group 2-vinyloxyethylamine reacted with compound **2** to form the product **16** in 94% yield (Scheme 5).

2-Vinyloxyethylamine was obtained by the vinylation of 2-hydroxyethylamine with acetylene in the presence of an alkali.

A very convenient and effective method for obtaining corresponding diamino derivatives as free bases was developed. Performing the reaction in acetonitrile in the presence of sodium or potassium carbonate ensures the selective formation of the corresponding diamino products due to the selective neutralization of hydrobromide with these basic reagents. Sodium or potassium carbonate was added at the beginning of the process after the amine was added to the reaction mixture. It should be noted that the possible formation of unsaturated products by the dehydrobromination reaction with the carbonates was not observed.

The reaction of 2-vinyloxyethylamine with compound **2** in acetonitrile at room temperature in the presence of sodium carbonate gave 2,6-bis(2-vinyloxyethylamino)-9-selenabicyclo[3.3.1]nonane **17** as a bifunctional free base in 91% yield (Scheme 5).

We suppose that the products **16** and **17**, containing two vinyloxy groups, can be useful monomers in oligomerization or polymerization reactions to produce new materials, containing a 9-selenabicyclo[3.3.1]nonane scaffold and ammonium or amino groups. These very interesting products can be also involved in further functionalization reactions of vinyloxy or amino groups.

It was found that, to obtain the corresponding diamine derivatives with high yields, it is necessary to use sodium or potassium carbonate in a 1.5–2-fold excess compared to stoichiometric quantities.

**Scheme 6:**
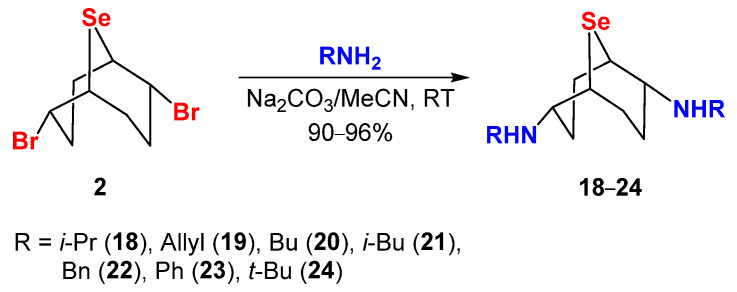
The synthesis of the products **18**–**24** from primary amines and compound **2**.

This method has proved to be very effective in the case of both primary and secondary amines. The reaction of isopropylamine, allylamine, butylamine, isobutylamine, tert-butylamine, benzylamine and phenylamine with compound **2** was carried out in the Na_2_CO_3_/acetonitrile system at room temperature, affording corresponding 2,6-diamino derivatives **18**–**24** in 90–96% yields (Scheme 6).

**Scheme 7:**
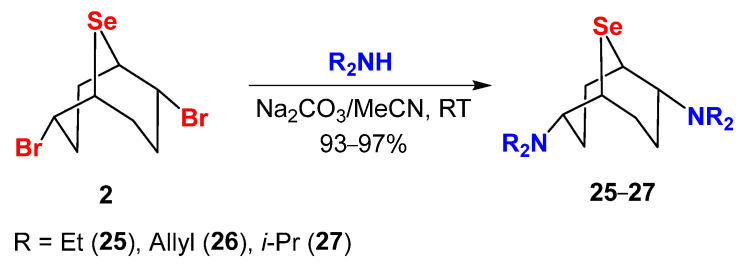
The synthesis of compounds **25**–**27**.

Secondary amines such as diethylamine, diisopropylamine and diallylamine were efficiently involved in the reaction with compound **2** in acetonitrile at room temperature in the presence of sodium carbonate to form corresponding 2,6-diamino derivatives **25**–**27** in 93–97% yields (Scheme 7).

**Scheme 8:**
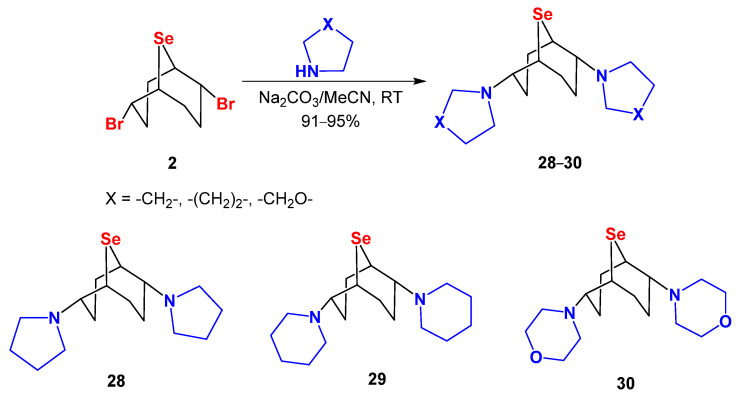
The synthesis of compounds **28**–**30**.

When the reaction of morpholine, piperidine and pyrrolidine with compound **2** was carried out in acetonitrile at room temperature in the presence of sodium or potassium carbonate, corresponding 2,6-diamino derivatives **28**–**30** were obtained in 91–95% yields (Scheme 8).

**Scheme 9:**
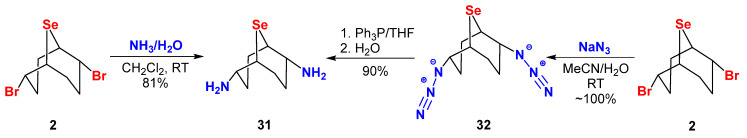
Two methods for the preparation of compound **31**.

We succeeded in the preparation of product **31**, containing two unsubstituted amino groups, by reacting compound **2** with ammonia in a two-phase system: methylene chloride-10% aqueous ammonia solution containing potassium carbonate. The reaction mixture was allowed to stir for 40 h at room temperature. Despite these very simple conditions, the target product **31** was obtained in 81% yield (Scheme 9).

A higher yield of product **31** was achieved starting from diazido derivative **32**, which was obtained from compound **2** and sodium azide in aqueous acetonitrile. The reduction of diazido derivative **32** with triphenylphosphine in THF followed by the hydrolysis afforded the target product **31** in 90% yield (Scheme 9).

It should be emphasized that the developed methods demonstrate the valuable features of click chemistry: high yields of target products, simple reaction conditions (room temperature), accessible starting materials, broad-scope reactions (ammonia, both primary and secondary amines with various substituents: Et, Bu, i-Bu, i-Pr, t-Bu, Bn, Ph, morpholine, piperidine, pyrrolidine and 2-vinyloxyethylamine) and convenient isolation procedures (no additional purification of the products required).

**Scheme 10:**
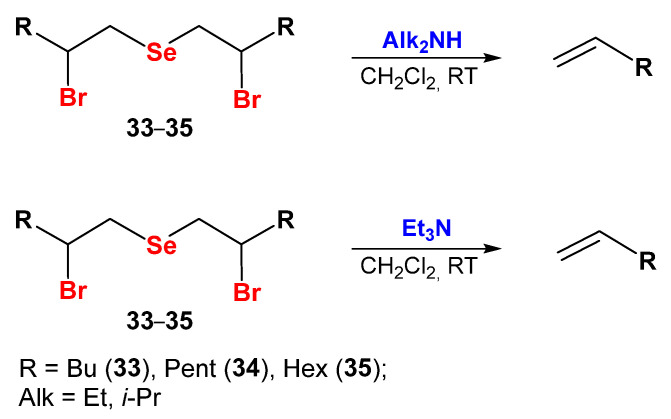
The reactions of the SeBr_2_-alkene adducts with amines.

It is worth noting that the adducts of selenium dibromide with simple alkenes **33**–**35** do not undergo nucleophilic substitution with amines. We found that the reactions of diethyl, diisopropyl and triethylamine with the SeBr_2_-alkene adducts **33**–**35** led to the formation of parent alkenes (Scheme 10). We assume that under the action of the amines, a process occurs that is the reverse reaction of the addition of selenium dibromide to alkenes. However, the use of compound **2** made it possible to carry out the nucleophilic substitution reactions with amines.

The reaction of dibromo compound **2** with triethylamine, which is more basic than other amines, proceeded non-selectively and was accompanied by dehydrobromination with the formation of a mixture of products. We found that the dehydrobromination of product **2** can be carried out more selectively by heating this compound in acetonitrile, which plays the role of a soft dehydrobromination reagent in this case. Heating compound **2** in acetonitrile made it possible to eliminate selectively one hydrobromide molecule from one molecule of compound **2** with the formation of the CH=CH moiety and to obtain a very interesting product, containing one double bond and one bromine atom: 6-bromo-9-selenabicyclo[3.3.1]nonene-2 (**36**).

**Scheme 11:**
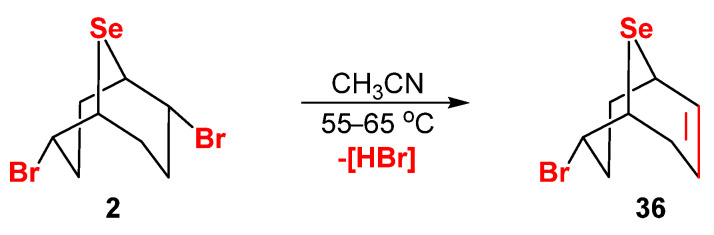
The synthesis of 6-bromo-9-selenabicyclo[3.3.1]nonene-2 (**36**).

Refluxing compound **2** in acetonitrile (~82 °C) for 3 h gave nonene **36** in about 50% yield, but the selectivity of this reaction was low and it was difficult to purify the target product. It was found that it is preferable to carry out the heating reaction at a lower temperature (55–65 °C) with an increase in the reaction time to 12 h. Under these conditions, the target product **36** was selectively obtained in 90% yield and could be easily purified (Scheme 11).

This remarkable reaction makes it possible to develop the convenient synthesis of 6-bromo-9-selenabicyclo[3.3.1]nonene-2 (**36**), which serves as a promising starting material in further transformations and the preparation of a number of selenabicyclo[3.3.1]nonene derivatives.

**Scheme 12:**
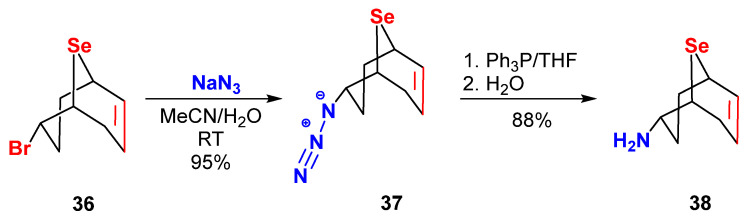
The synthesis of 6-amino-9-selenabicyclo[3.3.1]nonene-2 (**38**).

The reactions of monobromide **36** with alkylamines and ammonia, under the same condition as in the previous reactions with compound **2**, gave mixtures of products. However, we succeeded in the preparation of 6-azido-9-selenabicyclo[3.3.1]nonene-2 **37** and corresponding amino derivative **38**. Monobromide **36** was converted to azide **37** via the reaction with sodium azide in aqueous acetonitrile and then the obtained azide was reduced with triphenylphosphine in THF followed by a hydrolysis (Scheme 12). As a result, 6-amino-9-selenabicyclo[3.3.1]nonene-2 (**38**) was obtained in 88% yield.

**Scheme 13:**
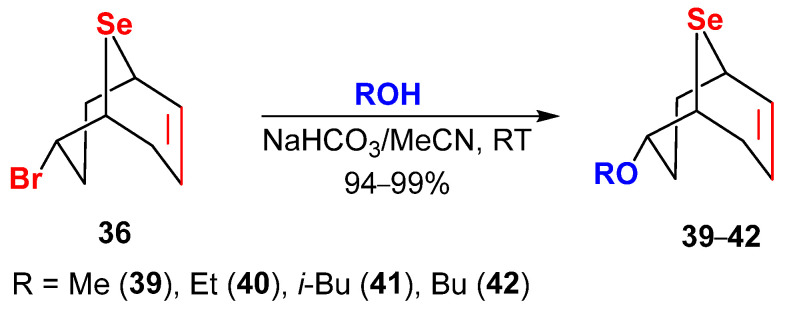
The synthesis of 6-alkoxy-9-selenabicyclo[3.3.1]nonenes-2 **39**–**42**.

A very convenient and selective synthesis of alkoxy derivatives of selenabicyclo[3.3.1]nonene **39**–**42** in 94–99% yield was developed by nucleophilic substitutions of bromine in compound **36** by alcohols. We found that the alkoxylation reaction proceeded efficiently in acetonitrile in the presence of weakly basic sodium bicarbonate at room temperature (Scheme 13).

Filtration followed by the solvent removal resulted in the pure target products **39**–**42**, which did not require additional purification. This reaction also meets the criteria of click chemistry.

The resulting 9-selenabicyclo[3.3.1]nonene-2 derivatives represent a new, very promising class of organoselenium heterocyclic compounds. The presence of the double bond and very active bromine atom opens new possibilities for functionalization. We plan to develop the chemistry of this new class of compounds using bromo derivative **36** as a starting material, as well as other approaches.

The high reactivity of compounds **2** and **36** is determined by the strong anchimeric assistance effect of the selenium atom [56]. It is assumed that the nucleophilic substitution of the bromine atom in compounds **2** and **36** proceeds via the formation of corresponding seleniranium intermediates [56]. We suppose that the dehydrobromination of compound **2** (Scheme 11) occurs as an anti-process, which is facilitated by acetonitrile as a polar solvent. Acetonitrile also exhibits the properties of a weak base in this reaction. The selenium atom is in an anti-position relative to the bromine atom and the anti-elimination of hydrobromide in the Br-CH-CH-Se group does not occur. However, there is a hydrogen atom in an anti-position to the bromine atom in the Br-CH-CH_2_- fragment and the elimination proceeds selectively in this fragment.

Acetonitrile was found to be a solvent of choice for these nucleophilic reactions (Scheme 2, Scheme 3, Scheme 4, Scheme 5, Scheme 6, Scheme 7, Scheme 8, Scheme 11, Scheme 12 and Scheme 13). Like DMSO and DMF, acetonitrile is an aprotic bipolar solvent, which considerably facilitated nucleophilic substitutions. However, unlike DMSO and DMF, acetonitrile has a lower boiling point (~82 °C) and can be easily removed from the reaction mixture. The products can be purified from acetonitrile in a vacuum, but doing the same using DMSO or DMF as a solvent is very difficult.

**Figure 3:**
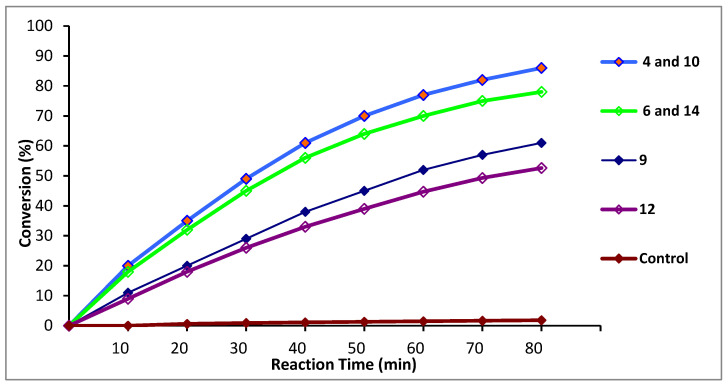
The evaluation of the GPx mimetic activity of compounds **4**, **6**, **9**, **10**, **12** and **14**.

The selenium atom is sterically accessible in the rigid configuration of the 9-selenabicyclo[3.3.1]nonane framework and its derivatives are very promising compounds for the manifestation of GPx mimetic activity. The obtained water-soluble dihydrobromide products were investigated as GPx mimics. The oxidation of dithiothreitol with *tert*-butyl hydroperoxide in deuterated water was used as the model reaction [65,66,67,68,69,70]. The synthesized products played the role of catalysts (10% mol). The progress of the redox reaction was monitored by ^1^H NMR spectroscopy at room temperature after mixing the reagents and a tested product (*tert*-butyl hydroperoxide, dithiothreitol, 0.025 mmol; a tested product, 0.0025 mmol; D_2_O, 0.5 mL). The results obtained for most active products are presented in Figure 3.

The catalytic activities of the dihydrobromide products were estimated by the measurement of half-lives (T_1/2_), representing the time required to oxidize half of the dithiotreitol to its disulfide (this is a generally accepted methodology [65,66,67,68,69,70]). It was found that compounds **4** and **10**, containing diethylamino and isopropylamino groups, showed the greatest activity of approximately the same value among the tested compounds (Figure 3). The activity of the products **6** and **14** with pyrrolidine and phenylamino substituents were also very high and about the same value. The half-lives (T_1/2_) of these four products were in the range of 30–35 min. The comparison of the activity of these products, **4**, **6**, **10** and **14,** with known data [65,66,67,68,69,70] allows us to consider them compounds with high activity. Less active were compounds **9** and **12**, containing allylamino (T_1/2_ = 61 min) and isobutylamino (T_1/2_ = 86 min) groups. The other products showed T_1/2_ > 100 min.

The amino groups can exhibit electronic and steric effects, influencing the main catalytic center—the selenium atom in compounds **4**–**16**. We assume that the obtained results show the importance of the steric factor for this family of compounds. Diethylamino and isopropylamino substituents show low steric hindrance at the selenium atom, which can participate in the catalytic cycle. This is also true for the pyrrolidine and phenylamino substituents, which exhibit lower steric effects compared to that of the piperidine and morpholine groups. It is worth noting that the phenylamino group has a flat configuration.

We suppose that the catalytic cycle [65,66,67,68,69,70] includes the formation of intermediate selenoxides, which are formed by the oxidation of the products with *tert*-butyl hydroperoxide. The selenoxides are very reactive intermediates, which oxidase dithiotreitol to its cyclic disulfide with the formation of water and the regeneration of the catalysts [67]. This process is energetically very favorable.

The structural assignments of the synthesized compounds were made using ^1^H, ^13^C and ^77^Se NMR spectroscopy including two-dimensional experiments and were confirmed by elemental analysis. The structures of selenabicyclo[3.3.1]nonene-2 derivatives were proved by NMR spectroscopy including COSY, HMBC and HSQC experiments.

When heated in a vacuum (1 mm Hg), the obtained compounds decompose at temperatures above 100–110 °C and it is hardly possible to measure their boiling point.

## 3. Materials and Methods

### 3.1. General Information

The ^1^H (400.1 MHz), ^13^C (100.6 MHz) and ^77^Se (76 MHz) NMR spectra were recorded on a Bruker DPX-400 spectrometer (Bruker BioSpin GmbH, Rheinstetten, Germany) in CDCl_3_ (referred to the residual solvent peaks of CDCl_3_, δ = 7.27 and 77.16 ppm for ^1^H and ^13^C NMR, respectively) peaks, δ = 7.27 and 77.16 ppm for ^1^H and ^13^C NMR, respectively), DMSO-*d*_6_ (referred to the residual solvent peaks of DMSO-*d*_6_, δ = 2.50 and 39.50 ppm for ^1^H and ^13^C NMR, respectively), and D_2_O (referred to the external standard, hexamethyldisiloxane, δ = 0.055 and 1.97 ppm for ^1^H and ^13^C NMR, respectively). The ^77^Se NMR spectra were referred to dimethyl selenide as standard. The NMR spectra can be found in Supplementary Materials. IR spectra were recorded on a Vertex 70 spectrometer (Bruker BioSpin GmbH, Rheinstetten, Germany). Elemental analysis was performed on a Thermo Scientific Flash 2000 Elemental Analyzer (Thermo Fisher Scientific Inc., Milan, Italy). Melting points were determined on a Kofler Hot-Stage Microscope PolyTherm A apparatus (Wagner & Munz GmbH, München, Germany). The distilled organic solvents and degassed water were used in syntheses.

### 3.2. The Synthesis of Dihydrobromide Salts ***4**–**8*** from Secondary Amines

*2,6-Bis(diethylamino)-9-selenabicyclo[3.3.1]nonane dihydrobromide* (**4**). A solution of diethylamine (0.15 g, 2.05 mmol) in methylene chloride (1 mL) was added dropwise to a solution of compound **2** (0.3 g, 0.86 mmol) in methylene chloride (4 mL) with stirring at room temperature. The reaction mixture was stirred overnight (16 h) at room temperature. Cold hexane (5 mL) was added to the mixture. The formed precipitate was filtered off, washed with cold hexane and dried in a vacuum giving the product (382 mg, 90% yield) as a white powder, mp 184–185 °C.

^1^H NMR (400 MHz, D_2_O, ppm): δ 0.69 (t, 12H, CH_3_), 1.44–1.60 (m, 2H, CH_2_), 1.73–1.81 (m, 2H, CH_2_), 1.92–2.02 (m, 2H, CH_2_), 2.03–2.12 (m, 2H, CH_2_), 2.59–2.71 (m, 4H, CH_2_N), 2.76–2.87 (m, 4H, CH_2_N), 2.88–2.93 (m, 2H, CHSe), 3.56–3.62 (m, 2H, CHN).

^13^C NMR (100 MHz, D_2_O, ppm): δ 8.0 (CH_3_), 8.3 (CH_3_), 23.2 (CH_2_), 24.0 (CHSe), 28.6 (CH_2_), 43.5 (CH_2_N), 44.4 (CH_2_N), 64.1 (CHN).

IR (KBr): 3728, 2922, 2765, 2629, 2490, 1632, 1567, 1466, 1418, 1373, 1304, 1177, 1094, 1038, 925, 842, 791 cm^−1^.

Anal. calcd for C_16_H_34_N_2_Br_2_Se (493.22): C 38.96, H 6.95, N 5.68, Br 32.40, Se 16.01%. Found: C 39.12, H 7.08, N 5.52, Br 32.76, Se 15.87%.

*2,6-Bis(diallylamino)-9-selenabicyclo[3.3.1]nonane dihydrobromide* (**5**). A solution of diallylamine (0.2 g, 2.06 mmol) in acetonitrile (1 mL) was added dropwise to a mixture of compound **2** (0.3 g, 0.86 mmol) and acetonitrile (4 mL) with stirring at room temperature. The reaction mixture was stirred overnight (16 h) at room temperature. Cold hexane (5 mL) was added to the mixture. The formed precipitate was filtered off, washed with cold acetonitrile and dried in a vacuum, giving the product (435 mg, 93% yield) mp 161–162 °C as a white powder.

^1^H NMR (400 MHz, D_2_O, ppm): δ 1.51–1.65 (m, 2H, CH_2_), 1.78–1.87 (m, 2H, CH_2_), 1.92–2.04 (m, 2H, CH_2_), 2.09–2.16 (m, 2H, CH_2_), 2.92–2.95 (m, 2H, CHSe), 3.20–3.46 (m, 8H, CH_2_N), 3.60–3.66 (m, 2H, CHN), 5.00–5.06 (m, 8H, CH=CH_2_), 5.29–5.40 (m, 4H, CH=CH_2_).

^13^C NMR (100 MHz, D_2_O, ppm): δ 22.6 (CHSe), 23.3 (CH_2_), 27.9 (CH_2_), 50.9 (CH_2_N), 51.3 (CH_2_N), 63.9 (CHN), 124.3 (CH=CH_2_), 124.8 (CH=CH_2_), 125.9 (CH=CH_2_).

IR (KBr): 3728, 3437, 2947, 2798, 2735, 2423, 2219, 1884, 1645, 1570, 1439, 1168, 1095, 991, 940, 875, 612, 523, 417 cm^−1^.

Anal. calcd for C_20_H_34_N_2_Br_2_Se (541.26): C 44.38, H 6.33, N 5.18, Br 29.53, Se 14.59%. Found: C 44.65, H 6.19, N 4.98, Br 29.80, Se 14.81%.

*2,6-Bis(1-pyrrolidinyl)-9-selenabicyclo[3.3.1]nonane dihydrobromide* (**6**) was obtained under the same conditions as compound **5** in 95% yield as a beige powder, mp 218–219 °C.

^1^H NMR (400 MHz, D_2_O, ppm): δ 1.36–1.54 (m, 10H, CH_2_CH_2_N, CH_2_), 1.73–1.80 (m, 2H, CH_2_), 1.86–1.96 (m, 2H, CH_2_), 1.98–2.05 (m, 2H, CH_2_), 2.71–2.85 (m, 10H, CH_2_N, CHSe), 3.34–3.40 (m, 2H, CHN).

^13^C NMR (100 MHz, D_2_O, ppm): δ 22.2 (CH_2_CH_2_N), 25.0 (CH_2_), 25.9 (CHSe), 27.5 (CH_2_), 52.3 (CH_2_N), 67.4 (CHN).

IR (KBr): 3728, 2926, 2880, 2669, 2584, 2479, 1630, 1405, 1169, 1016, 972, 895, 671, 574 cm^−1^.

Anal. calcd for C_16_H_30_N_2_Br_2_Se (489.19): C 39.28, H 6.18, N 5.73, Br 32.67, Se 16.14%. Found: C 39.56, H 5.99, N 5.57, Br 32.44, Se 16.38%.

*2,6-Bis(1-piperidinyl)-9-selenabicyclo[3.3.1]nonane dihydrobromide* (**7**) was obtained under the same conditions as compound **5** in 94% yield as a beige powder, mp 129–130 °C.

^1^H NMR (400 MHz, D_2_O, ppm): δ 0.88–0.98 (m, 2H, CH_2_CH_2_CH_2_N), 1.15–1.26 (m, 6H, CH_2_CH_2_CH_2_N), 1.33–1.45 (m, 4H, CH_2_CH_2_N), 1.51–1.63 (m, 2H, CH_2_), 1.83–2.02 (m, 4H, CH_2_), 2.06–2.14 (m, 2H, CH_2_), 2.39–2.51 (m, 4H, CH_2_N), 2.97–3.04 (m, 2H, CHSe), 3.05–3.19 (m, 4H, CH_2_N), 3.50–3.58 (m, 2H, CHN).

^13^C NMR (100 MHz, D_2_O, ppm): δ 21.2 (CH_2_CH_2_CH_2_N), 22.7 (CH_2_CH_2_N), 23.1 (CHSe), 23.8 (CH_2_), 28.5 (CH_2_), 50.2 (CH_2_N), 51.6 (CH_2_N), 67.7 (CHN).

IR (KBr): 3436, 2941, 2639, 2536, 1632, 1454, 1366, 1292, 1199, 1146, 1101, 1013, 930, 717, 557, 451 cm^−1^.

Anal. calcd for C_18_H_34_N_2_Br_2_Se (517.24): C 41.80, H 6.63, N 5.42, Br 30.90, Se 15.27%. Found: C 42.07, H 6.46, N 5.42, Br 31.14, Se 14.98%.

*2,6-Dimorpholinyl-9-selenabicyclo[3.3.1]nonane dihydrobromide* (**8**) was obtained under the same conditions as compound **5** in 91% yield as a beige powder, mp 173–174 °C.

^1^H NMR (400 MHz, DMSO-*d*_6_, ppm): δ 2.16–2.32 (m, 6H, CH_2_), 2.73–2.86 (m, 2H, CH_2_), 3.02–3.20 (m, 4H, CH_2_N), 3.51–3.63 (m, 6H, CH_2_N, CHSe), 3.74–4.02 (m, 10H, CHN, CH_2_O).

^13^C NMR (100 MHz, D_2_O, ppm): δ 22.5 (CHSe), 23.3 (CH_2_), 28.1 (CH_2_), 49.3 (CH_2_N), 63.8 (CH_2_O), 68.8 (CHN).

IR (KBr): 3728, 3417, 2913, 2676, 2604, 2458, 2249, 2073, 1634, 1416, 1267, 1181, 1127, 1019, 921, 653, 600, 518, 460 cm^−1^.

Anal. calcd for C_16_H_30_N_2_O_2_Br_2_Se (521.19): C 36.87, H 5.80, N 5.37, Br 30.66, Se 15.15%. Found: C 37.11, H 5.68, N 5.56, Br 30.87, Se 14.87%.

### 3.3. The Synthesis of Dihydrobromide Salts ***9**–**16*** from the Primary Amines

*2,6-Bis(allylamino)-9-selenabicyclo[3.3.1]nonane dihydrobromide* (**9**). A solution of allylamine (0.117 g, 2.05 mmol) in acetonitrile (1 mL) was added dropwise to a mixture of compound **2** (0.347 g, 1 mmol) and acetonitrile (9 mL) with stirring at room temperature. The reaction mixture was stirred overnight (20 h) at room temperature. Cold hexane (5 mL) was added to the mixture. The formed precipitate was filtered off, washed with cold acetonitrile and dried in a vacuum, giving the product (437 mg, 95% yield) mp 230–231 °C as a white powder.

^1^H NMR (400 MHz, D_2_O, ppm): δ 1.55–1.68 (m, 2H, CH_2_), 1.82–1.90 (m, 2H, CH_2_), 2.01–2.11 (m, 2H, CH_2_), 2.14–2.20 (m, 2H, CH_2_), 2.86–2.90 (m, 2H, CHSe), 3.24–3.34 (m, 4H, CH_2_N), 3.67–3.72 (m, 2H, CHN), 5.03–5.13 (m, 4H, CH=CH_2_), 5.43–5.54 (m, 2H, CH=CH_2_).

^13^C NMR (100 MHz, D_2_O, ppm): δ 25.0 (CH_2_), 25.2 (CHSe), 27.1 (CH_2_), 47.2 (CH_2_N), 59.5 (CHN), 123.8 (CH=CH_2_), 127.6 (CH=CH_2_).

Anal. calcd for C_14_H_26_N_2_Br_2_Se (461.14): C 36.46, H 5.68, N 6.07, Br 34.65, Se 17.12%. Found: C 36.63, H 5.81, N 5.89, Br 34.38, Se 16.86%.

*2,6-Bis(isopropylamino)-9-selenabicyclo[3.3.1]nonane dihydrobromide* (**10**) was obtained under the same conditions as compound **9** in 91% yield as a white powder, mp 230–231 °C.

^1^H NMR (400 MHz, D_2_O, ppm): δ 0.80 (d, 6H, CH_3_), 0.82 (d, 6H, CH_3_), 1.52–1.65 (m, 2H, CH_2_), 1.66–1.74 (m, 2H, CH_2_), 1.92–2.02 (m, 2H, CH_2_), 2.03–2.10 (m, 2H, CH_2_), 2.76–2.79 (m, 2H, CHSe), 3.03–3.13 (m, 2H, CHCH_3_), 3.66–3.72 (m, 2H, CHN).

^13^C NMR (100 MHz, D_2_O, ppm): δ 18.0 (CH_3_), 18.1 (CH_3_), 24.5 (CH_2_) 24.6 (CHSe), 26.6 (CH_2_), 47.2 (CH_3_CH), 56.6 (CH_2_CHN).

Anal. calcd for C_14_H_30_N_2_Br_2_Se (465.17): C 36.15, H 6.50, N 6.02, Br 34.35, Se 16.97%. Found: C 35.87, H 6.31, N 5.86, Br 34.12, Se 17.26%.

*2,6-Bis(butylamino)-9-selenabicyclo[3.3.1]nonane dihydrobromide* (**11**) was obtained under the same conditions as compound **9** in 96% yield as a white powder, mp 229–230 °C.

^1^H NMR (400 MHz, D_2_O, ppm): δ 0.44 (t, 6H, CH_3_), 0.87–0.96 (m, 4H, CH_2_), 1.14–1.23 (m, 4H, CH_2_), 1.50–1.63 (m, 2H, CH_2_), 1.74–1.85 (m, 2H, CH_2_), 1.95–2.16 (m, 4H, CH_2_), 2.56–2.70 (m, 4H, CH_2_N), 2.82–2.87 (m, 2H, CHSe), 3.62–3.67 (m, 2H, CHN).

^13^C NMR (100 MHz, D_2_O, ppm): δ 12.8 (CH_3_), 19.3 (CH_2_), 24.9 (CH_2_), 25.0 (CHSe), 27.0 (CH_2_), 27.6 (CH_2_), 45.0 (CH_2_N), 60.2 (CHN).

Anal. calcd for C_16_H_34_N_2_Br_2_Se (493.22): C 38.96, H 6.95, N 5.68, Br 32.40, Se 16.01%. Found: C 39.23, H 7.13, N 5.85, Br 32.61, Se 15.83%.

*2,6-Bis(isobutylamino)-9-selenabicyclo[3.3.1]nonane dihydrobromide* (**12**) was obtained under the same conditions as compound **9** in 93% yield as a white powder, mp 220–221 °C.

^1^H NMR (400 MHz, D_2_O, ppm): δ 0.47 (d, 6H, CH_3_), 0.49 (d, 6H, CH_3_), 1.45–1.54 (m, 2H, CH_3_CH), 1.55–1.65 (m, 2H, CH_2_), 1.73–1.80 (m, 2H, CH_2_), 1.92–2.02 (m, 2H, CH_2_), 2.04–2.12 (m, 2H, CH_2_), 2.40–2.49 (m, 4H, CH_2_N), 2.83–2.86 (m, 2H, CHSe), 3.60–3.66 (m, 2H, CHN).

^13^C NMR (100 MHz, D_2_O, ppm): δ 18.8 (CH_3_), 18.9 (CH_3_), 24.3 (CH_2_), 24.4 (CHSe), 25.2 (CH_2_), 26.6 (CH_3_CH), 51.9 (CH_2_N), 60.5 (CHN).

IR (KBr): 3996, 3737, 2922, 2789, 2531, 2419, 2339, 2212, 2101, 1958, 1846, 1631, 1578, 1440, 1283, 1157, 1004, 947, 882, 522, 437 cm^−1^.

Anal. calcd for C_16_H_34_N_2_Br_2_Se (493.22): C 38.96, H 6.95, N 5.68, Br 32.40, Se 16.01%. Found: C 39.01, H 6.99, N 5.63, Br 32.28, Se 16.11%.

*2,6-Bis(benzylamino)-9-selenabicyclo[3.3.1]nonane dihydrobromide* (**13**) was obtained under the same conditions as compound **9** in 94% yield, mp 205–206 °C.

^1^H NMR (400 MHz, CDCl_3_, ppm): δ 2.25–2.51 (m, 4H, CH_2_), 2.61–2.74 (m, 2H, CH_2_), 3.16–3.28 (m, 2H, CH_2_), 3.40–3.49 (m, 2H, CHSe), 3.94–4.00 (m, 2H, CHN), 4.14–4.24 (m, 4H, CH_2_N), 7.42–7.49 (m, 6H, CH_Ar_), 7.74–7.82 (m, 4H, CH_Ar_).

^13^C NMR (100 MHz, CDCl_3_, ppm): δ 25.4 (CH_2_), 25.7 (CHSe), 28.1 (CH_2_), 48.2 (CH_2_N), 59.2 (CHN), 129.0 (CH_Ar_), 129.3 (CH_Ar_), 130.2 (C_Ar_), 130.5 (CH_Ar_).

IR (KBr): 3437, 2946, 2740, 2345, 2051, 1982, 1580, 1455, 1291, 1211, 1159, 1101, 971, 850, 750, 696, 578, 485, 402 cm^−1^.

Anal. calcd for C_22_H_30_N_2_Br_2_Se (561.25): C 47.08, H 5.39, N 4.99, Br 28.47, Se 14.07%. Found: C 47.04, H 5.41, N 5.04, Br 28.32, Se 14.11%.

*2,6-Bis(phenylamino)-9-selenabicyclo[3.3.1]nonane dihydrobromide* (**14**) was obtained under the same conditions as compound **9** in 91% yield as a beige white powder, mp 212–215 °C.

^1^H NMR (400 MHz, DMSO-D_6_, ppm): δ 2.07–2.18 (m, 4H, CH_2_), 2.44–2.56 (m, 2H, CH_2_), 2.84–2.90 (m, 2H, CH_2_), 3.00–3.06 (m, 2H, CHSe), 4.23–4.31 (m, 2H, CHN), 7.31–7.36 (m, 2H, CH_Ar_), 7.45–7.53 (m, 8H, CH_Ar_).

^13^C NMR (100 MHz, DMSO-D_6_, ppm): δ 25.5 (CH_2_), 25.6 (CHSe), 27.5 (CH_2_), 63.3 (CHN), 122.3 (CH_Ar_), 127.5 (CH_Ar_), 130.0 (CH_Ar_), 135.7 (C_Ar_).

Anal. calcd for C_20_H_26_N_2_Br_2_Se (533.20): C 45.05, H 4.91, N 5.25, Br 29.97, Se 14.81%. Found: C 45.16, H 4.93, N 5.27, Br 29.84, Se 14.96%.

*2,6-Bis(tert-butylamino)-9-selenabicyclo[3.3.1]nonane dihydrobromide* (**15**) was obtained under the same conditions as compound **9** in 88% yield as a white powder, mp 238–239 °C.

^1^H NMR (400 MHz, D_2_O, ppm): δ 0.83 (s, 18H, CH_3_), 1.64–1.75 (m, 4H, CH_2_), 1.88–1.97 (m, 2H, CH_2_), 1.98–2.07 (m, 2H, CH_2_), 2.60–2.65 (m, 2H, CHSe), 3.68–3.75 (m, 2H, CHN).

^13^C NMR (100 MHz, D_2_O, ppm): δ 25.5 (CH_3_), 26.5 (CH_2_), 27.4 (CHSe), 27.5 (CH_2_), 57.5 (CN), 59.5 (CHN).

Anal. calcd for C_16_H_34_N_2_Br_2_Se (493.22): C 38.96, H 6.95, N 5.68, Br 32.40, Se 16.01%. Found: C 39.00, H 6.95, N 5.65, Br 32.32, Se 16.09%.

*2,6-Bis(2-vinyloxyethylamino)-9-selenabicyclo[3.3.1]nonane dihydrobromide* (**16**) was obtained under the same conditions as compound **9** in 94% yield as a yellowish powder, mp 177–178 °C.

^1^H NMR (400 MHz, D_2_O, ppm): δ 1.46–1.58 (m, 2H, CH_2_), 1.69–1.77 (m, 2H, CH_2_), 1.88–1.99 (m, 2H, CH_2_), 2.00–2.07 (m, 2H, CH_2_), 2.78–2.81 (m, 2H, CHSe), 2.87–2.91 (m, 4H, CH_2_N), 3.45–3.49 (m, 2H, CH_2_O), 3.61–3.65 (m, 4H, CHN, CH_2_=CH), 3.74–3.78 (m, 2H, CH_2_=CH), 5.92–5.98 (m, 2H, CH_2_=CH).

^13^C NMR (100 MHz, D_2_O, ppm): δ 24.8 (CH_2_), 24.8 (CHSe), 27.0 (CH_2_), 44.1 (CH_2_N), 60.5 (CHN), 62.7 (CH_2_O), 88.4 (CH_2_=CH), 150.6 (CH_2_=CH).

IR (KBr): 3342, 2941, 2817, 2452, 2204, 1870, 1619, 1547, 1445, 1399, 1325, 1195, 1067, 1003, 929, 821, 589, 475 cm^−1^.

Anal. calcd for C_16_H_30_N_2_Br_2_SeO_2_ (521.19): C 36.87, H 5.80, N 5.37, Br 30.66, Se 15.15%. Found: C 36.98, H 5.81, N 5.40, Br 30.54, Se 15.29%.

### 3.4. The Synthesis of the Free Base Compounds ***17**–**24*** from the Primary Amines

*2,6-Bis(2-vinyloxyethylamino)-9-selenabicyclo[3.3.1]nonane* (**17**). A solution of 2-vinyloxyethylamine (0.18 g, 2.07 mmol) in acetonitrile (1 mL) was added dropwise to a mixture of compound **2** (0.347 g, 1 mmol) and acetonitrile (11 mL) followed by the addition of powdered Na_2_CO_3_ (0.3 g, 3.5 mmol) with stirring at room temperature. The reaction mixture was stirred overnight (20 h) at room temperature. The main part of the solvent was removed on a rotary evaporator and water (10 mL) and methylene chloride (15 mL) were added. The aqueous phase was extracted with methylene chloride (3 × 15 mL). The organic phase was dried over Na_2_SO_4_ and the solvent was removed on a rotary evaporator. The residue was dried in a vacuum, giving the product (327 mg, 91% yield) as a grey oil.

^1^H NMR (400 MHz, CDCl_3_, ppm): δ 1.39–1.50 (m, 2H, CH_2_), 1.70–1.77 (m, 2H, CH_2_), 1.98–2.08 (m, 2H, CH_2_), 2.35–2.43 (m, 2H, CH_2_), 2.63–2.69 (m, 2H, CHSe), 2.74–2.80 (m, 4H, CH_2_N), 3.16–3.22 (m, 2H, CHN), 3.54–3.62 (m, 4H, CH_2_O), 3.79–3.83 (m, 2H, CH_2_=CH), 3.96–4.03 (m, 2H, CH_2_=CH), 6.25–6.32 (m, 2H, CH_2_=CH).

^13^C NMR (100 MHz, CDCl_3_, ppm): δ 27.3 (CH_2_), 29.0 (CHSe), 30.3 (CH_2_), 45.3 (CH_2_N), 59.4 (CHN), 67.2 (CH_2_O), 86.4 (CH_2_=CH), 151.3 (CH_2_=CH).

Anal. calcd for C_16_H_28_N_2_SeO_2_ (359.36): C 53.48, H 7.85, N 7.80, Se 21.97%. Found: C 53.54, H 8.01, N 7.67, Se 22.08%.

*2,6-Bis(isopropylamino)-9-selenabicyclo[3.3.1]nonane* (**18**) was obtained under similar conditions as compound **17** as a grey oil, yield: 95%.

^1^H NMR (400 MHz, CDCl_3_, ppm): δ 0.73 (d, 6H, CH_3_), 0.75 (d, 6H, CH_3_), 1.23–1.37 (m, 2H, CH_2_), 1.58–1.68 (m, 2H, CH_2_), 1.90–2.03 (m, 2H, CH_2_), 2.23–2.31 (m, 2H, CH_2_), 2.62–2.66 (m, 2H, CHSe), 2.67–2.73 (m, 2H, CHCH_3_), 3.13–3.20 (m, 2H, CHN).

^13^C NMR (100 MHz, CDCl_3_, ppm): δ 22.3 (CH_3_), 23.8 (CH_3_), 27.6 (CH_2_), 29.1 (CHSe), 31.0 (CH_2_), 44.1 (CH_3_CH), 56.2 (CH_2_CHN).

Anal. calcd for C_14_H_28_N_2_Se (303.34): C 55.43, H 9.30, N 9.23, Se 26.03%. Found: C 55.24, H 9.22, N 9.20, Se 25.81%.

*2,6-Bis(allylamino)-9-selenabicyclo[3.3.1]nonane* (**19**) was obtained under similar conditions as compound **17** as a grey oil, yield: 93%.

^1^H NMR (400 MHz, CDCl_3_, ppm): δ 1.35–1.45 (m, 2H, CH_2_), 1.64–1.72 (m, 2H, CH_2_), 1.95–2.03 (m, 2H, CH_2_), 2.32–2.39 (m, 2H, CH_2_), 2.69–2.74 (m, 2H, CHSe), 2.97–3.12 (m, 4H, CH_2_N), 3.14–3.19 (m, 2H, CHN), 4.83–4.98 (m, 4H, CH=CH_2_), 5.60–5.72 (m, 2H, CH=CH_2_).

^13^C NMR (100 MHz, CDCl_3_, ppm): δ 27.4 (CH_2_), 29.0 (CHSe), 30.3 (CH_2_), 49.0 (CH_2_N), 58.7 (CHN), 115.3 (CH=CH_2_), 136.8 (CH=CH_2_).

Anal. calcd for C_14_H_24_N_2_Se (299.31): C 56.18, H 8.08, N 9.36, Se 26.38%. Found: C 56.42, H 7.90, N 9.22, Se 26.41%.

*2,6-Bis(butylamino)-9-selenabicyclo[3.3.1]nonane* (**20**) was obtained under similar conditions as compound **17** as a grey oil, yield: 95%.

^1^H NMR (400 MHz, CDCl_3_, ppm): δ 0.71 (t, 6H, CH_3_), 1.09–1.19 (m, 4H, CH_2_), 1.20–1.31 (m, 4H, CH_2_), 1.36–1.48 (m, 2H, CH_2_), 1.67–1.76 (m, 2H, CH_2_), 1.95–2.07 (m, 2H, CH_2_), 2.31–2.42 (m, 4H, CH_2_N, CH_2_), 2.44–2.51 (m, 2H, CH_2_N), 2.74–2.79 (m, 2H, CHSe), 3.12–3.19 (m, 2H, CHN).

^13^C NMR (100 MHz, CDCl_3_, ppm): δ 13.7 (CH_3_), 20.2 (CH_2_), 27.5 (CH_2_), 29.1 (CHSe), 30.4 (CH_2_), 32.2 (CH_2_), 46.2 (CH_2_N), 59.6 (CHN).

Anal. calcd for C_16_H_32_N_2_Se (331.40): C 57.99, H 9.73, N 8.45, Se 23.83%. Found: C 58.04, H 9.77, N 8.41, Se 23.87%.

*2,6-Bis(isobutylamino)-9-selenabicyclo[3.3.1]nonane* (**21**) was obtained under similar conditions as compound **17** as a grey oil, yield: 92%.

^1^H NMR (400 MHz, CDCl_3_, ppm): δ 0.70 (d, 6H, CH_3_), 0.72 (d, 6H, CH_3_), 1.35–1.55 (m, 4H, CH_3_CH, CH_2_), 1.66–1.73 (m, 2H, CH_2_), 1.96–2.08 (m, 2H, CH_2_), 2.21–2.28 (m, 4H, CH_2_N), 2.35–2.41 (m, 2H, CH_2_), 2.72–2.76 (m, 2H, CHSe), 3.10–3.15 (m, 2H, CHN).

^13^C NMR (100 MHz, CDCl_3_, ppm): δ 20.4 (CH_3_), 20.5 (CH_3_), 27.5 (CH_2_), 28.4 (CH_2_), 29.3 (CHSe), 30.6 (CH_3_CH), 54.7 (CH_2_N), 59.6 (CHN). ^77^Se NMR (76 MHz, CDCl_3_): 303.8.

Anal. calcd for C_16_H_32_N_2_Se (331.40): C 57.99, H 9.73, N 8.45, Se 23.83%. Found: C 58.22, H 9.81, N 8.61, Se 23.68%.

*2,6-Bis(benzylamino)-9-selenabicyclo[3.3.1]nonane* (**22**) was obtained under similar conditions as compound **17** as a grey oil, yield: 96%.

^1^H NMR (400 MHz, CDCl_3_, ppm): δ 1.60–1.75 (m, 2H, CH_2_), 1.92–2.00 (m, 2H, CH_2_), 2.18–2.28 (m, 2H, CH_2_), 2.63–2.69 (m, 2H, CH_2_), 2.98–3.02 (m, 2H, CHSe), 3.42–3.48 (m, 2H, CHN), 3.74–3.89 (m, 4H, CH_2_N), 7.24–7.26 (m, 2H, CH_Ar_), 7.28–7.36 (m, 8H, CH_Ar_).

^13^C NMR (100 MHz, CDCl_3_, ppm): δ 27.6 (CH_2_), 29.2 (CHSe), 30.4 (CH_2_), 50.7 (CH_2_N), 59.1 (CHN), 126.7 (CH_Ar_), 127.8 (CH_Ar_), 128.1 (CH_Ar_), 140.4 (C_Ar_).

Anal. calcd for C_22_H_28_N_2_Se (399.43): C 66.15, H 7.07, N 7.01, Se 19.77%. Found: C 65.96, H 6.97, N 7.16, Se 19.89%.

*2,6-Bis(phenylamino)-9-selenabicyclo[3.3.1]nonane* (**23**) was obtained under similar conditions as compound **17** as a beige powder, mp 196–197 °C, yield: 95%.

^1^H NMR (400 MHz, CDCl_3_, ppm): δ 1.76–1.88 (m, 2H, CH_2_), 2.10–2.18 (m, 2H, CH_2_), 2.28–2.39 (m, 2H, CH_2_), 2.57–2.64 (m, 2H, CH_2_), 3.17–3.20 (m, 2H, CHSe), 4.27–4.34 (m, 2H, CHN), 6.54–6.69 (m, 4H, CH_Ar_), 6.72–6.78 (m, 2H, CH_Ar_), 7.18–7.25 (m, 4H, CH_Ar_).

^13^C NMR (100 MHz, CDCl_3_, ppm): δ 28.1 (CH_2_), 28.3 (CHSe), 30.5 (CH_2_), 55.7 (CHN), 113.9 (CH_Ar_), 118.1 (CH_Ar_), 129.6 (CH_Ar_), 146.0 (C_Ar_).

IR (KBr): 3417, 3366, 3014, 2910, 1926, 1600, 1502, 1429, 1309, 1260, 1152, 1103, 947, 893, 752, 693, 509 cm^−1^.

Anal. calcd for C_20_H_24_N_2_Se (371.38): C 64.68, H 6.51, N 7.54, Se 21.26%. Found: C 64.63, H 6.49, N 7.58, Se 21.31%.

*2,6-Bis(tert-butylamino)-9-selenabicyclo[3.3.1]nonane* (**24**) was obtained under similar conditions as compound **17** as a grey oil, yield: 91%.

^1^H NMR (400 MHz, CDCl_3_, ppm): δ 1.05 (s, 18H, CH_3_), 1.52–1.65 (m, 2H, CH_2_), 1.74–1.81 (m, 2H, CH_2_), 2.11–2.21 (m, 2H, CH_2_), 2.62–2.71 (m, 4H, CHSe, CH_2_), 3.33–3.40 (m, 2H, CHN).

^13^C NMR (100 MHz, CDCl_3_, ppm): δ 28.8 (CH_2_), 30.1 (CH_3_), 32.9 (CHSe), 33.3 (CH_2_), 51.2 (CN), 54.8 (CHN).

Anal. calcd for C_16_H_32_N_2_Se (331.40): C 57.99, H 9.73, N 8.45, Se 23.83%. Found: C 58.14, H 9.90, N 8.59, Se 24.04%.

### 3.5. The Synthesis of Free Base Compounds ***25**–**30*** from the Secondary Amines

*2,6-Bis(diethylamino)-9-selenabicyclo[3.3.1]nonane* (**25**). A solution of diethylamine (0.131 g, 1.8 mmol) in acetonitrile (1 mL) was added dropwise to a mixture of compound **2** (0.3 g, 0.86 mmol) and acetonitrile (9 mL) followed by the addition of powdered Na_2_CO_3_ (0.28 g, 3.26 mmol) with stirring at room temperature. The reaction mixture was stirred overnight (20 h) at room temperature. The main part of the solvent was removed on a rotary evaporator and water (10 mL) and methylene chloride (15 mL) were added. The aqueous phase was extracted with methylene chloride (3 × 15 mL). The organic phase was dried over Na_2_SO_4_ and the solvent was removed on a rotary evaporator. The residue was dried in a vacuum, giving the product (278 mg, 97% yield) as a grey oil.

^1^H NMR (400 MHz, CDCl_3_, ppm): δ 0.88 (t, 12H, CH_3_), 1.71–1.79 (m, 2H, CH_2_), 1.88–1.99 (m, 2H, CH_2_), 2.16–2.26 (m, 2H, CH_2_), 2.46–2.66 (m, 10H, CH_2_N, CH_2_), 2.91–2.94 (m, 2H, CHSe), 3.28–3.33 (m, 2H, CHN).

^13^C NMR (100 MHz, CDCl_3_, ppm): δ 13.0 (CH_3_), 27.2 (CH_2_) 29.2 (CHSe), 30.6 (CH_2_), 43.3 (CH_2_N), 62.0 (CHN).

Anal. calcd for C_16_H_32_N_2_Se (331.40): C 57.99, H 9.73, N 8.45, Se 23.83%. Found: C 58.32, H 9.92, N 8.62, Se 23.64%.

*2,6-Bis(diallylamino)-9-selenabicyclo[3.3.1]nonane* (**26**) was obtained under similar conditions as compound **25** as a grey oil, yield: 94%.

^1^H NMR (400 MHz, CDCl_3_, ppm): δ 1.81–1.90 (m, 2H, CH_2_), 1.94–2.07 (m, 2H, CH_2_), 2.24–2.34 (m, 2H, CH_2_), 2.62–2.69 (m, 2H, CH_2_), 2.99–3.03 (m, 2H, CHSe), 3.16–3.30 (m, 8H, CH_2_N), 3.43–3.49 (m, 2H, CHN), 5.02–5.15 (m, 8H, CH=CH_2_), 5.72–5.84 (m, 4H, CH=CH_2_).

^13^C NMR (100 MHz, CDCl_3_, ppm): δ 27.0 (CH_2_), 29.3 (CHSe), 30.7 (CH_2_), 53.0 (CH_2_N), 61.9 (CHN), 116.4 (CH=CH_2_), 136.6 (CH=CH_2_).

Anal. calcd for C_20_H_32_N_2_Se (379.44): C 63.31, H 8.50, N 7.38, Se 20.81%. Found: C 63.19, H 8.62, N 7.54, Se 21.01%.

*2,6-Bis(diisopropyl)-9-selenabicyclo[3.3.1]nonane* (**27**) was obtained under similar conditions as compound **25** as a grey oil, yield: 95%.

^1^H NMR (400 MHz, CDCl_3_, ppm): δ 1.01 (d, 12H, CH_3_), 1.06 (d, 12H, CH_3_), 1.69–1.75 (m, 2H, CH_2_), 2.26–2.37 (m, 2H, CH_2_), 2.42–2.51 (m, 2H, CH_2_), 2.65–2.69 (m, 2H, CHSe), 2.69–2.74 (m, 2H, CH_2_), 3.16–3.28 (m, 4H, CHCH_3_), 3.63–3.68 (m, 2H, CHN).

^13^C NMR (100 MHz, CDCl_3_, ppm): δ 22.4 (CH_3_), 24.4 (CH_3_), 29.6 (CH_2_), 32.7 (CHSe), 35.7 (CH_2_), 45.8 (CHCH_3_), 57.4 (CHN).

IR (film): 3718, 3434, 2960, 1611, 1457, 1360, 1183, 1135, 1083, 1014, 909, 857, 653, 569, 493 cm^−1^.

Anal. calcd for C_20_H_40_N_2_Se (387.50): C 61.99, H 10.40, N 7.23, Se 20.38%. Found: C 62.14, H 10.48, N 7.11, Se 20.43%.

*2,6-Bis(1-pyrrolidinyl)-9-selenabicyclo[3.3.1]nonane* (**28**) was obtained under similar conditions as compound **25** as a white powder, mp 109–110 °C, yield: 95%.

^1^H NMR (400 MHz, CDCl_3_, ppm): δ 1.72–1.79 (m, 8H, CH_2_CH_2_N), 1.90–2.00 (m, 2H, CH_2_), 2.14–2.25 (m, 2H, CH_2_), 2.51–2.57 (m, 8H, CH_2_N), 2.62–2.68 (m, 2H, CH_2_), 2.74–2.80 (m, 2H, CHN), 2.99–3.02 (m, 2H, CHSe).

^13^C NMR (100 MHz, CDCl_3_, ppm): δ 23.2 (CH_2_CH_2_N), 28.4 (CH_2_), 29.6 (CHSe), 29.7 (CH_2_), 51.9 (CH_2_N), 67.7 (CHN). ^77^Se NMR (76 MHz, CDCl_3_, ppm): δ 347.1.

IR (KBr): 3434, 2932, 2775, 1631, 1424, 1354, 1305, 1257, 1197, 1125, 1040, 948, 893, 592 cm^−1^.

Anal. calcd for C_16_H_28_N_2_Se (327.37): C 58.70, H 8.62, N 8.56, Se 24.12%. Found: C 58.73, H 8.61, N 8.58, Se 24.06%.

*2,6-Bis(1-piperidinyl)-9-selenabicyclo[3.3.1]nonane* (**29**) was obtained under similar conditions as compound **25** as a white powder, mp 105–106 °C, yield: 93%.

^1^H NMR (400 MHz, CDCl_3_, ppm): δ 1.40–1.47 (m, 4H, CH_2_CH_2_CH_2_N), 1.52–1.61 (m, 8H, CH_2_CH_2_N), 1.86–2.07 (m, 4H, CH_2_), 2.23–2.33 (m, 2H, CH_2_), 2.52–2.62 (m, 8H, CH_2_N), 2.69–2.75 (m, 2H, CH_2_), 3.03–3.08 (m, 2H, CHN), 3.14–3.18 (m, 2H, CHSe).

^13^C NMR (100 MHz, CDCl_3_, ppm): δ 24.9 (CH_2_CH_2_CH_2_N), 26.6 (CH_2_CH_2_N), 27.2 (CH_2_), 28.6 (CHSe), 30.7 (CH_2_), 51.3 (CH_2_N), 66.8 (CHN).

IR (KBr): 3991, 2929, 2850, 2786, 2523, 1655, 1440, 1376, 1306, 1259, 1099, 1036, 978, 900, 786, 732, 617 cm^−1^.

Anal. calcd for C_18_H_32_N_2_Se (355.42): C 60.83, H 9.07, N 7.88, Se 22.22%. Found: C 60.58, H 8.97, N 8.04, Se 22.01%.

*2,6-Dimorpholinyl-9-selenabicyclo[3.3.1]nonane* (**30**) was obtained under similar conditions as compound **25** as a yellowish powder, mp 139–140 °C, yield: 91%.

^1^H NMR (400 MHz, CDCl_3_, ppm): δ 1.71–1.84 (m, 2H, CH_2_), 1.87–1.95 (m, 2H, CH_2_), 2.12–2.21 (m, 2H, CH_2_), 2.50–2.53 (m, 8H, CH_2_N), 2.62–2.69 (m, 2H, CH_2_), 2.84–2.90 (m, 2H, CHN), 3.05–3.08 (m, 2H, CHSe), 3.64–3.69 (m, 8H, CH_2_O).

^13^C NMR (100 MHz, CDCl_3_, ppm): δ 27.0 (CH_2_), 27.3 (CHSe), 29.1 (CH_2_), 50.7 (CH_2_N), 65.9 (CHN), 67.4 (CH_2_O).

IR (KBr): 2945, 2857, 2794, 1638, 1449, 1273, 1191, 1115, 987, 925, 878, 792, 712, 657, 612, 513 cm^−1^.

Anal. calcd for C_16_H_28_N_2_O_2_Se (359.36): C 53.48, H 7.85, N 7.80, Se 21.97%. Found: C 53.72, H 7.98, N 7.66, Se 22.10%.

### 3.6. The Synthesis of Diamino Derivative ***31*** 

*2,6-Diamino-9-selenabicyclo[3.3.1]nonane* (**31**). To a solution of diazide **32** [67] (271 mg, 1 mmol) in THF (4 mL), PPh_3_ was added (1040 mg, 4 mmol) at room temperature. After stirring at room temperature for 2 h and at 40–50 °C for 6 h, H_2_O (360 mg) was added at the same temperature. The mixture was stirred at 40–50 °C for 8 h and 50–60 °C for 2 h; the solvent was removed on a rotary evaporator and the residue was subjected to column chromatography (eluent: hexane, then hexane/chloroform 8:1, then hexane/chloroform 4:1), giving the product (197 mg, 90% yield) as a colorless oil.

^1^H NMR (400 MHz, CDCl_3_, ppm): δ 1.41 (s, 4H, NH_2_), 1.57–1.69 (m, 2H, CH_2_), 1.91–1.97 (m, 2H, CH_2_), 2.21–2.31 (m, 2H, CH_2_), 2.61–2.67 (m, 2H, CH_2_), 2.68–2.71 (m, 2H, CHSe), 3.60–3.65 (m, 2H, CHN).

^13^C NMR (100 MHz, CDCl_3_, ppm): δ 27.1 (CH_2_), 32.2 (CHSe), 32.8 (CH_2_), 53.7 (CHN).

^77^Se NMR (76 MHz, CDCl_3_, ppm): δ 309.3.

IR (film): 3351, 3280, 2924, 2687, 2214, 1583, 1480, 1362, 1265, 1092, 909, 731, 643, 491 cm^−1^.

Anal. calcd for C_8_H_16_N_2_Se (219.19): C 43.84, H 7.36, N 12.78, Se 36.02%. Found: C 43.69, H 7.26, N 12.89, Se 35.74%.

*The Synthesis of diamine* **31** *from compound* **2**. A solution of compound **2** (0.347 g, 1 mmol) in methylene chloride (10 mL) was added to a 10% solution of ammonia (15 mL) containing K_2_CO_3_ (1 g). The reaction mixture was stirred for 48 h at room temperature. The organic phase was separated, dried over Na_2_SO_4_, and the solvent was removed on a rotary evaporator. The residue was subjected to column chromatography (eluent: hexane, then hexane/chloroform 8:1, then hexane/chloroform 4:1), giving the product (178 mg, 81% yield).

### 3.7. The Synthesis of Selenabicyclo[3.3.1]nonene-2 Derivatives ***36**–**42*** 

*6-Bromo-9-selenabicyclo[3.3.1]nonene-2 (***36***).* A solution of compound **2** (0.694 g, 2 mmol) in acetonitrile (10 mL) was heated at 55–65 °C with stirring for 12 h. Acetonitrile was removed on a rotary evaporator. The residue was analyzed by NMR, which showed an 80% conversion of compound **2**, and subjected to column chromatography (eluent: hexane, then hexane/chloroform 8:1), giving the product (383 mg, 90% yield based on consumed compound **2**).

^1^H NMR (400 MHz, CDCl_3_, ppm): δ 2.10–2.17 (m, 1H, CH_2_), 2.22–2.29 (m, 2H, CH_2_), 2.31–2.40 (m, 1H, CH_2_), 2.58–2.71 (m, 2H, CH_2_), 3.39–3.44 (m, 2H, CHSe), 5.08–5.14 (m, 1H, CHBr), 5.86–5.95 (m, 1H, CH=CH), 5.96–6.02 (m, 1H, CH=CH).

^13^C NMR (100 MHz, CDCl_3_, ppm): δ 25.7 (CH_2_), 28.0 (CH_2_), 30.9 (CHSe), 33.7(CHSe), 36.0 (CH_2_), 59.9 (CHBr), 128.0 (CH=CH), 132.0 (CH=CH). ^77^Se NMR (76 MHz, CDCl_3_, ppm): δ 336.1.

Anal. calcd for C_8_H_11_BrSe (266.04): C 36.12, H 4.17, Br 30.03, Se 29.68%. Found: C 36.14, H 4.18, Br 29.97, Se 29.81%.

*6-Azido-9-selenabicyclo[3.3.1]non-2-ene* (**37**). A solution of sodium azide (1 g, 15.4 mmol) in water (5.3 mL) was added dropwise to a mixture of compound **36** (0.532 g, 2 mmol) and acetonitrile (8 mL) with stirring at room temperature. The reaction mixture was stirred overnight (16 h) at room temperature. Acetonitrile was removed on a rotary evaporator and the residue was extracted with methylene chloride (3 × 10 mL). The organic phase was dried over CaCl_2_, the solvent was removed on a rotary evaporator and the residue was dried in a vacuum, giving the product (433 mg, 95% yield) as a grey oil.

^1^H NMR (400 MHz, CDCl_3_, ppm): δ 1.67–1.79 (m, 1H, CH_2_), 1.85–1.93 (m, 1H, CH_2_), 2.14–2.32 (m, 3H, CH_2_), 2.51–2.60 (m, 1H, CH_2_), 3.20–3.25 (m, 1H, CHSe), 3.32–3.37 (m, 1H, CHSe), 4.21–4.27 (m, 1H, CHN), 5.87–5.94 (m, 2H, CH=CH).

^13^C NMR (100 MHz, CDCl_3_, ppm): δ 24.5 (CH_2_), 25.6 (CH_2_) 27.3 (CHSe), 30.3 (CHSe), 34.0 (CH_2_), 65.4 (CHN), 128.4 (HC=CH), 131.9 (HC=CH). ^77^Se NMR (76 MHz, CDCl_3_, ppm): δ 330.2.

IR (film): 3309, 2918, 2474, 2089, 1648, 1433, 1365, 1254, 1157, 1112, 1001, 949, 883, 700, 617, 557 cm^−1^.

Anal. calcd for C_8_H_11_N_3_Se (228.15): C 42.12, H 4.86, N 18.42, Se 34.61%. Found: C 42.17, H 4.88, N 18.36, Se 34.71%.

*6-Amino-9-selenabicyclo[3.3.1]nonene-2* (**38**). To a solution of azide **37** (228 mg, 1 mmol) in THF (3 mL), PPh_3_was added (524 mg, 2 mmol) at room temperature. After stirring at room temperature for 4 h and at 40–50 °C for 4 h, H_2_O (180 mg) was added at the same temperature. The mixture was stirred at room temperature for 8 h and 50–60 °C for 2 h, the solvent was removed on a rotary evaporator and the residue was subjected to column chromatography (eluent: hexane, then hexane/chloroform 8:1, then hexane/chloroform 4:1), giving the product (178 mg, 88% yield).

^1^H NMR (400 MHz, CDCl_3_, ppm): δ 1.45 (s, 2H, NH_2_), 1.64–1.76 (m, 2H, CH_2_), 1.96–2.07 (m, 2H, CH_2_), 2.19–2.28 (m, 2H, CH_2_), 2.59–2.66 (m, 2H, CH_2_), 2.65–2.72 (m, 2H, CHSe), 3.58–3.64 (m, 1H, CHN), 5.87–5.94 (m, 2H, CH=CH.

^13^C NMR (100 MHz, CDCl_3_, ppm): δ 25.2 (CH_2_), 25.9 (CH_2_), 27.8 (CH_2_), 33.8 (CHSe), 34.0 (CHSe), 54.9 (CHN), 128.4 (HC=CH), 131.9 (HC=CH). ^77^Se NMR (76 MHz, CDCl_3_): 276.4.

IR (film): 3351, 3011, 2925, 1958, 1647, 1571, 1448, 1339, 1278, 1117, 946, 867, 694, 648, 556 cm^−1^.

Anal. calcd for C_8_H_13_NSe (202.156): C 47.53, H 6.48, N 6.93, Se 39.06%. Found: C 47.79, H 6.29, N 7.08, Se 38.84%.

*6-Methoxy-9-selenabicyclo[3.3.1]nonene-2* (**39**). Methanol (0.4 mL) and NaHCO_3_ (0.05 g) were added to a solution of bromide **36** (133 mg, 0.5 mmol) in acetonitrile (2 mL). The mixture was stirred overnight (20 h) at room temperature and filtered. The solvent was on a rotary evaporator and the residue was dried in a vacuum to give the product as a colorless oil (yield: 107 mg, 99%), which did not require additional purification.

^1^H NMR (400 MHz, CDCl_3_, ppm): δ 1.54–1.65 (m, 1H, CH_2_), 1.83–1.91 (m, 1H, CH_2_), 2.16–2.31 (m, 3H, CH_2_), 2.43–2.52 (m, 1H, CH_2_), 3.28–3.33 (m, 1H, CHSe), 3.35–3.42 (m, 4H, CHSe, CH_3_), 3.78–3.84 (m, 1H, CHO), 5.85–5.92 (m, 2H, CH=CH).

^13^C NMR (100 MHz, CDCl_3_, ppm): δ 25.1 (CH_2_), 25.8 (CH_2_) 26.5 (CHSe), 29.3 (CHSe), 34.9 (CH_2_), 55.9 (CH_3_), 82.4 (CHO), 128.5 (HC=CH), 132.1 (HC=CH). ^77^Se NMR (76 MHz, CDCl_3_, ppm): δ 257.1.

Anal. calcd for C_9_H_14_OSe (217.17): C 49.77, H 6.50, Se 36.36%. Found: C 49.69, H 6.32, Se 36.41%.

*6-Ethoxy-9-selenabicyclo[3.3.1]nonene-2* (**40**) was obtained under similar conditions as compound **39** as a colorless oil, yield: 97%.

^1^H NMR (400 MHz, CDCl_3_, ppm): δ 1.54–1.65 (m, 1H, CH_2_), 1.83–1.91 (m, 1H, CH_2_), 2.16–2.31 (m, 3H, CH_2_), 2.43–2.52 (m, 1H, CH_2_), 3.28–3.33 (m, 1H, CHSe), 3.35–3.42 (m, 4H, CHSe, CH_3_), 3.78–3.84 (m, 1H, CHO), 5.84–5.92 (m, 2H, CH=CH).

^13^C NMR (100 MHz, CDCl_3_, ppm): δ 15.9 (CH_3_), 25.5 (CH_2_), 25.8 (CH_2_) 26.6 (CHSe), 30.0 (CHSe), 35.0 (CH_2_), 63.5 (CH_2_O), 80.7 (CHO), 128.4 (HC=CH), 132.1 (HC=CH). ^77^Se NMR (76 MHz, CDCl_3_, ppm): δ 259.4.

Anal. calcd for C_10_H_16_OSe (231.19): C 51.95, H 6.98, Se 34.15%. Found: C 51.82, H 6.89, Se 34.17%.

*6-Isobutoxy-9-selenabicyclo[3.3.1]nonene-2* (**41**) was obtained under similar conditions as compound **39** as a colorless oil, yield: 94%.

^1^H NMR (400 MHz, CDCl_3_): δ 0.89–0.92 (m, 6H, CH_3_), 1.55–1.67 (m, 1H, CH_2_), 1.76–1.88 (m, 2H, CH_2,_ CHCH_3_), 2.14–2.36 (m, 3H, CH_2_), 2.42–2.50 (m, 1H, CH_2_), 3.18–3.22 (m, 1H, CHSe), 3.27–3.34 (m, 3H, CHSe, CH_2_O), 3.81–3.87 (m, 1H, CHO), 5.83–5.91 (m, 2H, CH=CH).

^13^C NMR (100 MHz, CDCl_3_): δ 19.5 (CH_3_), 19.6 (CH_3_), 25.4 (CH_2_), 25.8 (CH_2_) 26.6 (CHSe), 28.9 (CH_3_CH), 30.0 (CHSe), 35.0 (CH_2_), 75.3 (CH_2_O), 80.9 (CHO), 128.4 (HC=CH), 132.1 (HC=CH). ^77^Se NMR (76 MHz, CDCl_3_, ppm): δ 257.5.

Anal. calcd for C_12_H_20_OSe (259.25): C 55.59, H 7.78, O 6.17, Se 30.46%. Found: C 55.64, H 7.80, Se 30.58%.

*6-Butoxy-9-selenabicyclo[3.3.1]nonene-2* (**42**) was obtained under similar conditions as compound **39** as a colorless oil, yield: 95%.

^1^H NMR (400 MHz, CDCl_3_): δ 0.92 (t, 3H, CH_3_), 1.33–1.44 (m, 2H, CH_2_), 1.49–1.60 (m, 2H, CH_2_), 1.60–1.67 (m, 1H, CH_2_), 1.80–1.86 (m, 1H, CH_2_), 2.14–2.36 (m, 3H, CH_2_), 2.42–2.50 (m, 1H, CH_2_), 3.26–3.30 (m, 1H, CHSe), 3.30–3.34 (m, 1H, CHSe), 3.41–3.46 (m, 2H, CH_2_O), 3.53–3.59 (m, 2H, CH_2_O), 3.84–3.89 (m, 1H, CHO), 5.84–5.91 (m, 2H, CH=CH).

^13^C NMR (100 MHz, CDCl_3_): δ 14.0 (CH_3_), 19.5 (CH_2_), 25.5 (CH_2_) 25.8 (CHSe), 26.6 (CH_2_), 30.0 (CHSe), 32.4 (CH_2_), 35.0 (CH_2_), 68.1 (CH_2_O), 80.8 (CHO), 128.4 (HC=CH), 132.1 (HC=CH). ^77^Se NMR (76 MHz, CDCl_3_, ppm): δ 330.6.

Anal. calcd for C_12_H_20_OSe (259.25): C 55.59, H 7.78, Se 30.46%. Found: C 55.56, H 7.74, Se 30.59%.

## 4. Conclusions

The efficient and selective synthesis of 6-bromo-9-selenabicyclo[3.3.1]nonene-2 was developed by the remarkable reaction of 2,6-dibromo-9-selenabicyclo[3.3.1]nonane with acetonitrile. Azido-, amino- and alkoxy-9-selenabicyclo[3.3.1]nonene-2 derivatives were obtained in 88–99% yields based on the azidation and alkoxylation reactions of 6-bromo-9-selenabicyclo[3.3.1]nonene-2. The 9-selenabicyclo[3.3.1]nonene-2 derivatives are a new class of organoselenium heterocyclic compounds and are prospective intermediates for organic synthesis.

The methods of amination of 2,6-dibromo-9-selenabicyclo[3.3.1]nonane are characterized by high yields of target products (88–98%), high selectivity and simple reaction conditions (room temperature), broad-scope reactions (ammonia, both primary and secondary amines with various substituents (Et, i-Pr, Bu, i-Bu, t-Bu, PhCH_2,_ Ph, morpholine, piperidine, pyrrolidine and 2-vinyloxyethylamine) and convenient isolation procedures. The products were isolated from the reaction mixture by filtration (dihydrobromide salts) or by solvent removing (free base diamino derivatives) and did not require additional purifications. These methods meet the criteria of click chemistry.

Four water-soluble dihydrobromide compounds, containing diethylamino, isopropylamino, phenylamino and pyrrolidine substituents, were found to exhibit high GPx mimetic activity.

## Data Availability

Data are contained within the article and Supplementary Materials.

## Supplementary Materials

The supporting information can be downloaded at: https://www.mdpi.com/article/10.3390/ijms242417485/s1.
