# Supplementary material for: Selenium Dibromide Click Chemistry: The Efficient Synthesis of Novel Selenabicyclo[3.3.1]nonene-2 and -nonane Derivatives
Source: Int J Mol Sci. 2023 Dec 14;24(24):17485. doi: 10.3390/ijms242417485 (PMC10744190; doi:10.3390/ijms242417485)

Supplementary Materials

Selenium Dibromide Click Chemistry: The Efficient Synthesis of Novel Selenabicyclo[3.3.1]nonene-2 and -nonane Derivatives

Maxim V. Musalov *, Svetlana V. Amosova and Vladimir A. Potapov

A. E. Favorsky Irkutsk Institute of Chemistry, Siberian Division of The Russian Academy of Sciences, 1 Favorsky Str., Irkutsk 664033, Russia; amosova@irioch.irk.ru (S.V.A.); v.a.potapov@mail.ru (V.A.P.)

* Correspondence: musalov_maxim@irioch.irk.ru

Table of Contents

Experimental (General Information) 2

^{1}H and ^{13}C-NMR Spectra of All Products 3-37

Experimental (General Information)

The ^{1}H (400.1 MHz) and ^{13}C (100.6 MHz) NMR spectra were recorded on a Bruker DPX-400 spectrometer (Bruker BioSpin GmbH, Rheinstetten, Germany) in $CDCl_3$ (referred to the residual solvent peaks, δ = 7.27 and 77.16 ppm for ^{1}H and ^{13}C NMR, respectively), DMSO-$\boldsymbol{d_6}$ (referred to the residual solvent peaks, δ = 2.50 and 39.50 ppm for ^{1}H and ^{13}C NMR, respectively) and D_2O (referred to the external standard HMDSO, δ = 0.055 and 1.97 ppm for ^{1}H and ^{13}CNMR, respectively).

^{1}H and ^{13}C-NMR Spectra of All Products

^{1}H- and ^{13}C-NMR spectra of compound 4 (D_2O)

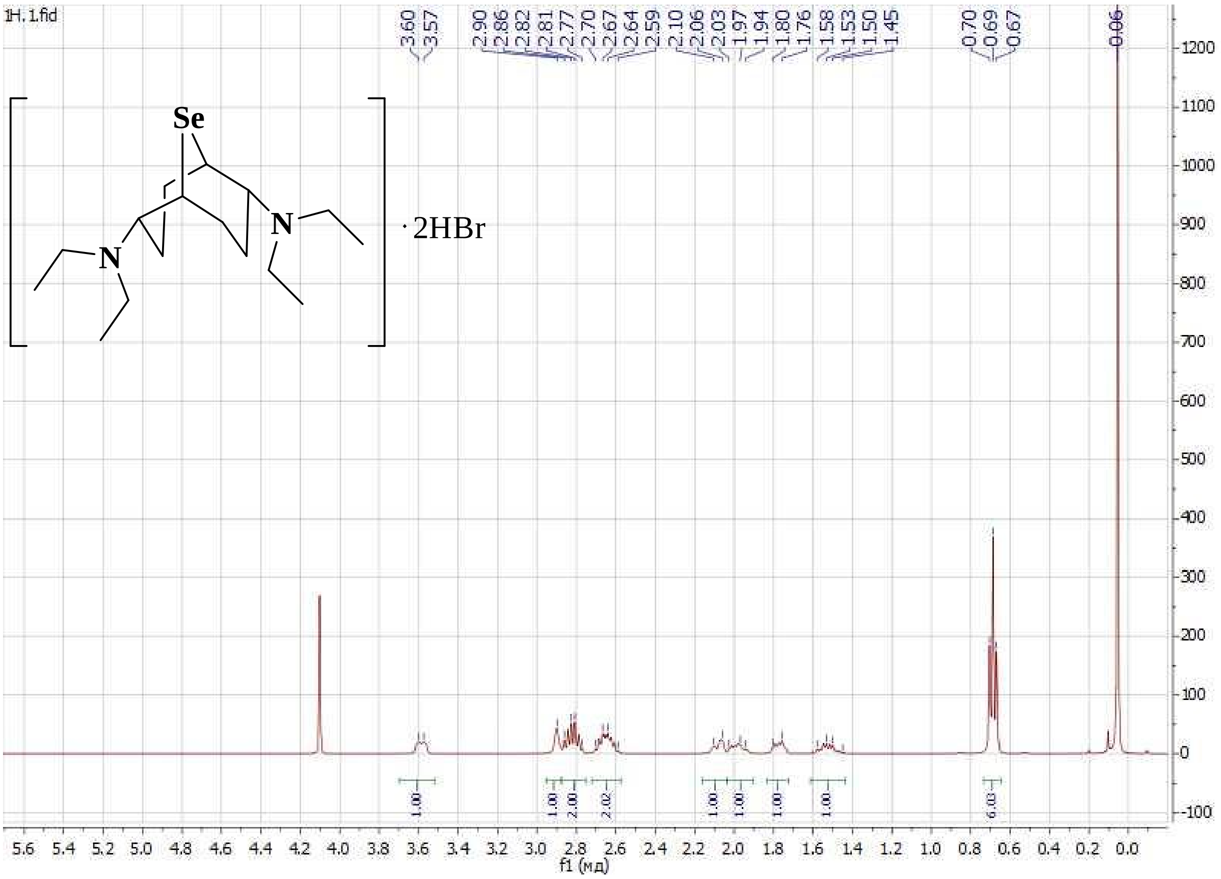

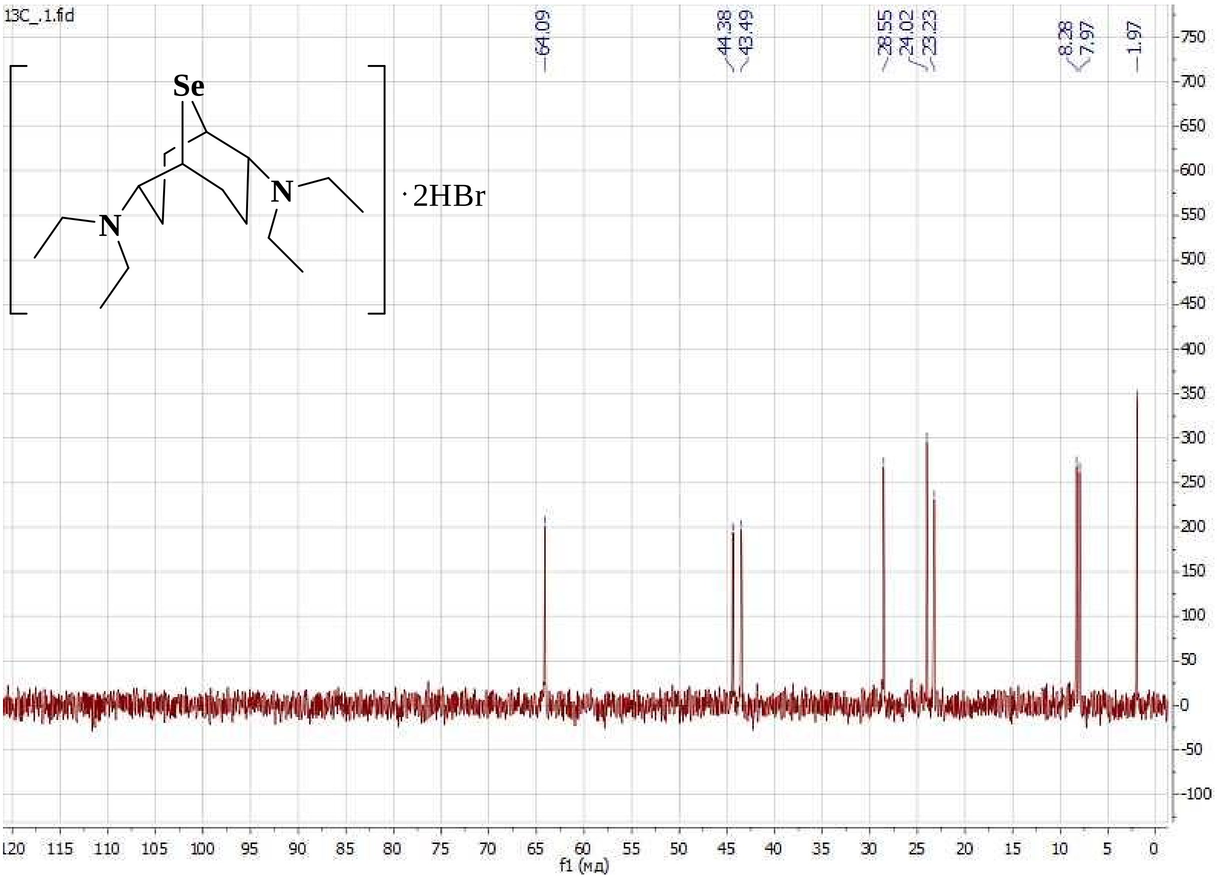

^{1}H- and ^{13}C-NMR spectra of compound 5 (D_2O)

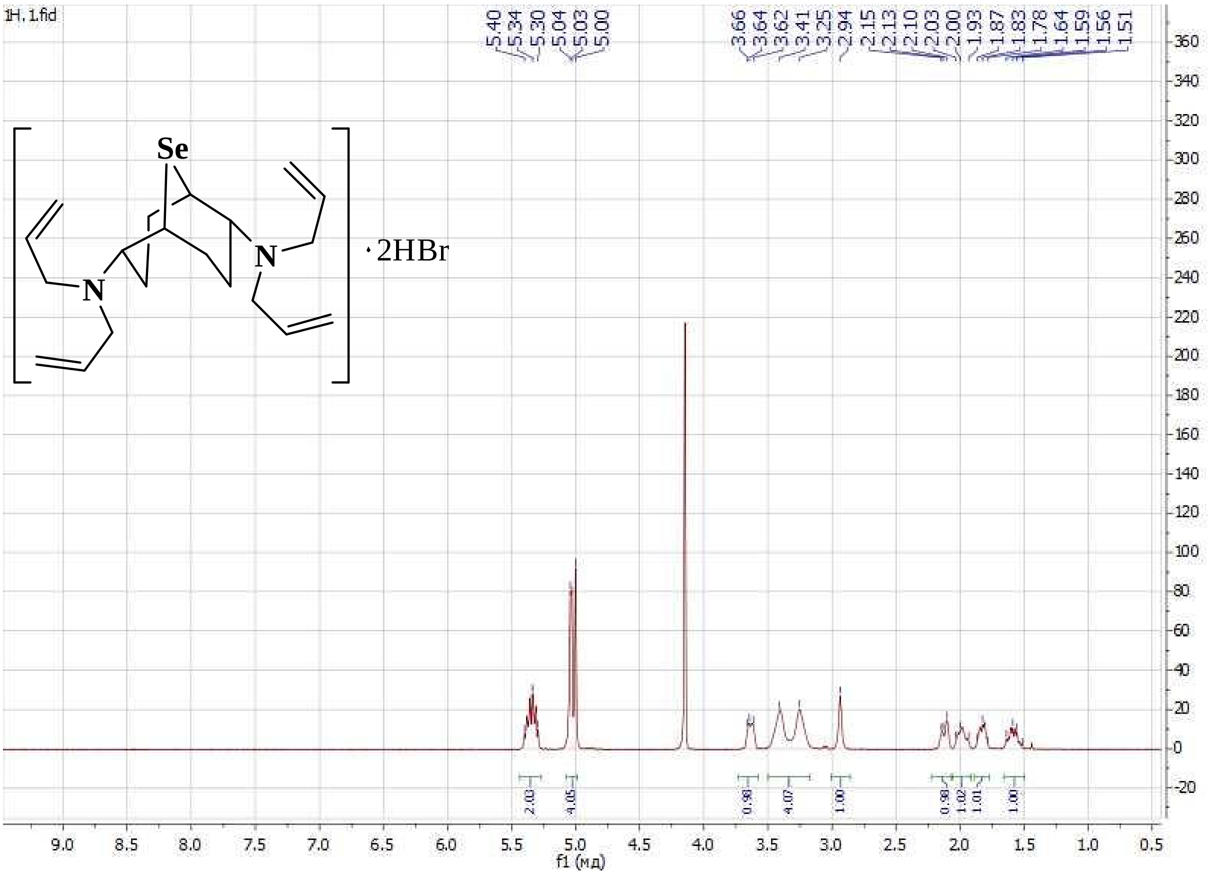

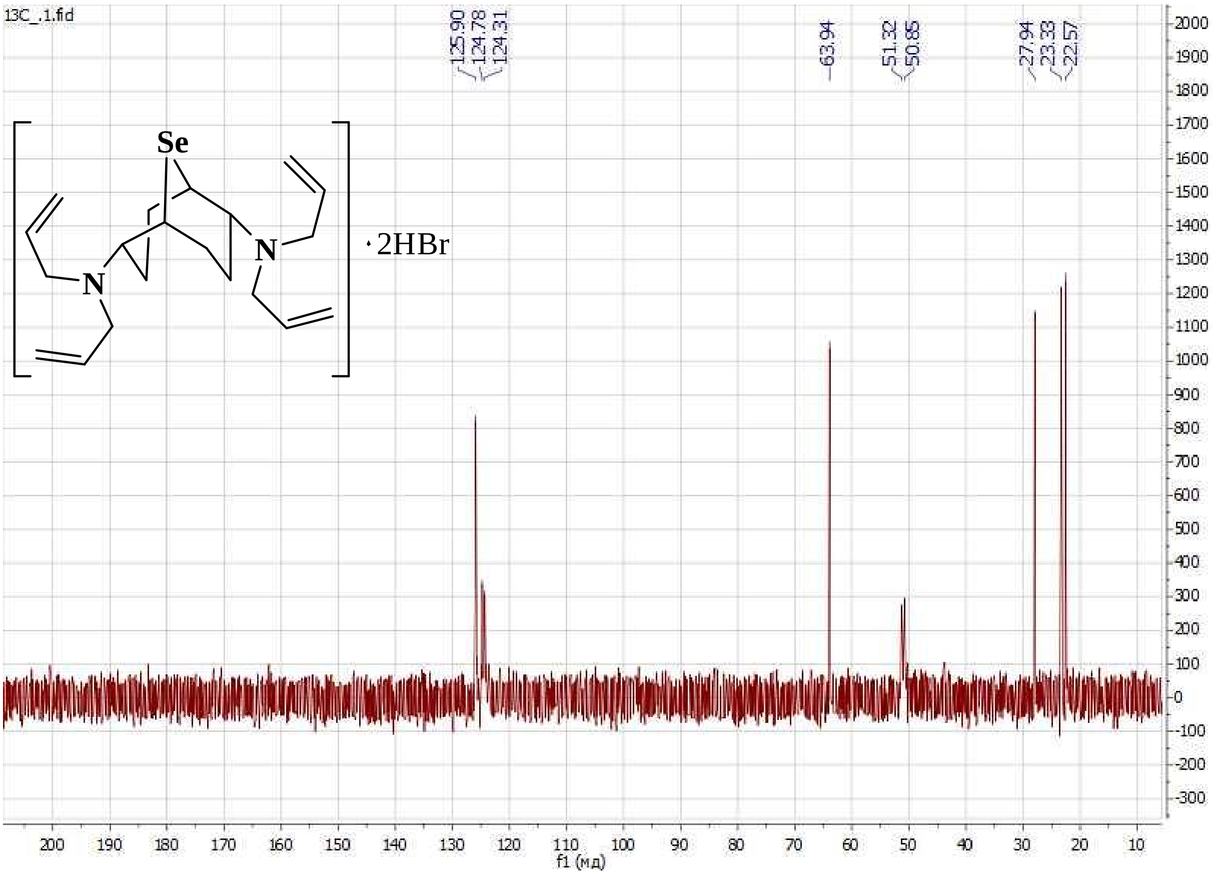

^{1}H- and ^{13}C-NMR spectra of compound 6 (D_2O)

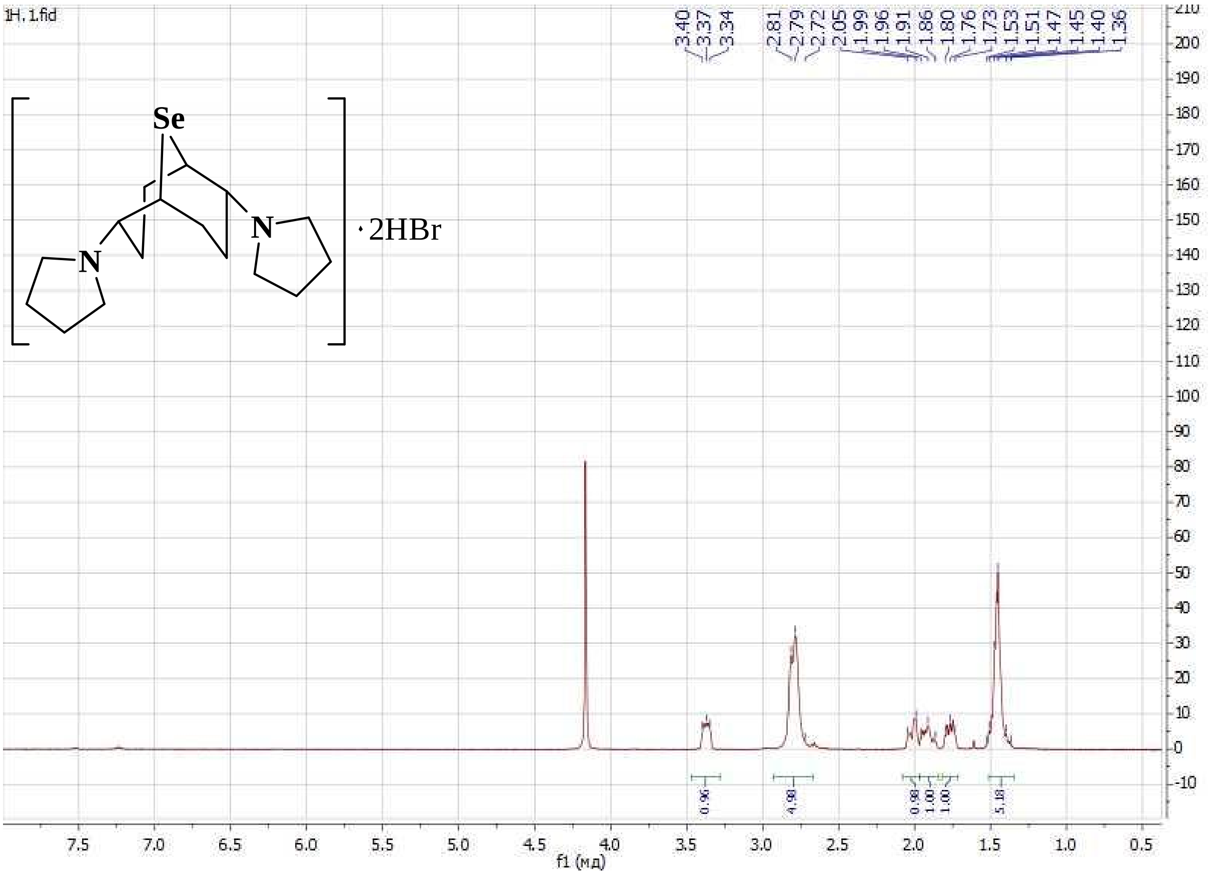

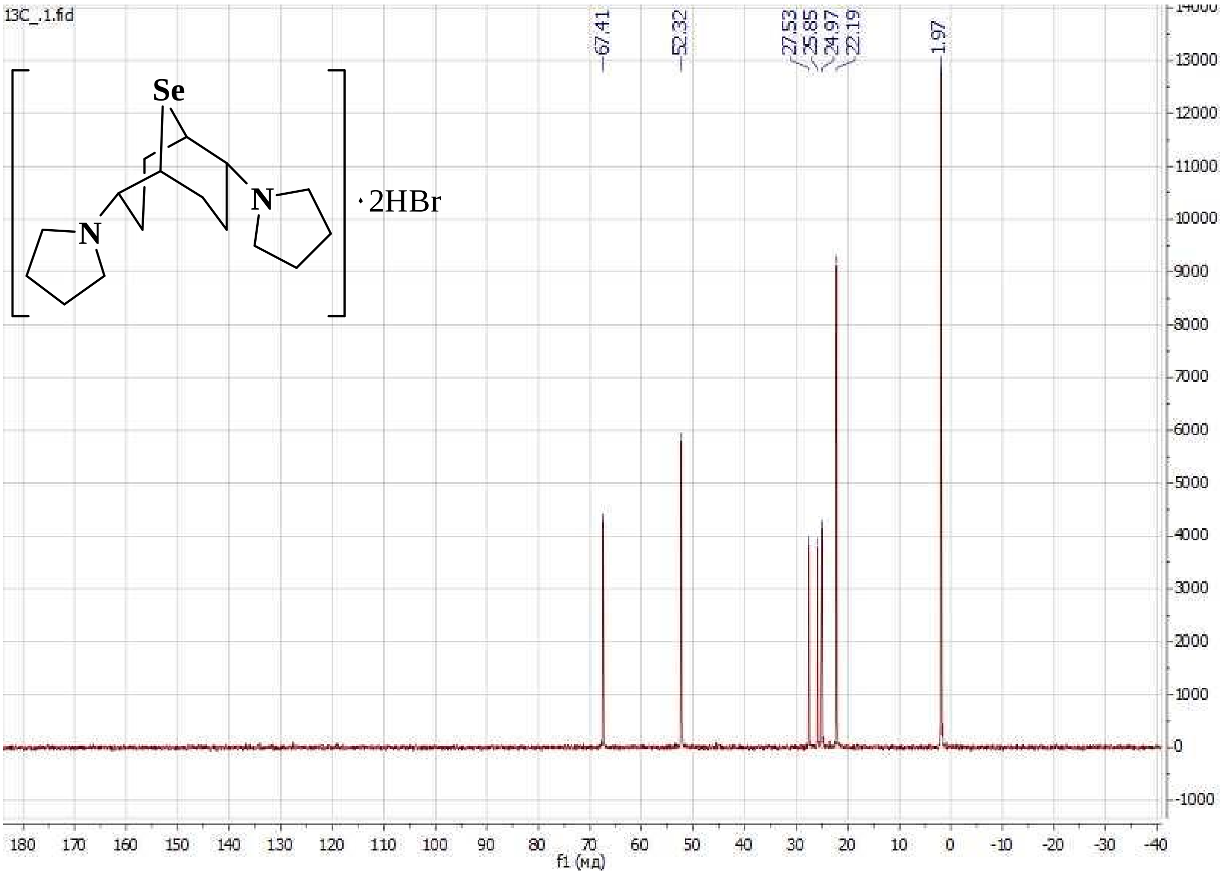

^{1}H- and ^{13}C-NMR spectra of compound 7 (D_2O)

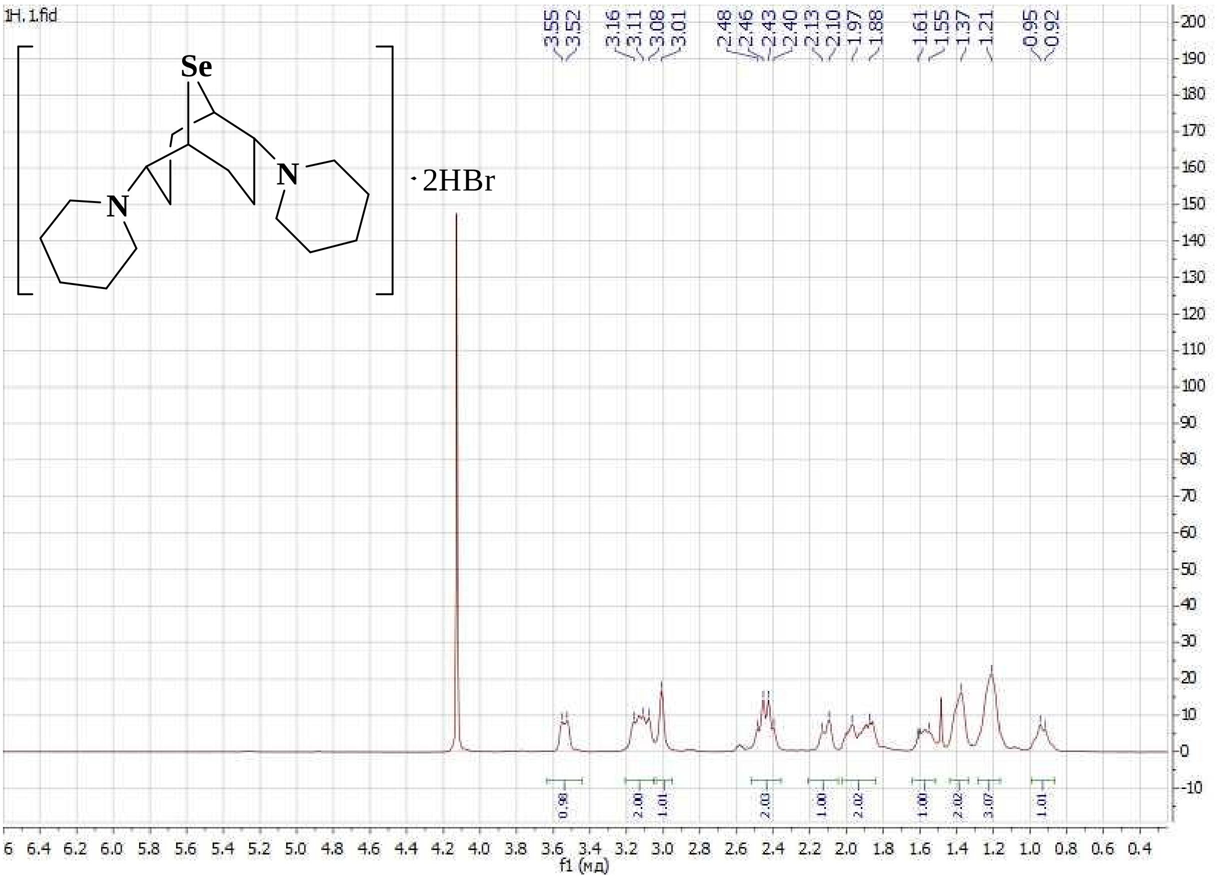

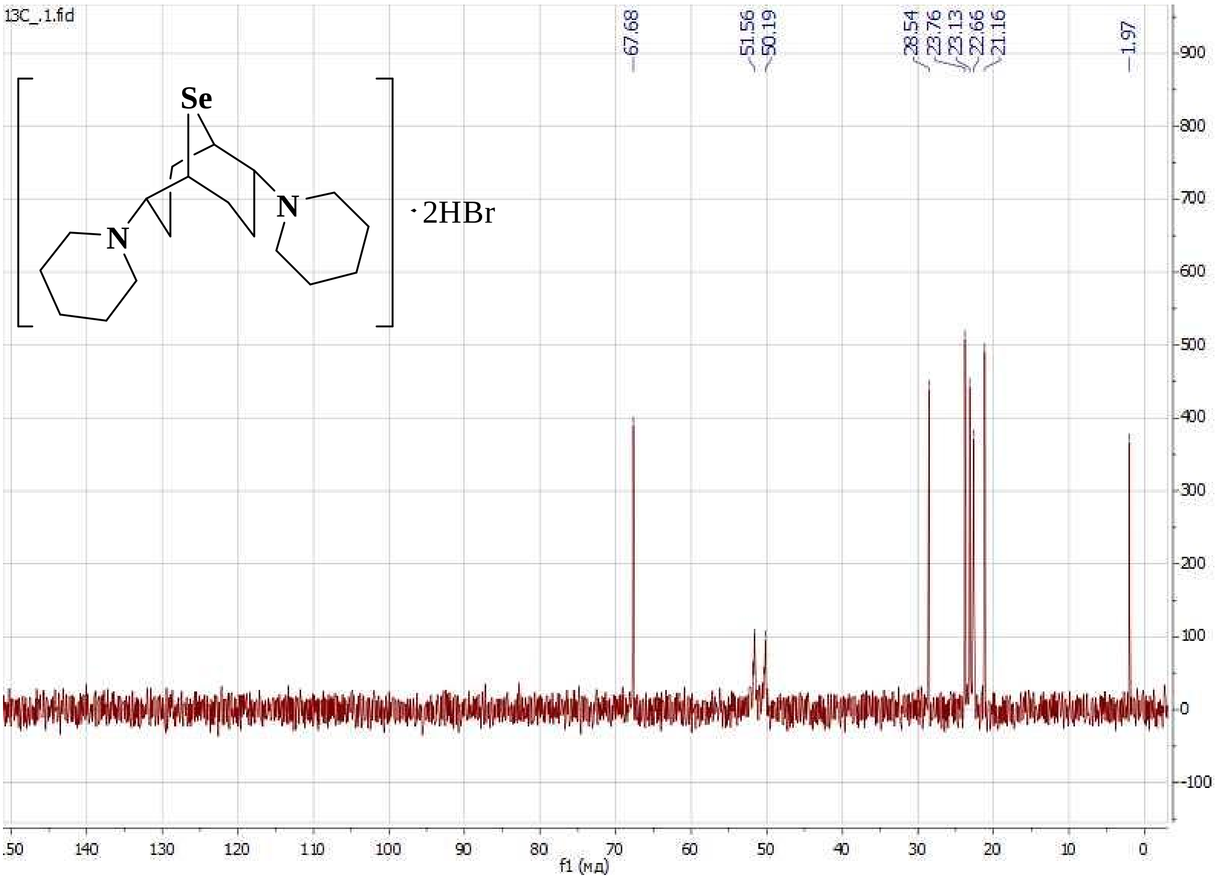

^{1}H-NMR spectrum of compound 8 (DMSO-d_6)

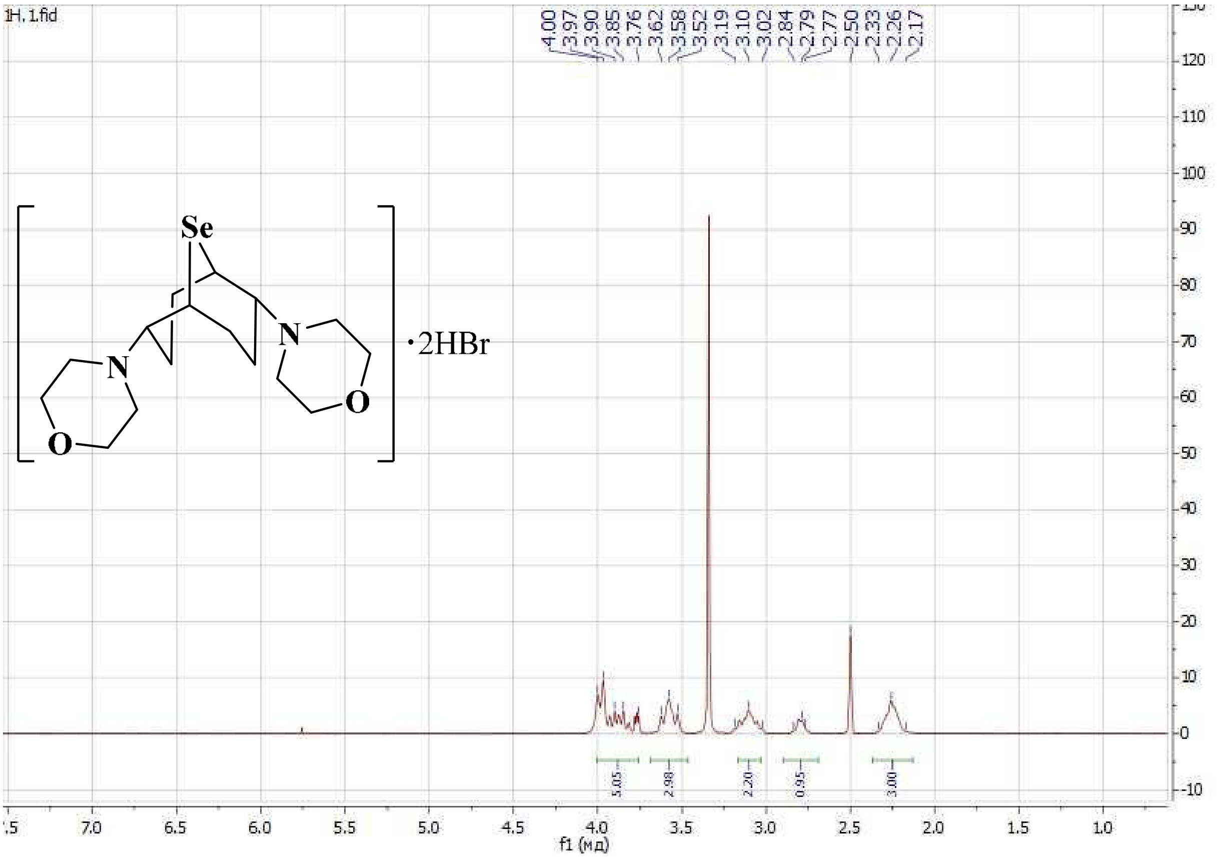

^{13}C-NMR spectrum of compound 8 (D_2O)

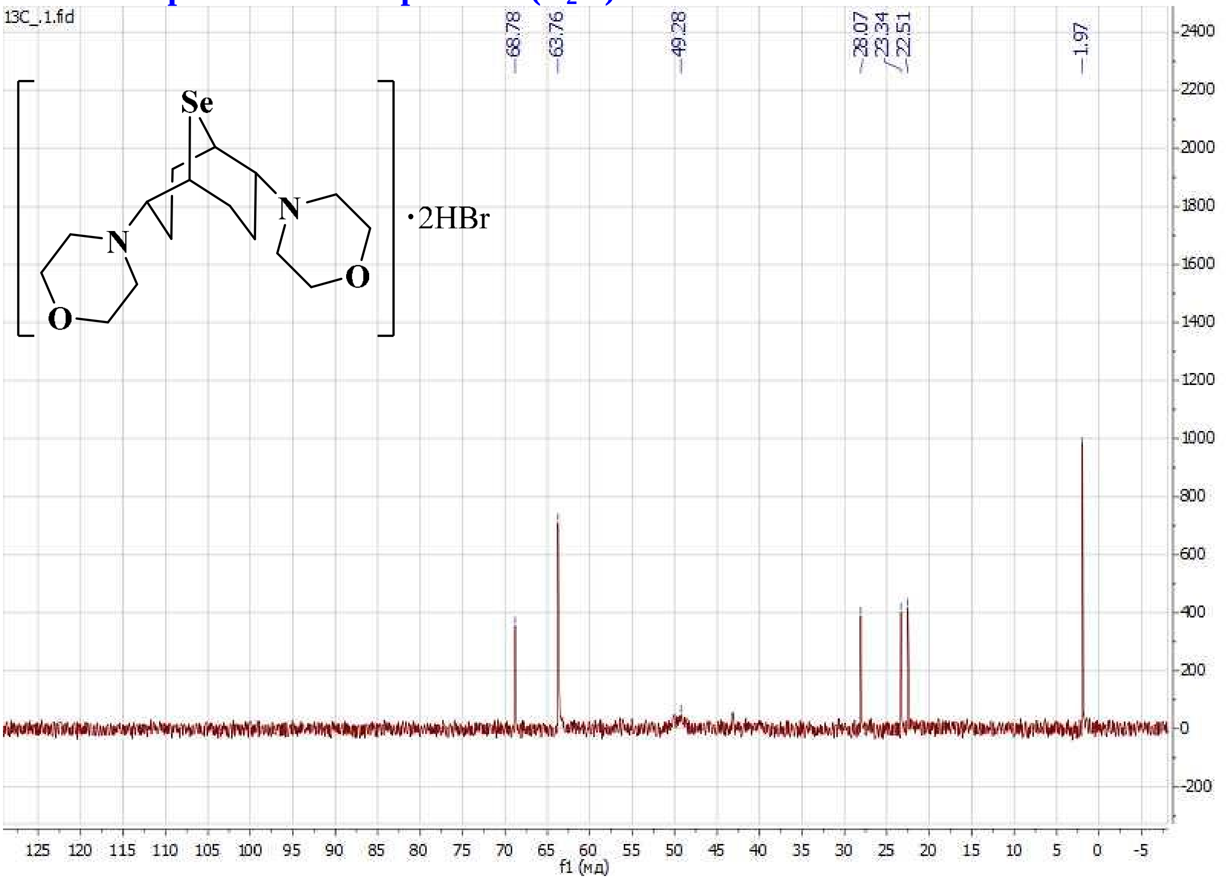

^{1}H- and ^{13}C-NMR spectra of compound 9 (D_2O)

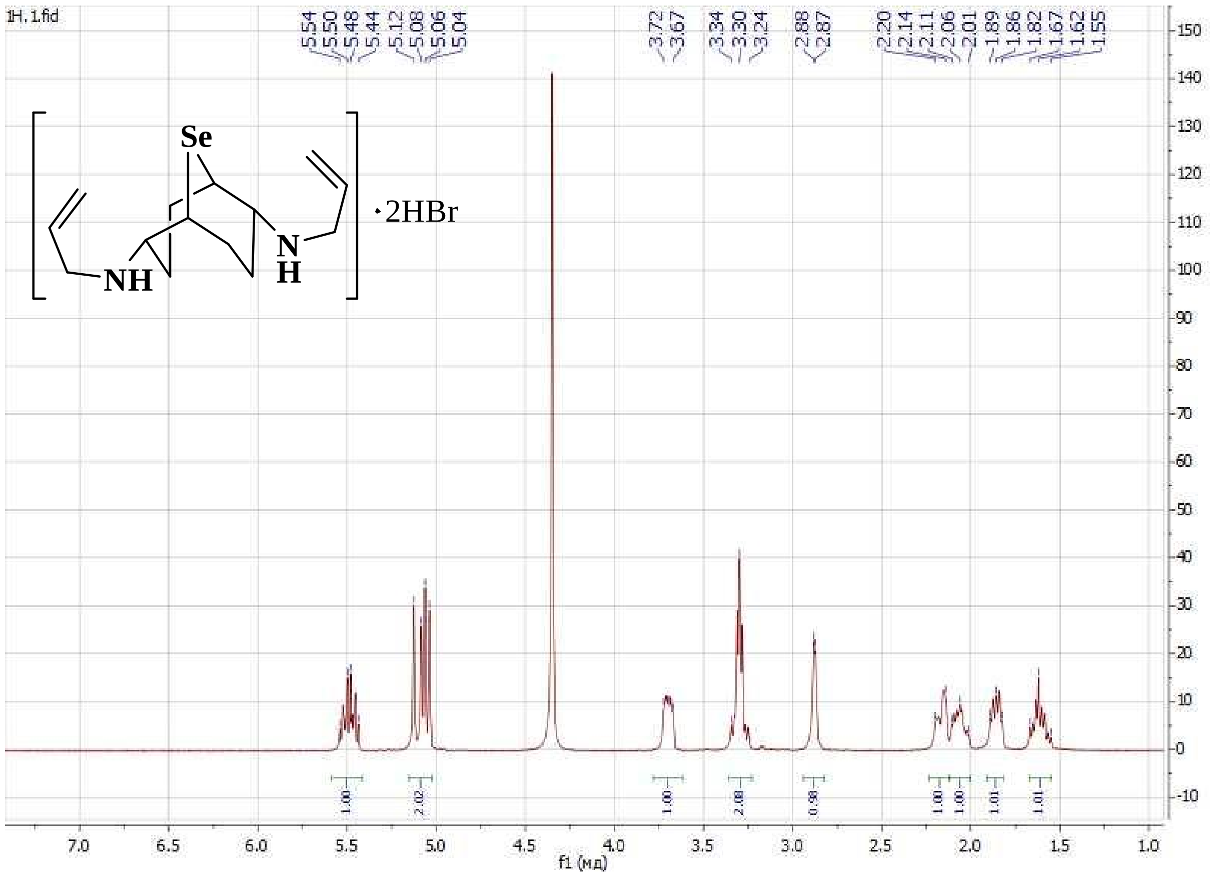

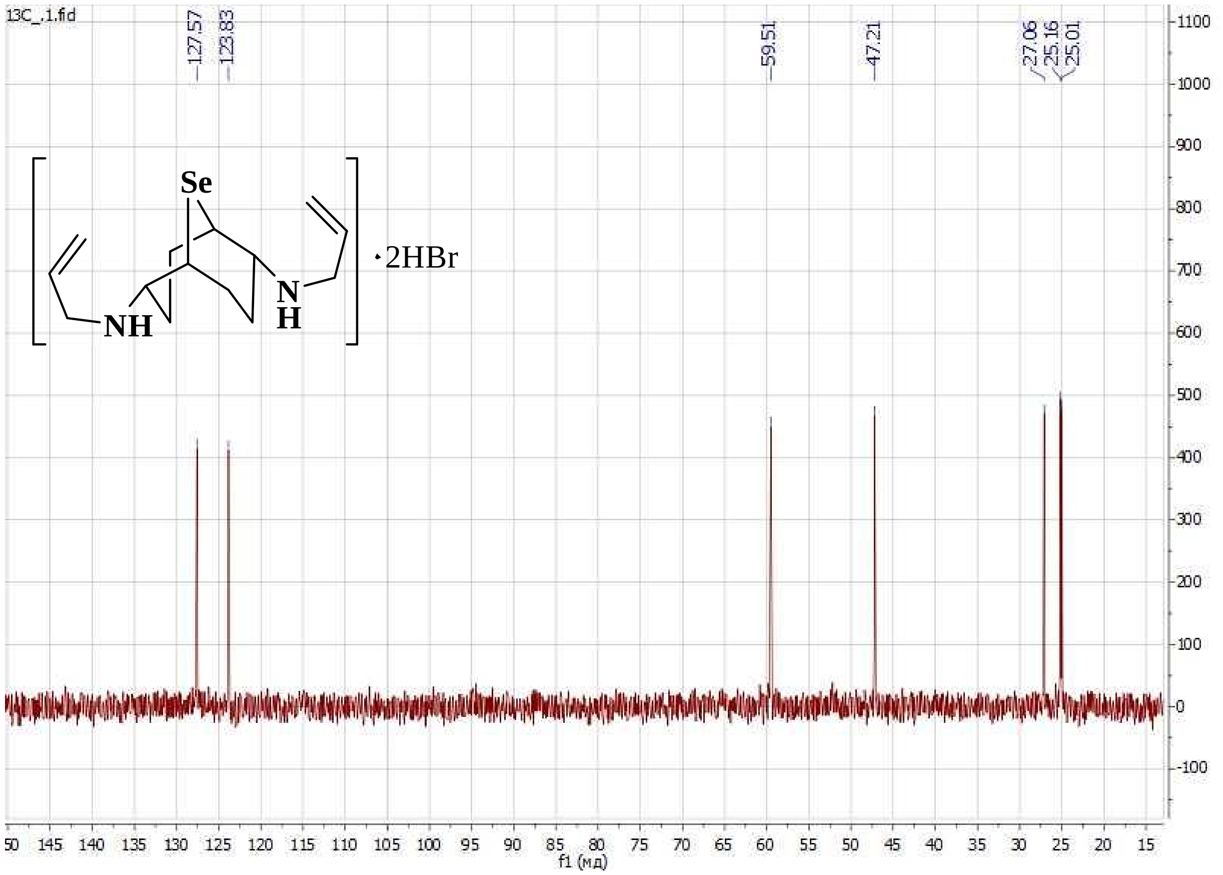

^{1}H- and ^{13}C-NMR spectra of compound 10 (D_2O)

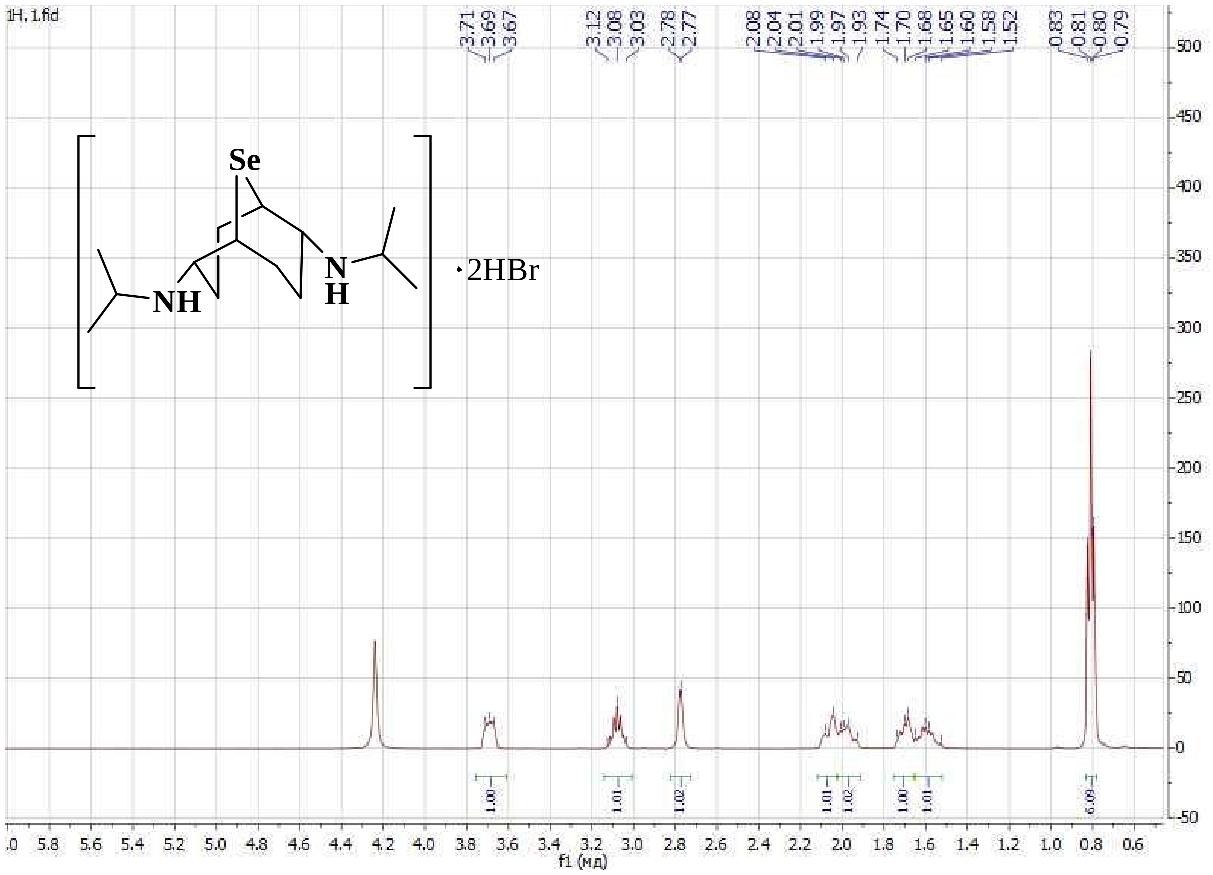

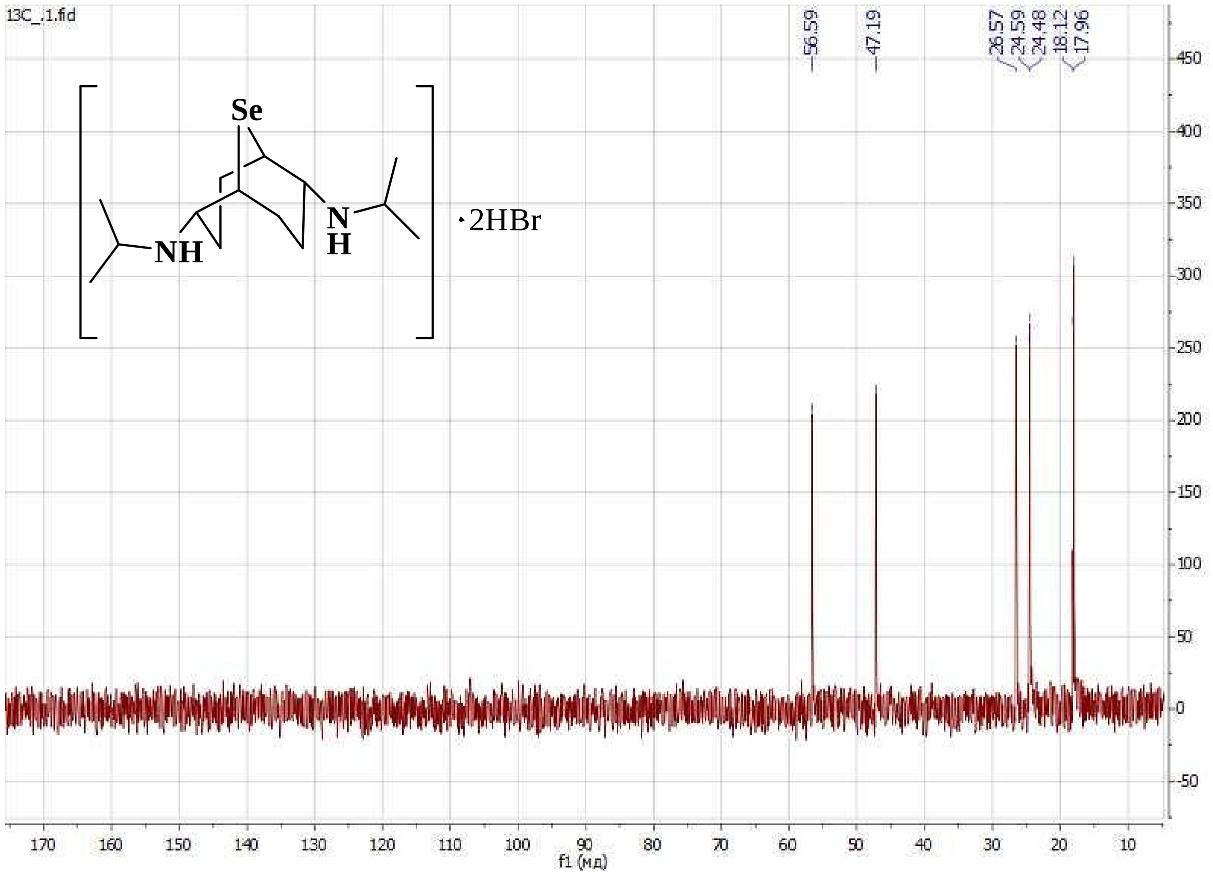

^{1}H- and ^{13}C-NMR spectra of compound 11 (D_2O)

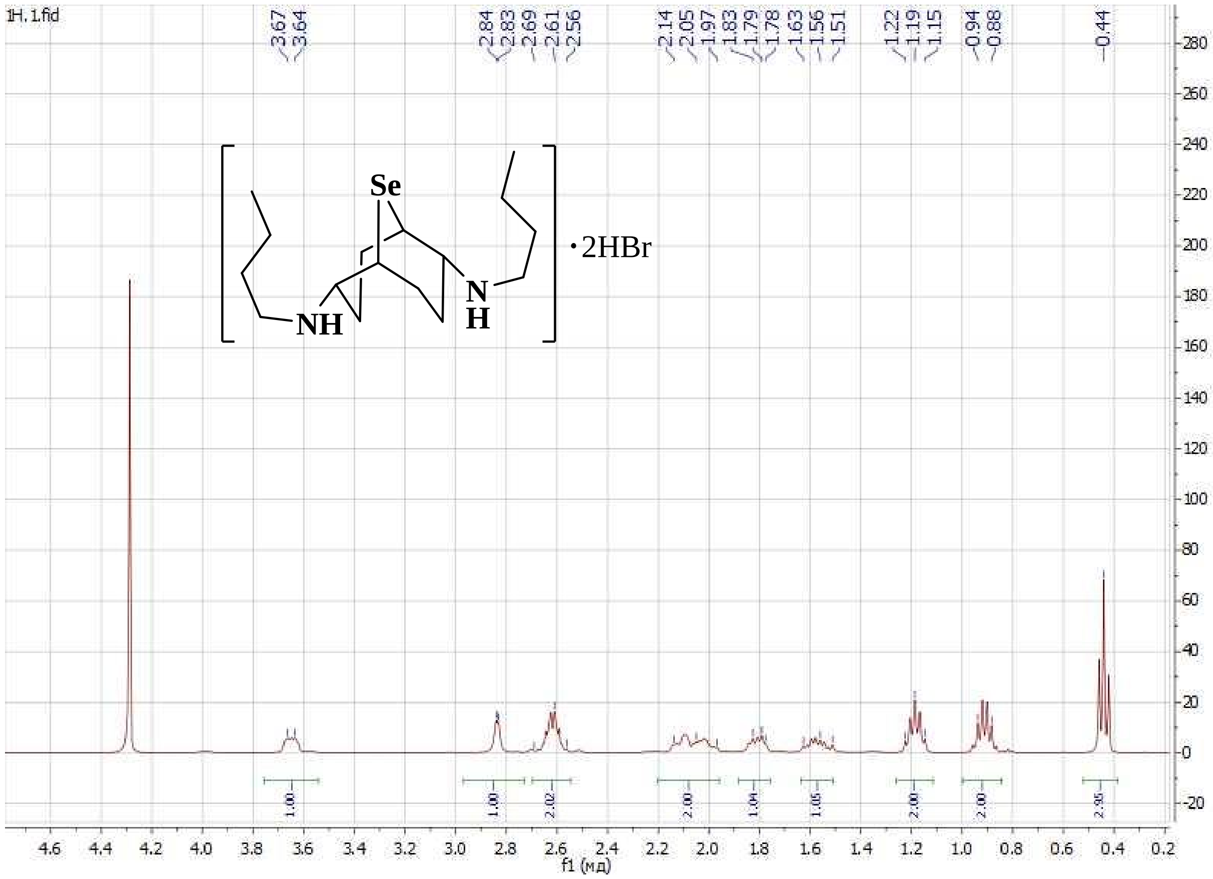

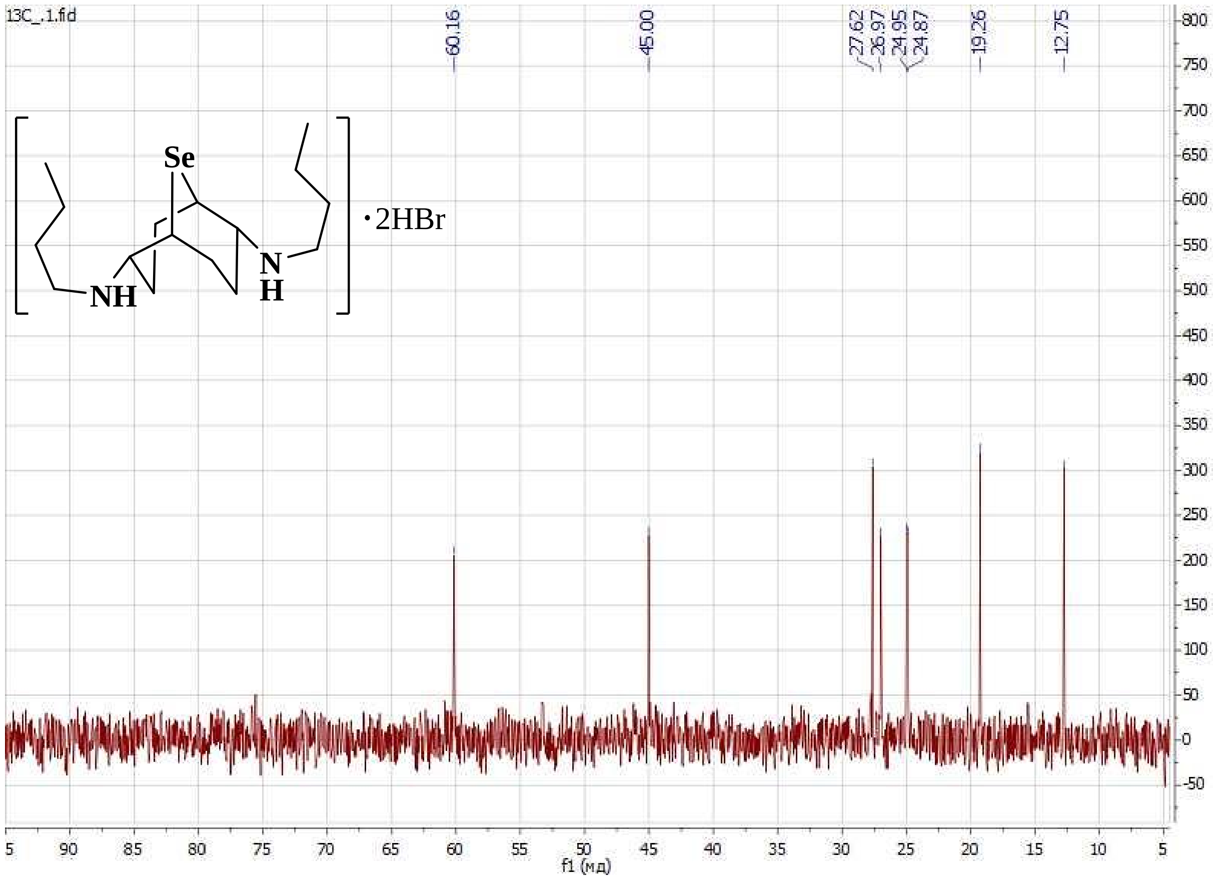

^{1}H- and ^{13}C-NMR spectra of compound 12 (D_2O)

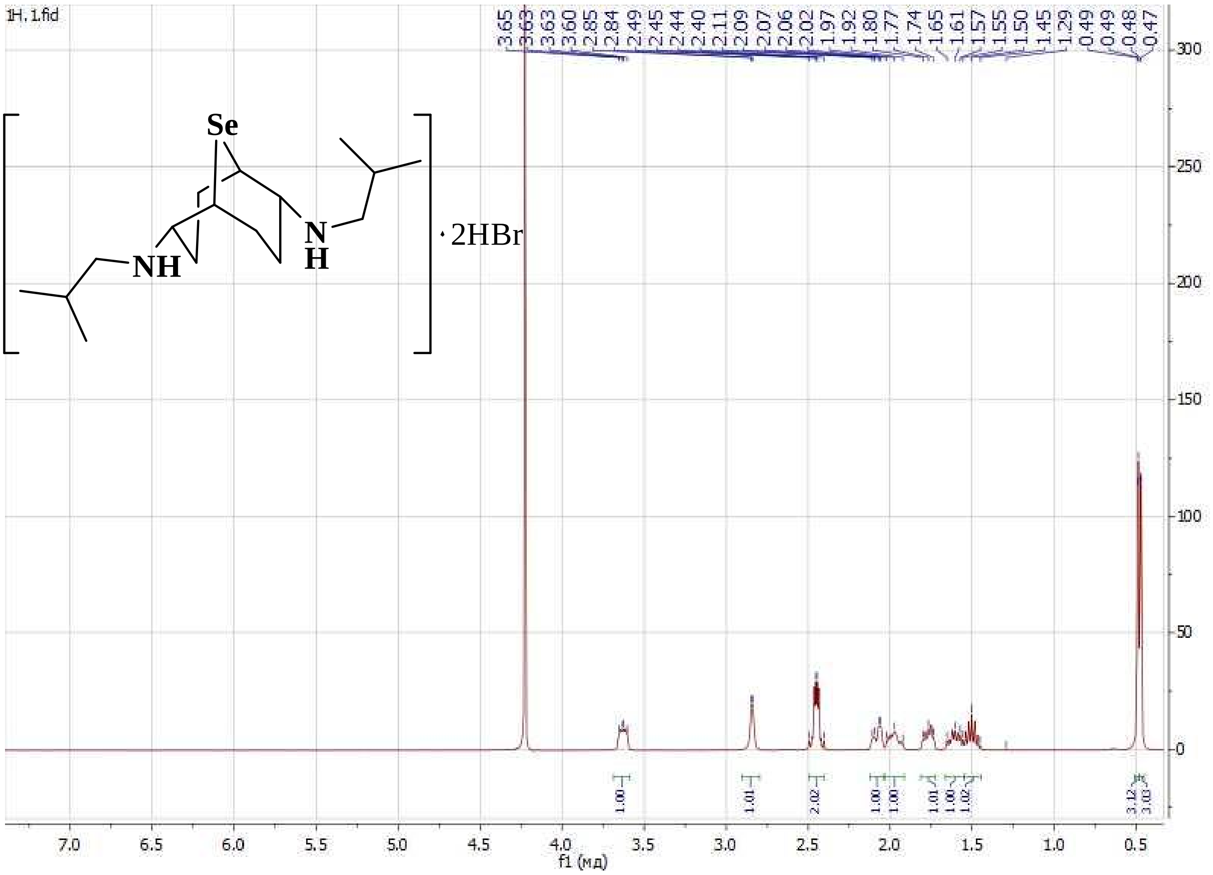

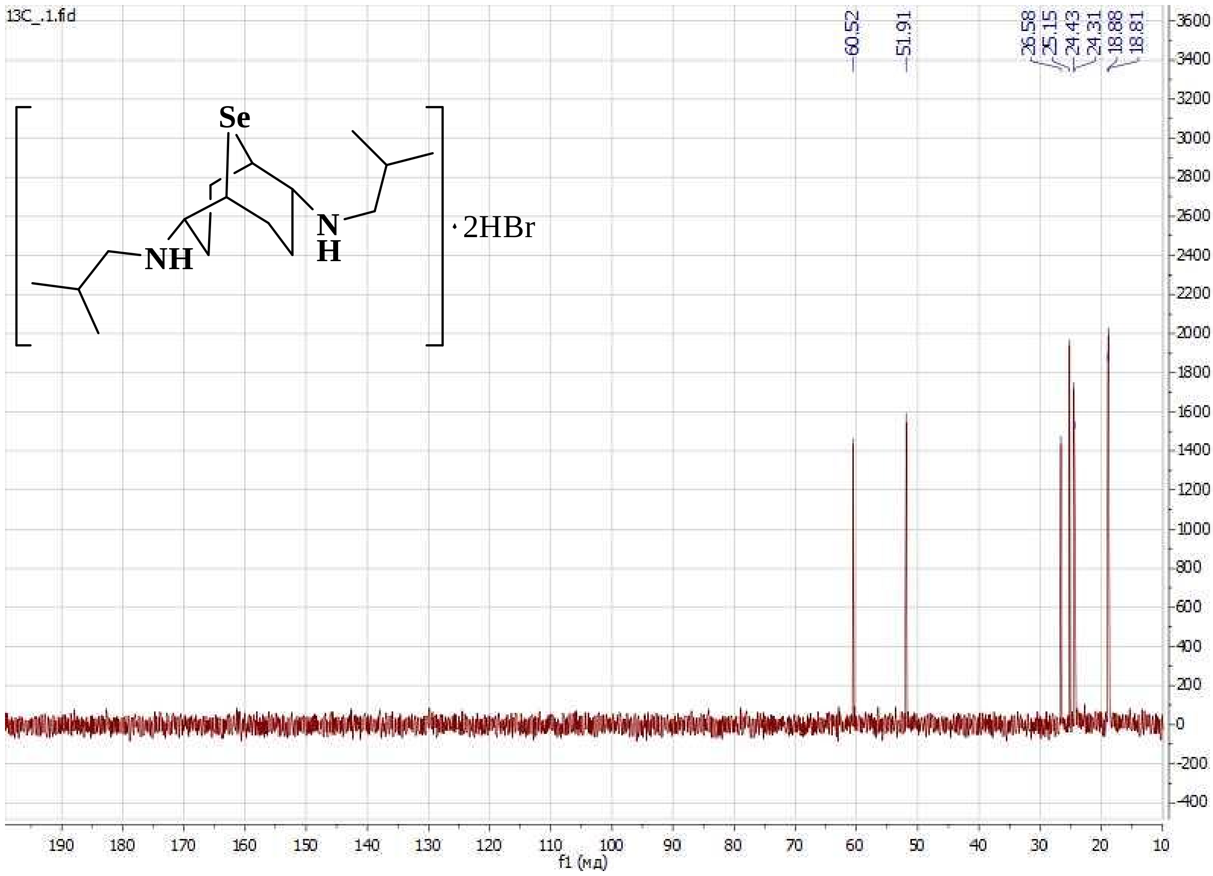

^{1}H- and ^{13}C-NMR spectra of compound 13 ($CDCl_3$)

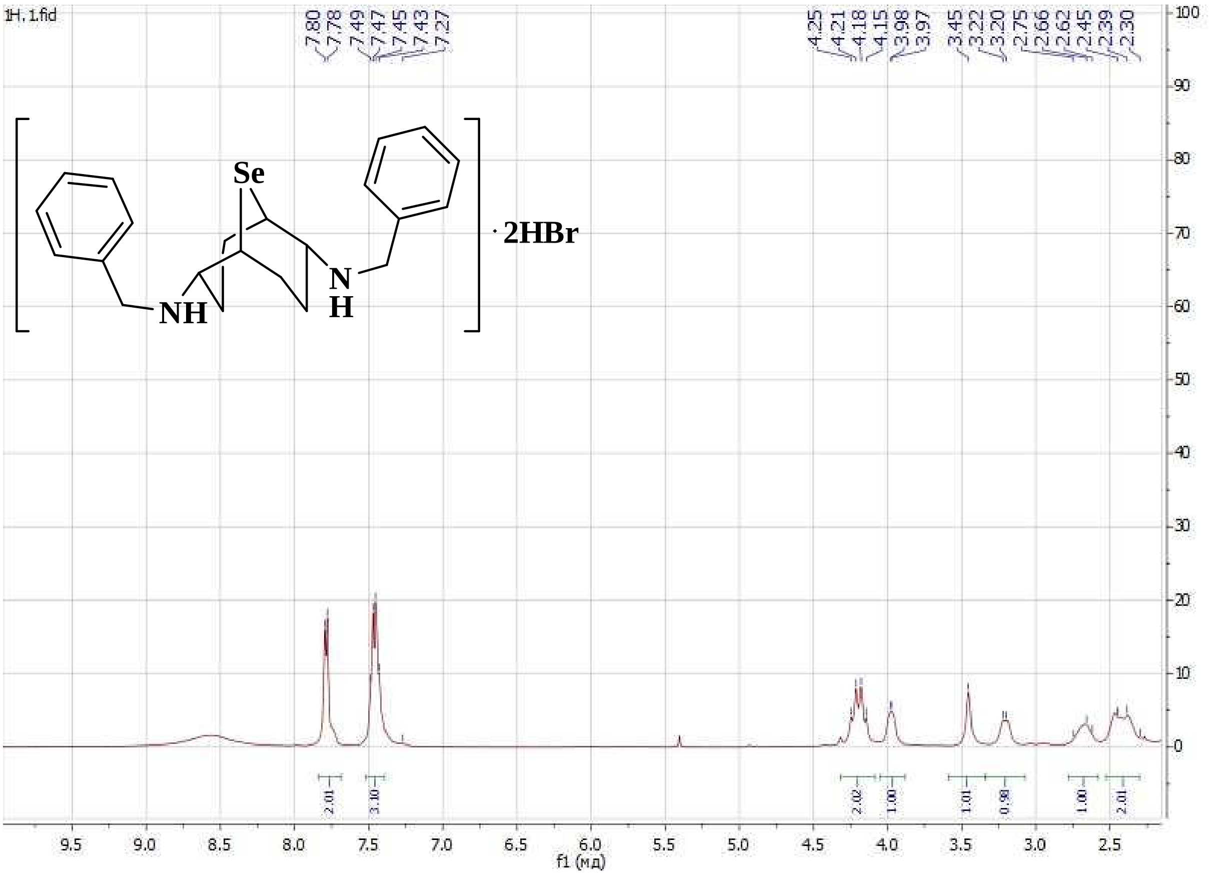

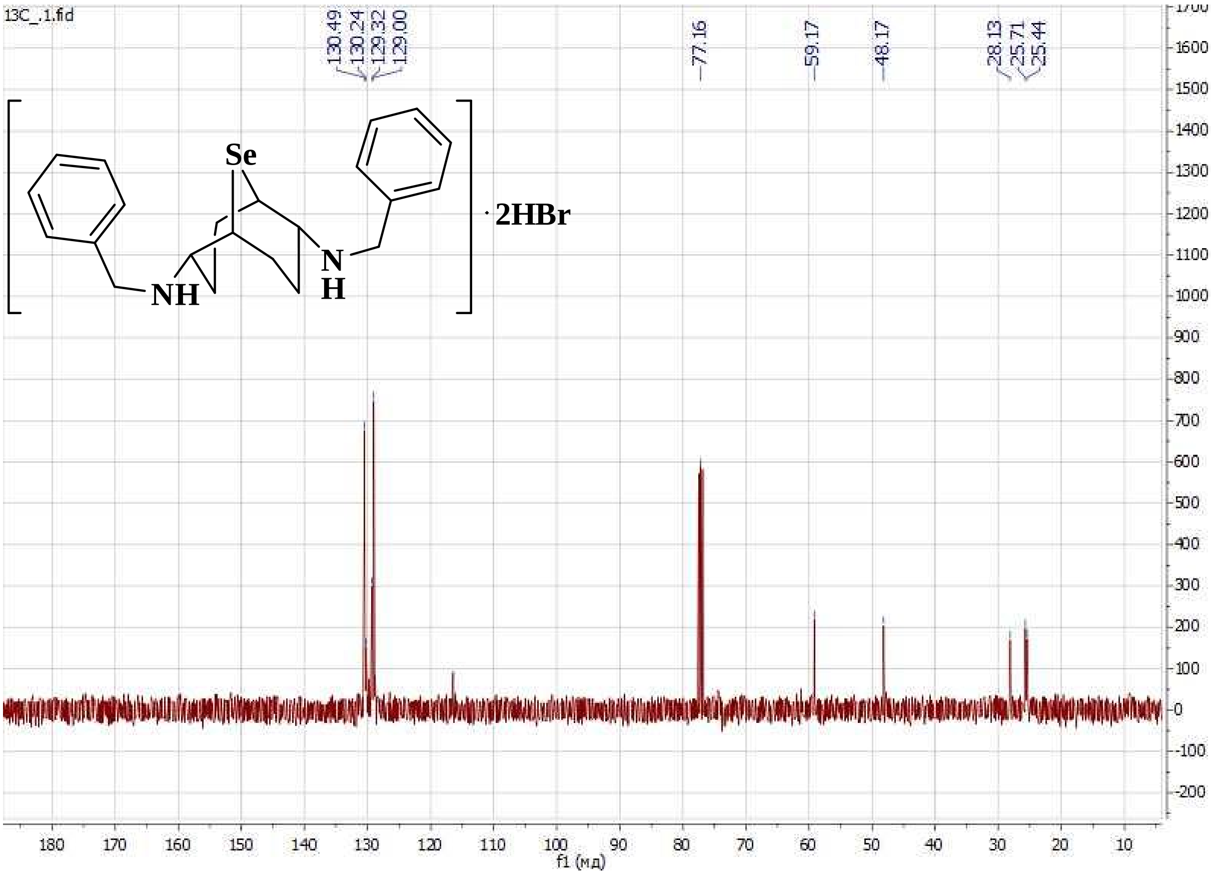

^{1}H-NMR spectrum of compound 14 ($CDCl_3$)

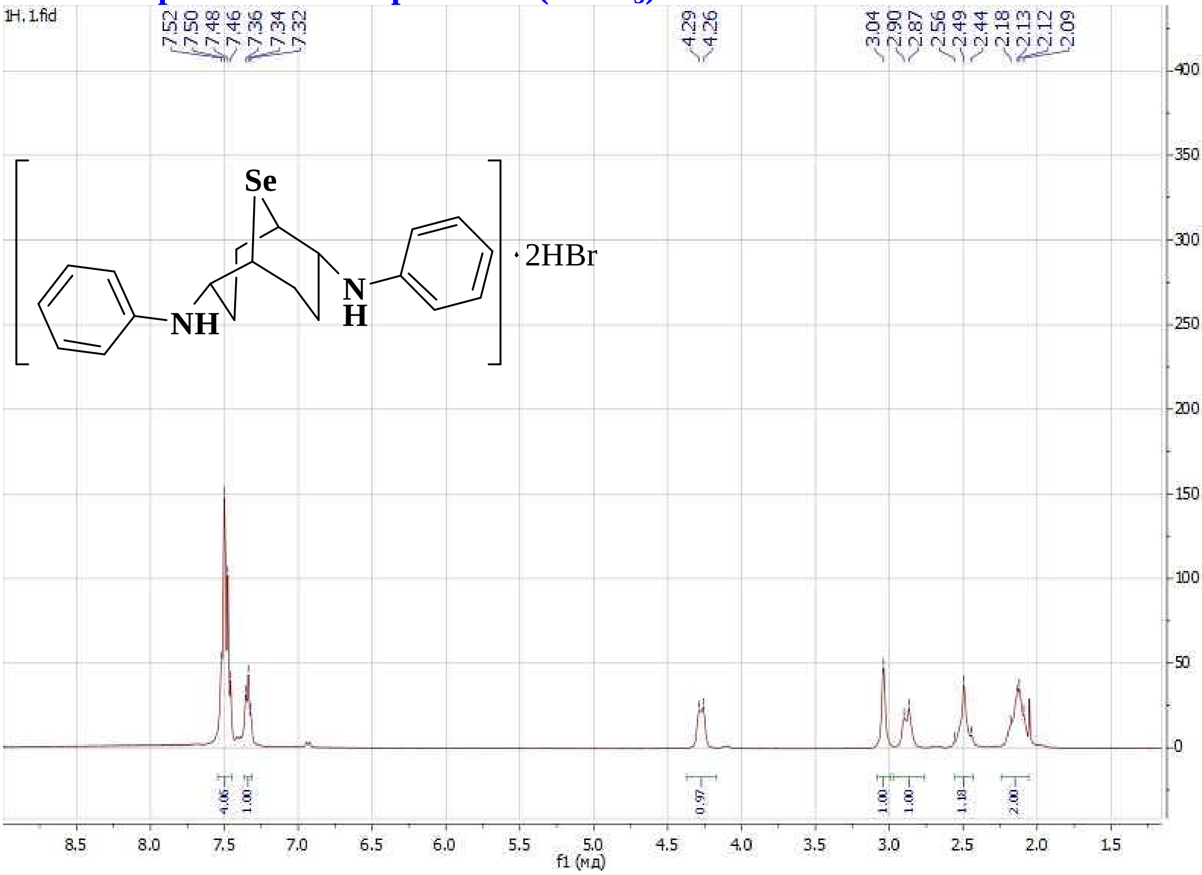

^{13}C-NMR spectrum of compound 14 (DMSO-d_6)

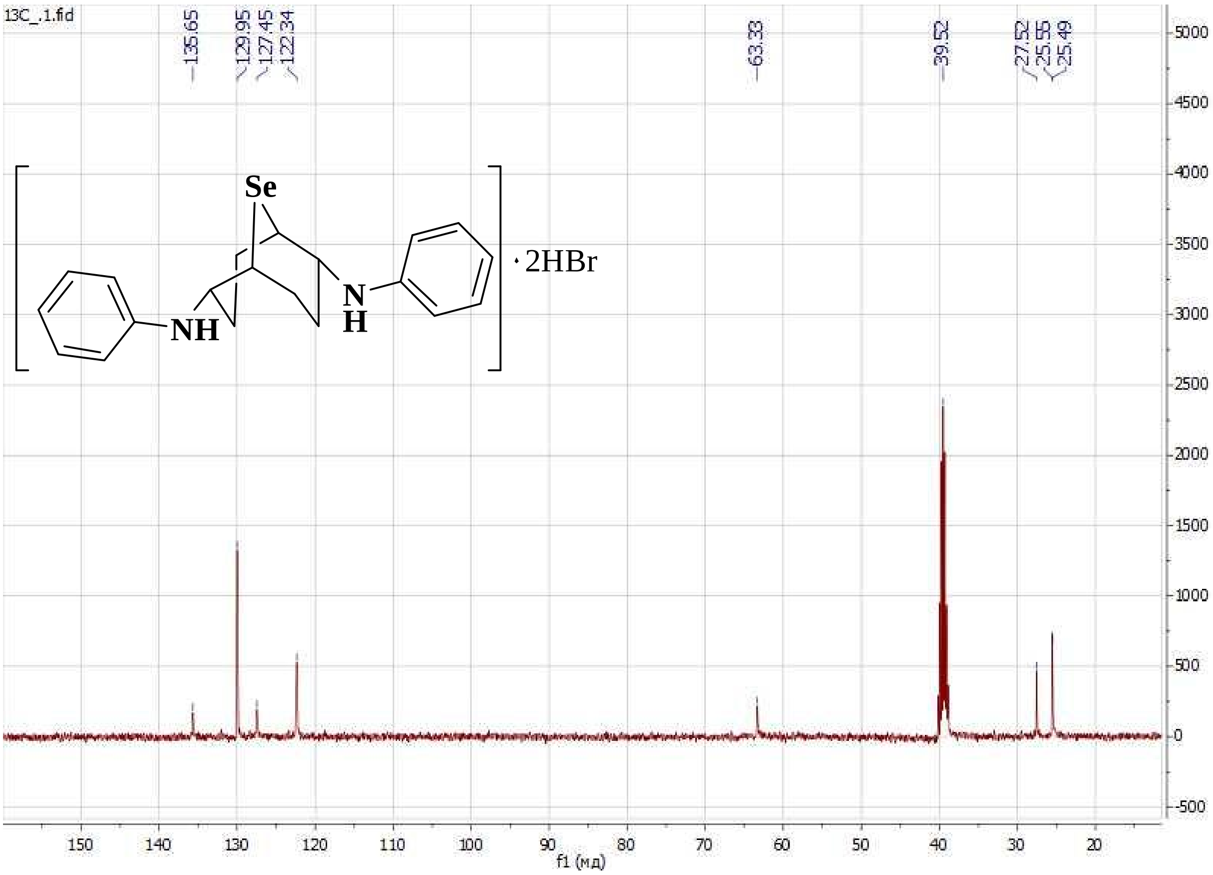

^{1}H- and ^{13}C-NMR spectra of compound 15 (D_2O)

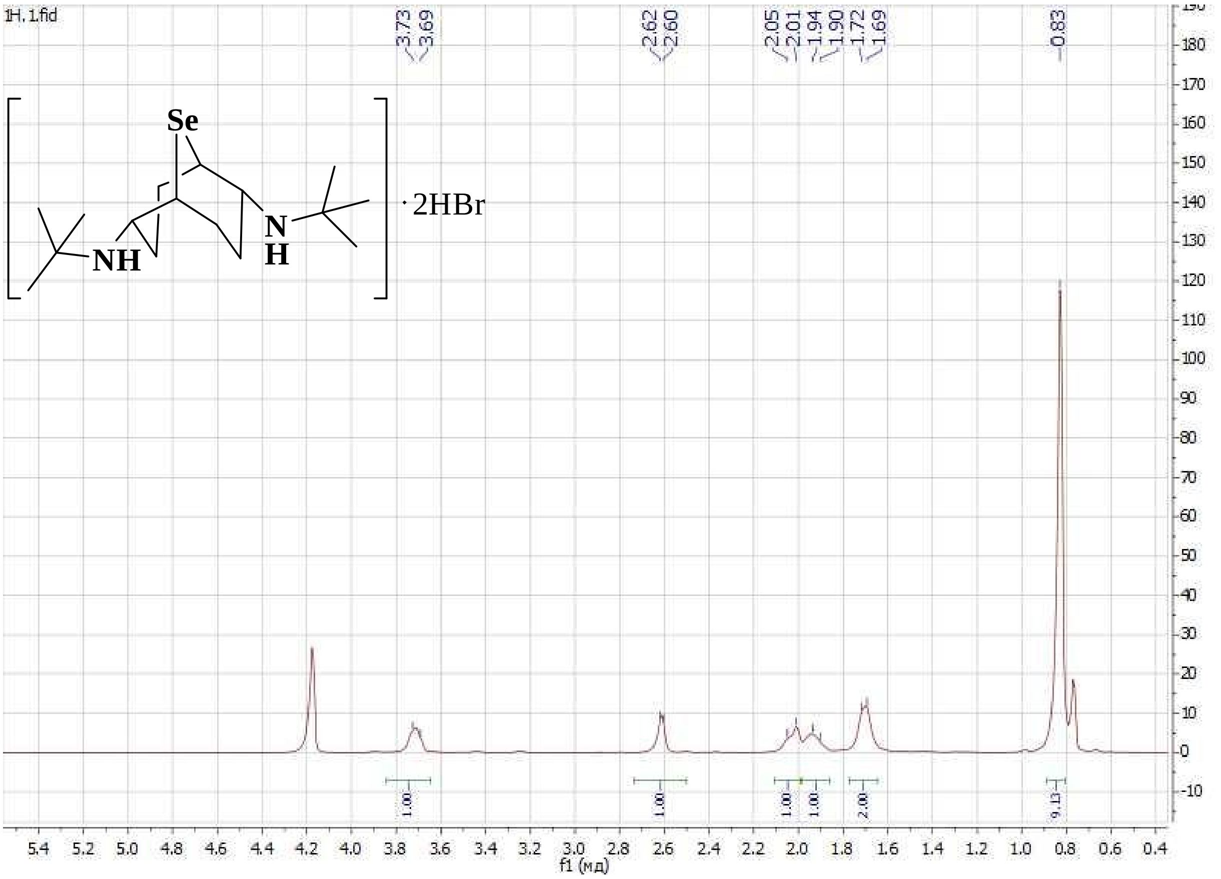

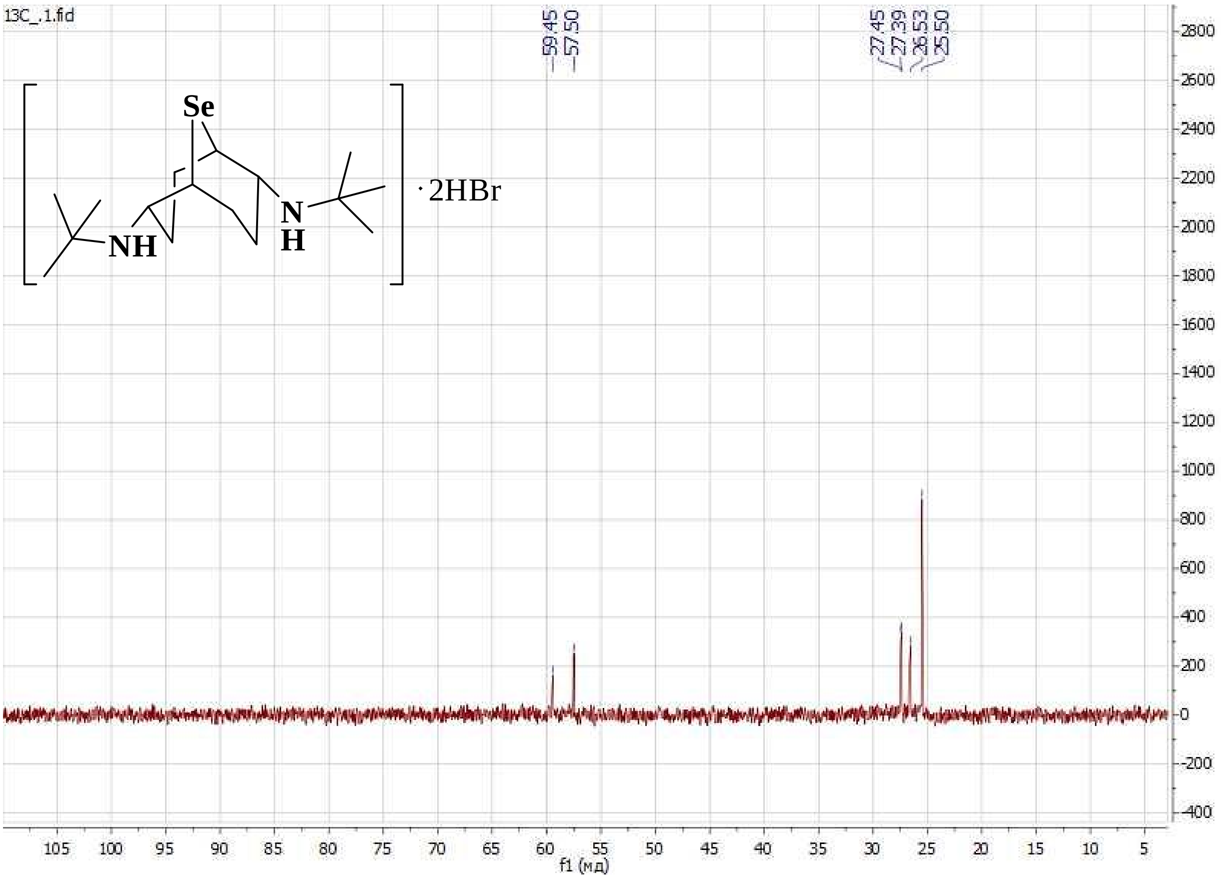

^{1}H- and ^{13}C-NMR spectra of compound 16 (D_2O)

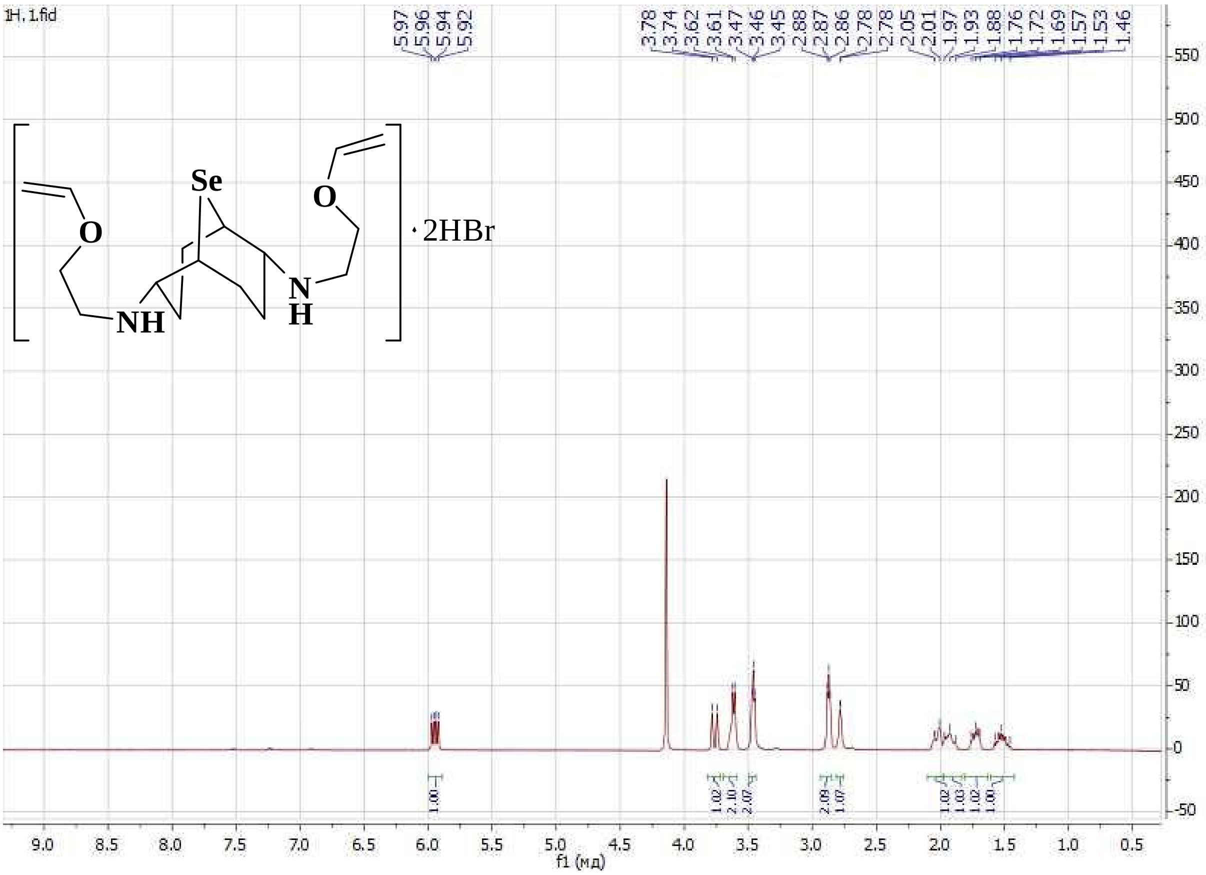

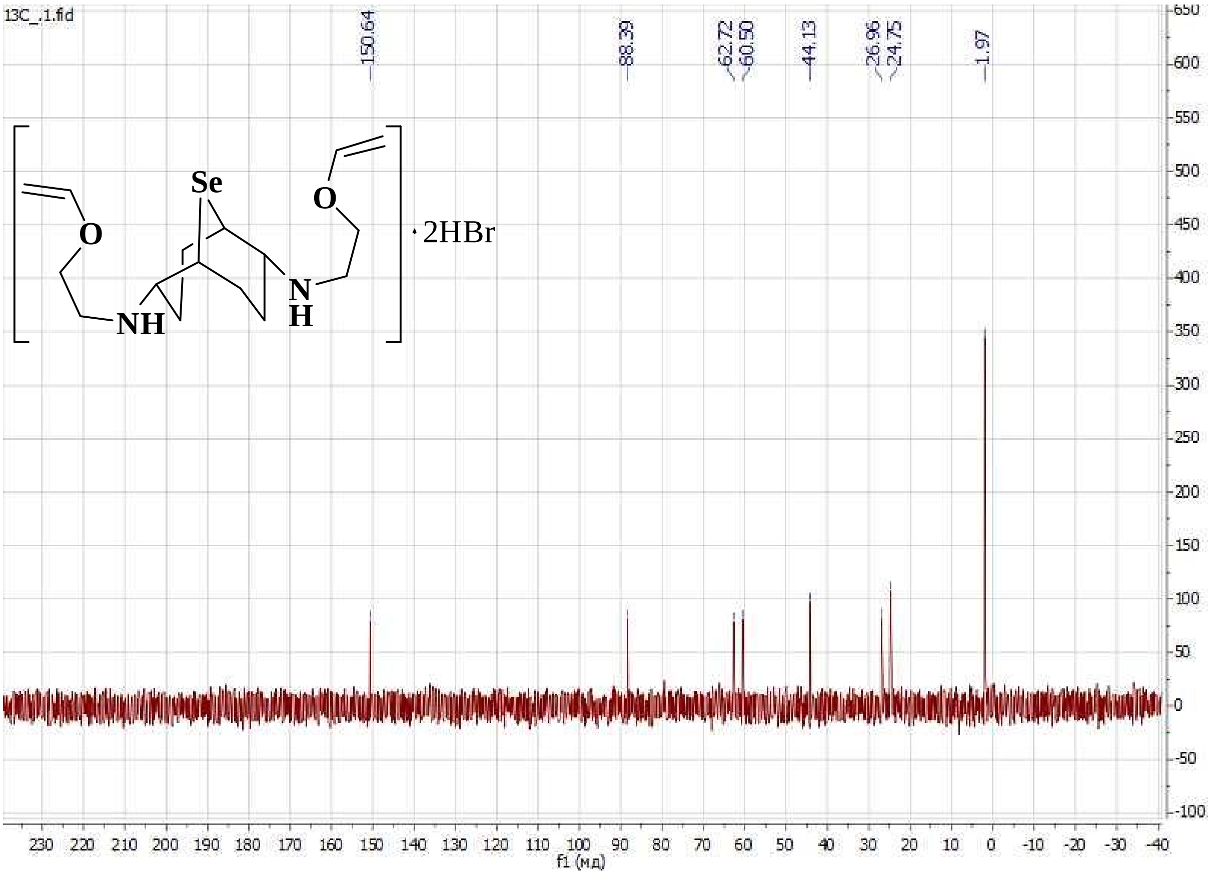

^{1}H- and ^{13}C-NMR spectra of compound 17 ($CDCl_3$)

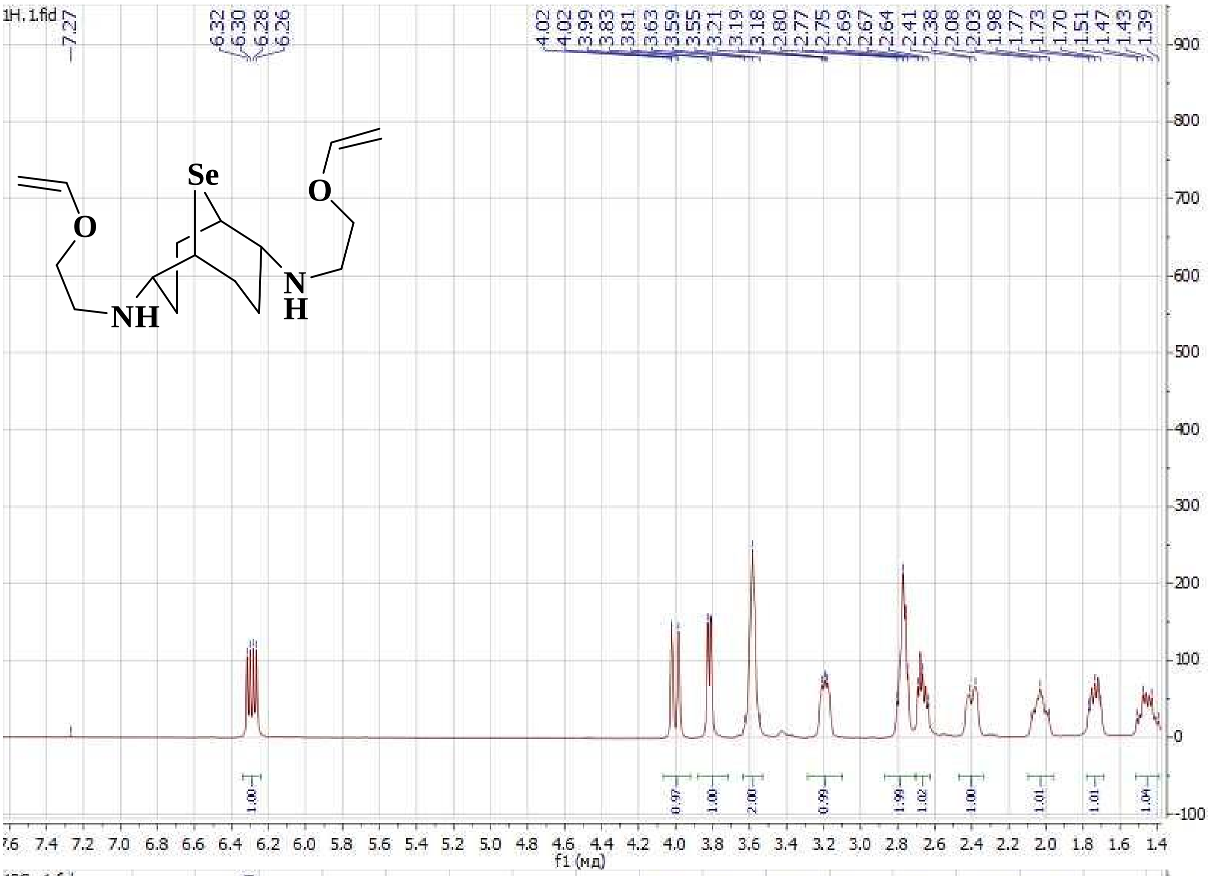

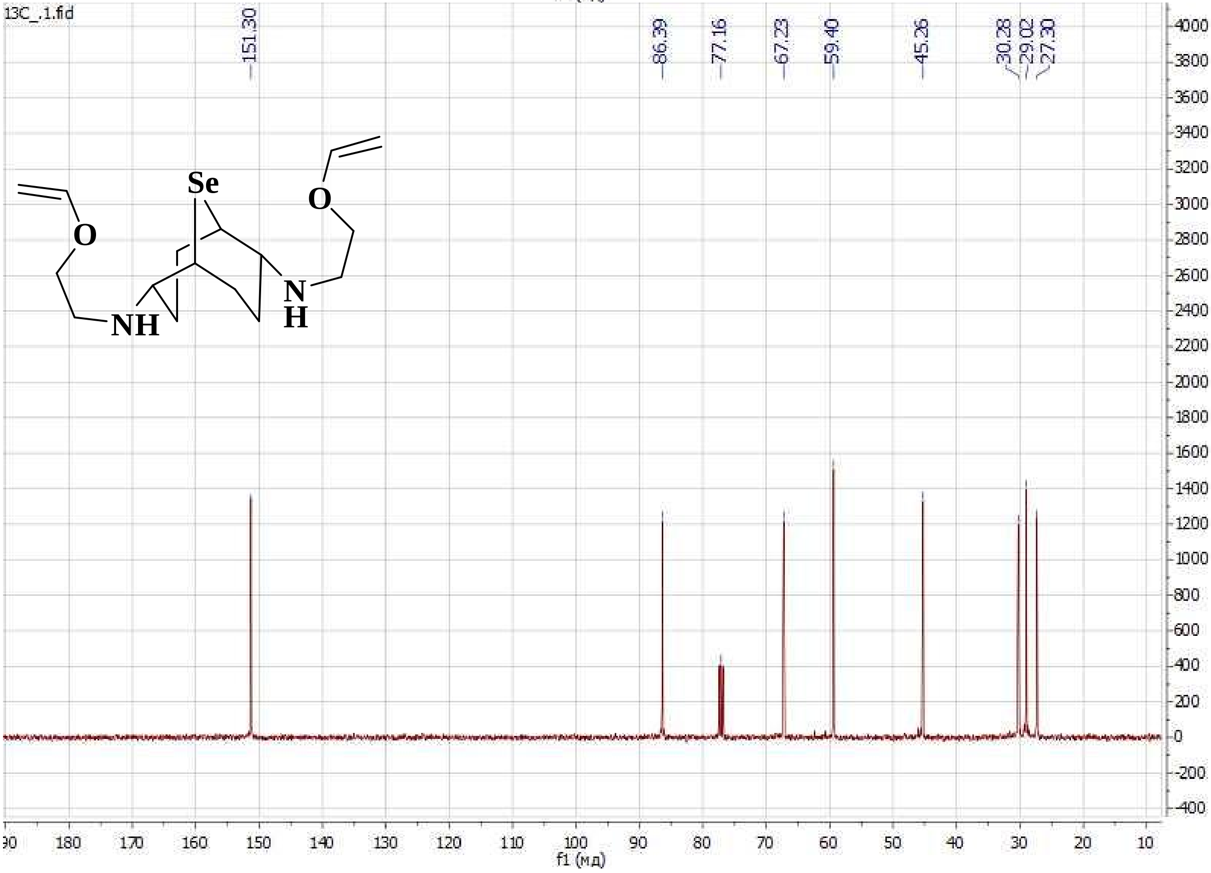

^{1}H- and ^{13}C-NMR spectra of compound 18 ($CDCl_3$)

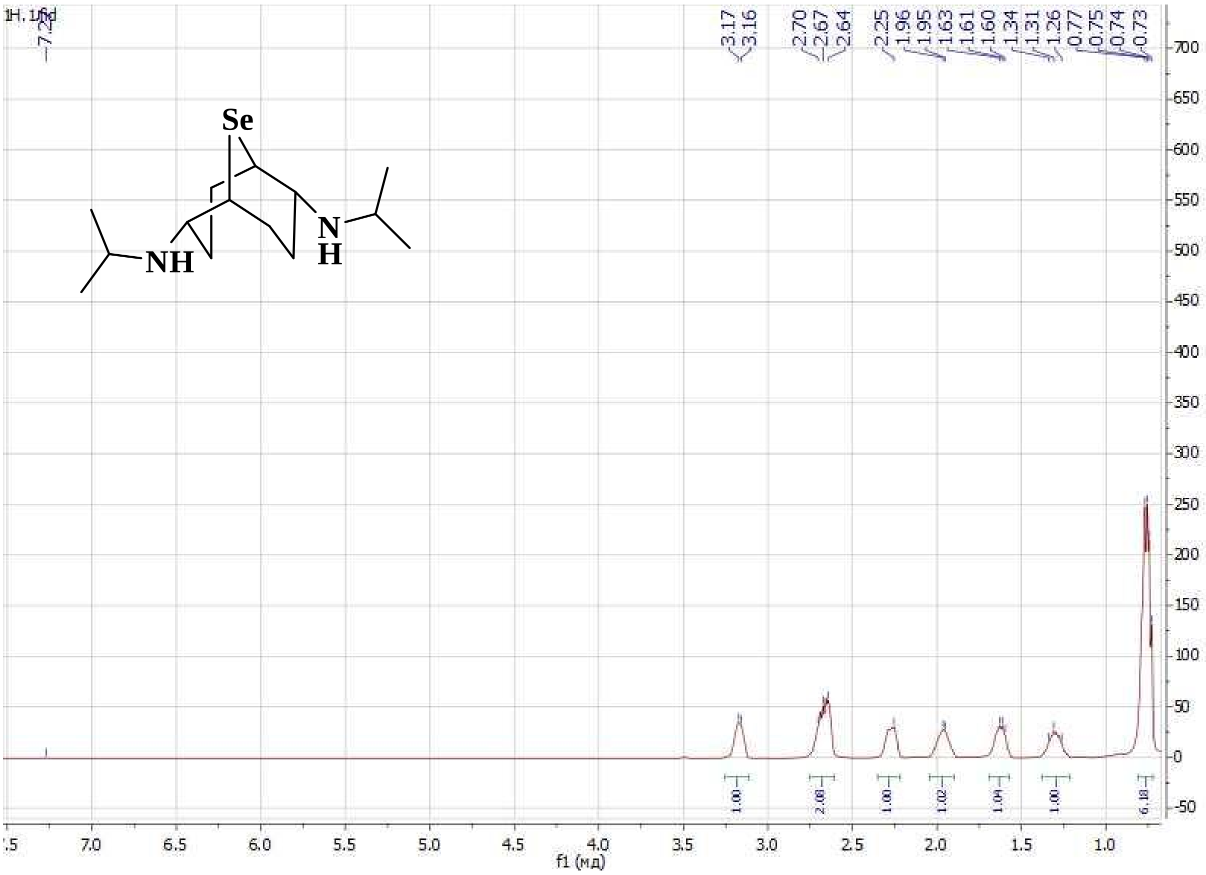

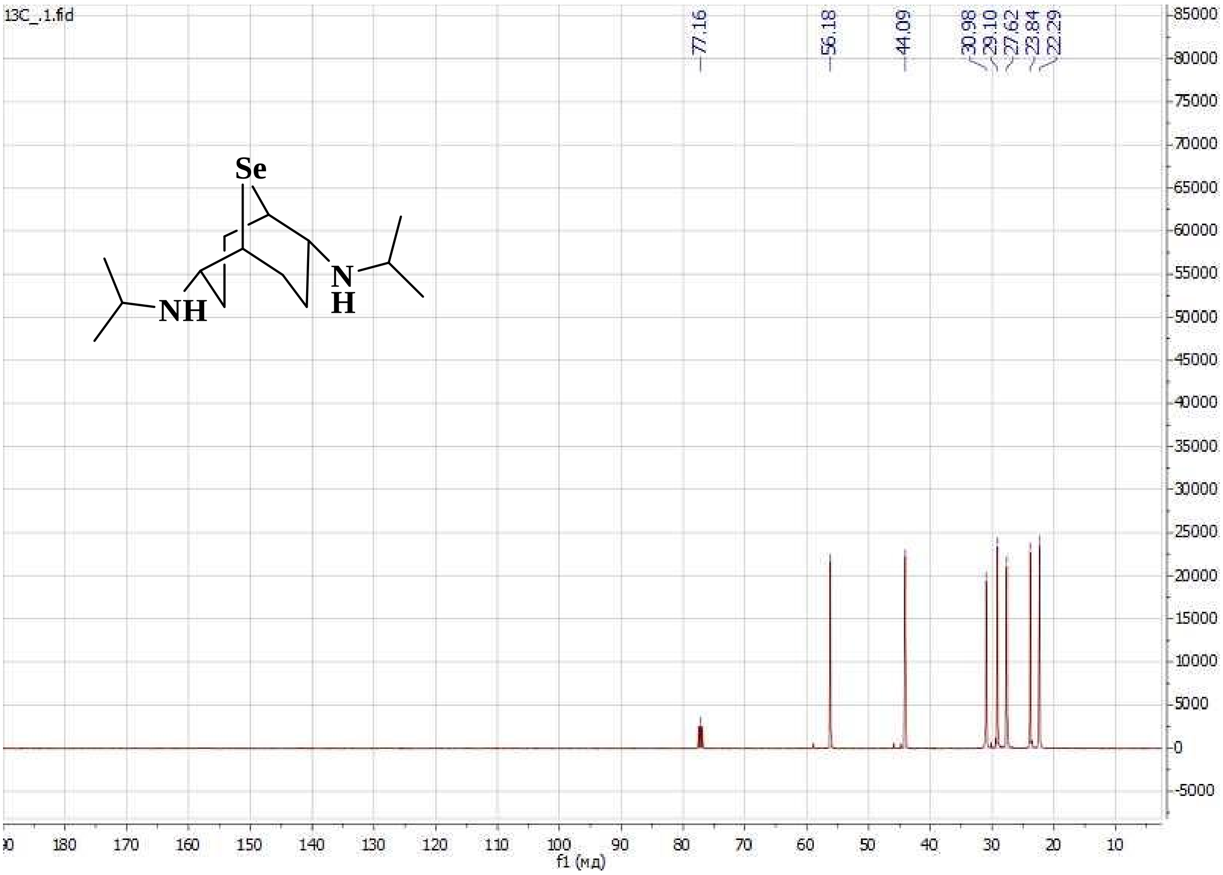

^{1}H- and ^{13}C-NMR spectra of compound 19 ($CDCl_3$)

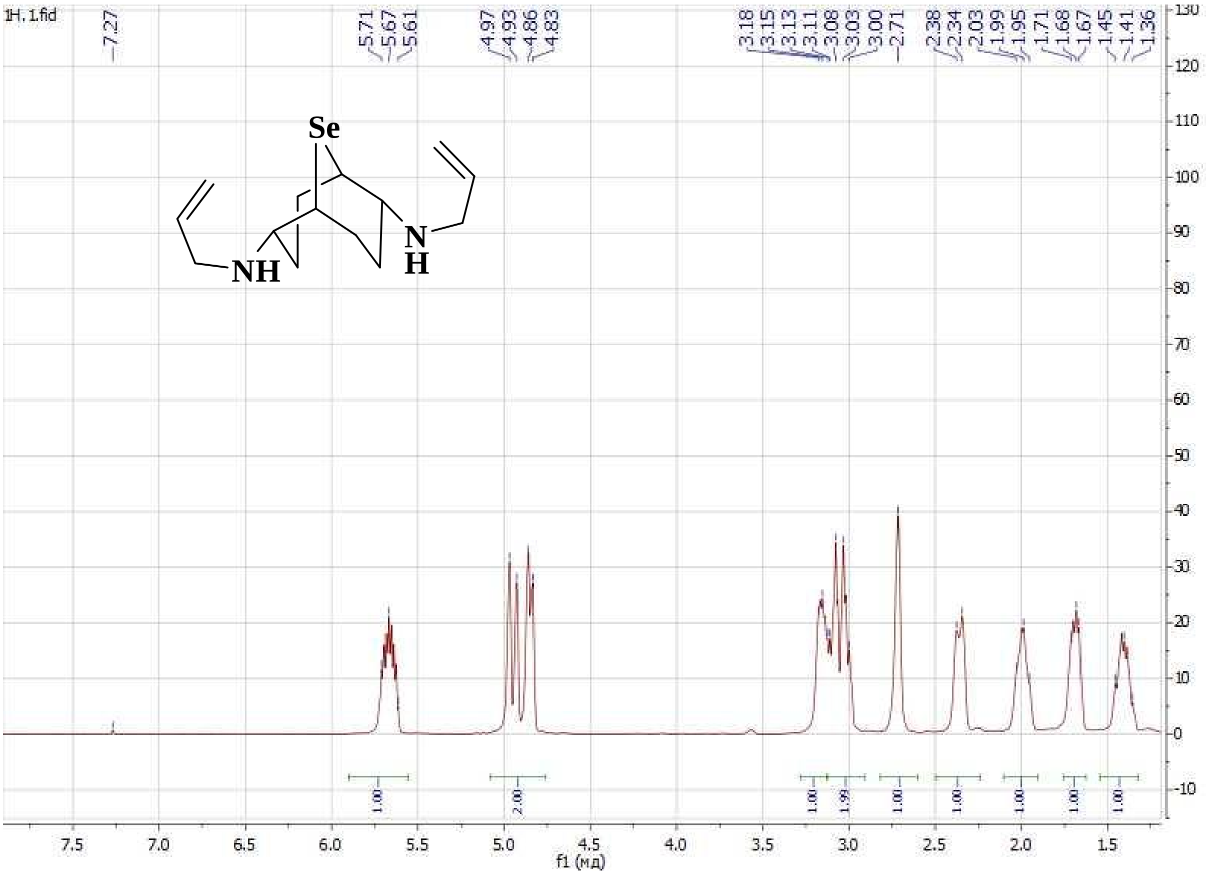

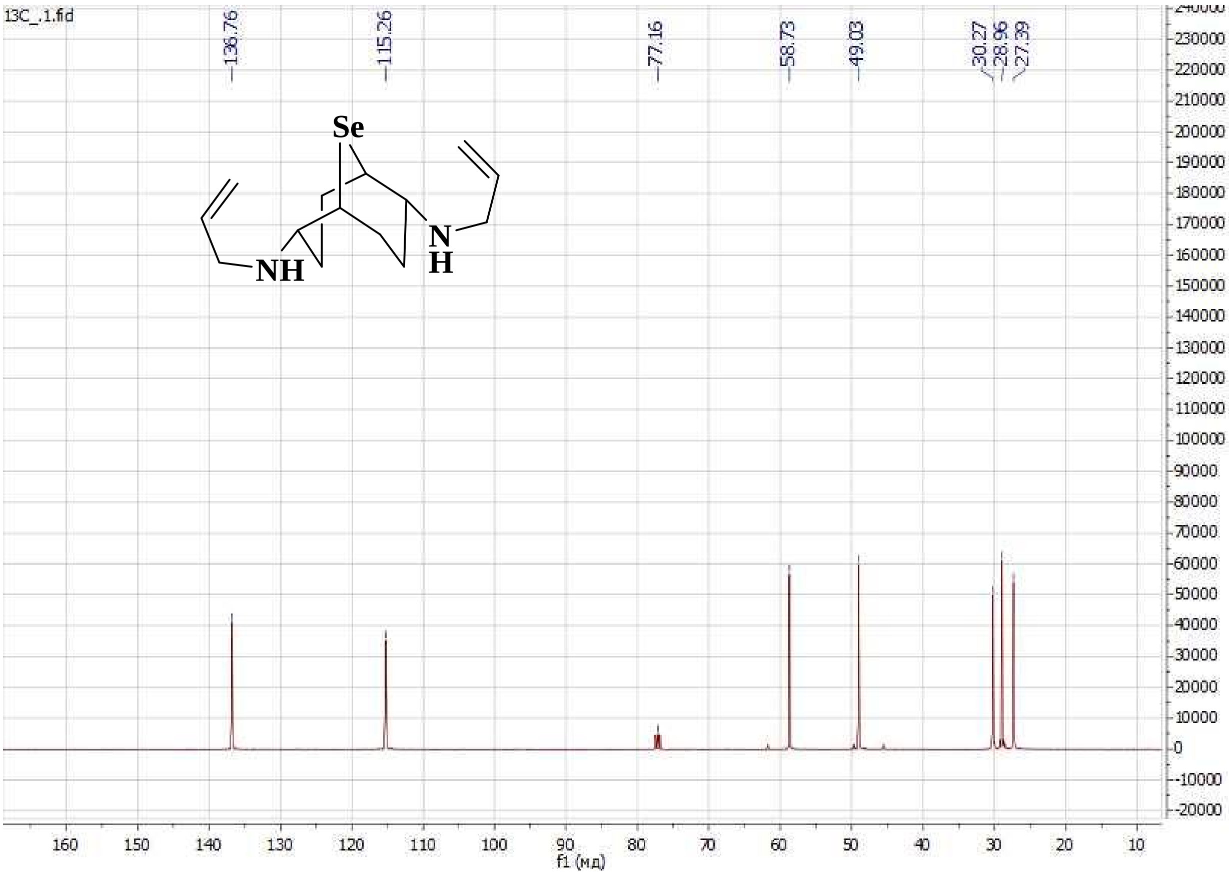

^{1}H- and ^{13}C-NMR spectra of compound 20 ($CDCl_3$)

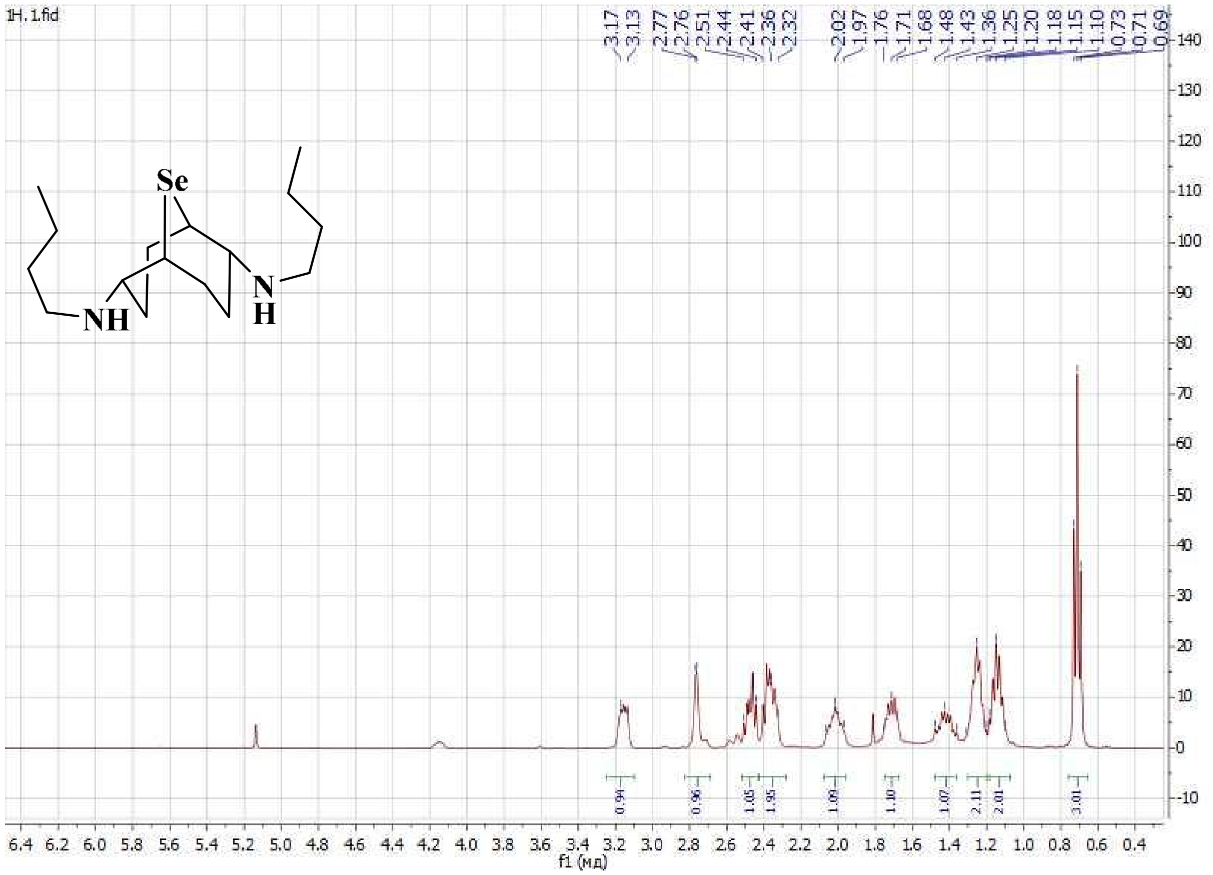

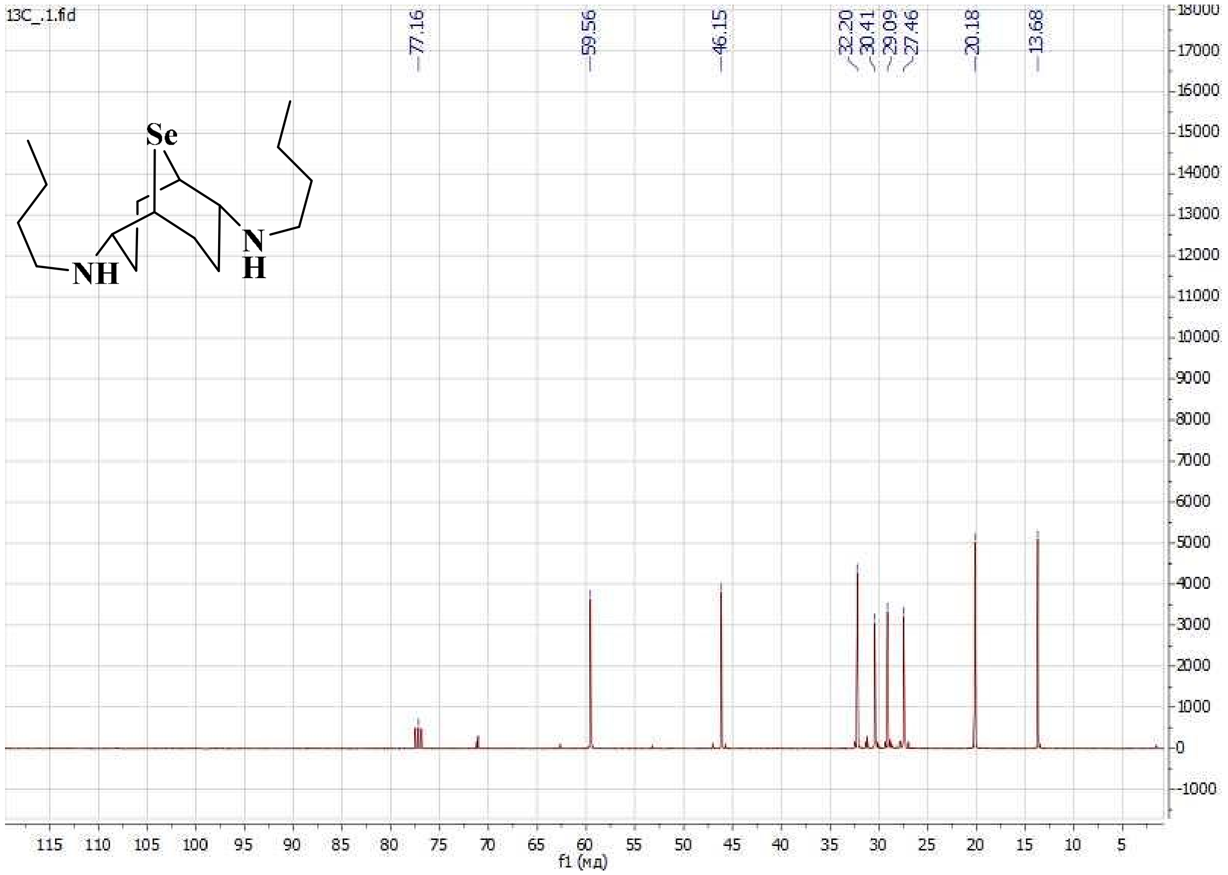

^{1}H- and ^{13}C-NMR spectra of compound 21 ($CDCl_3$)

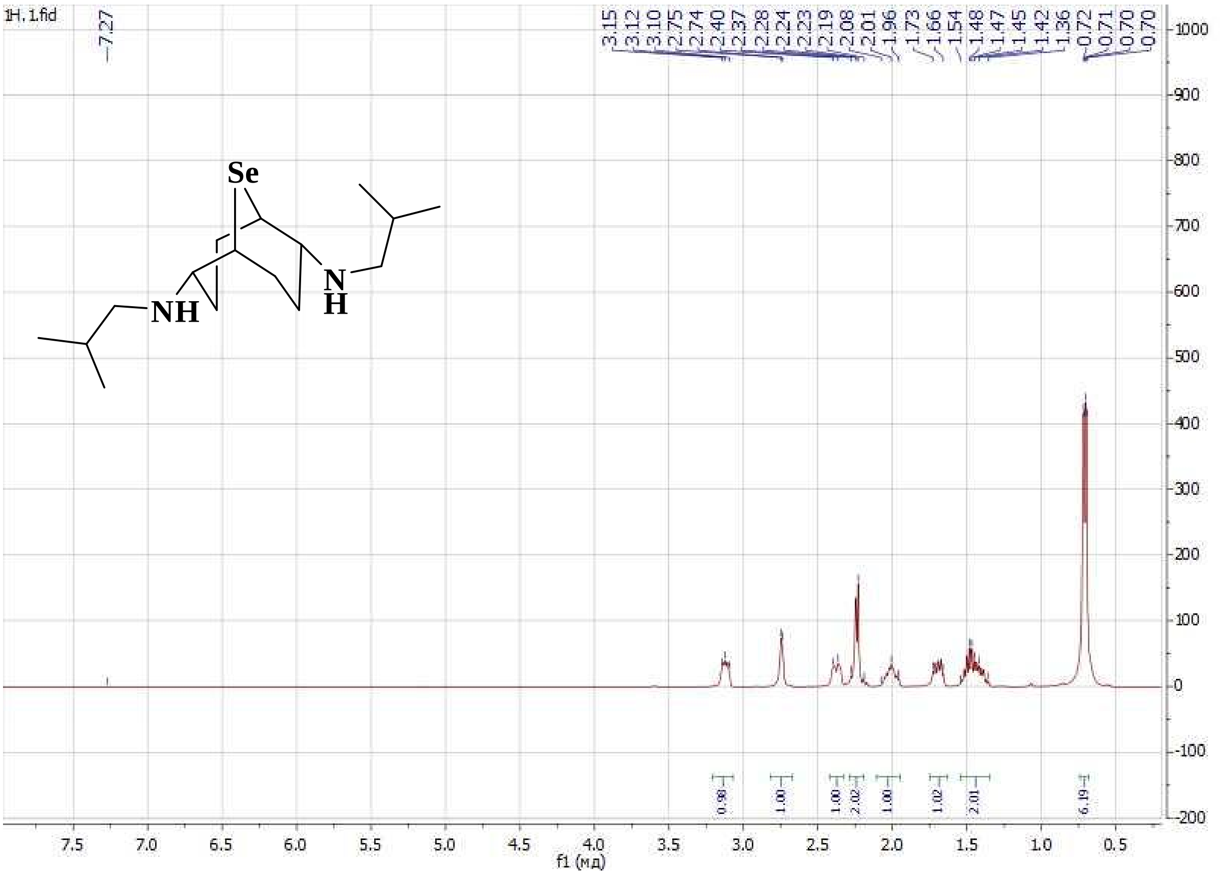

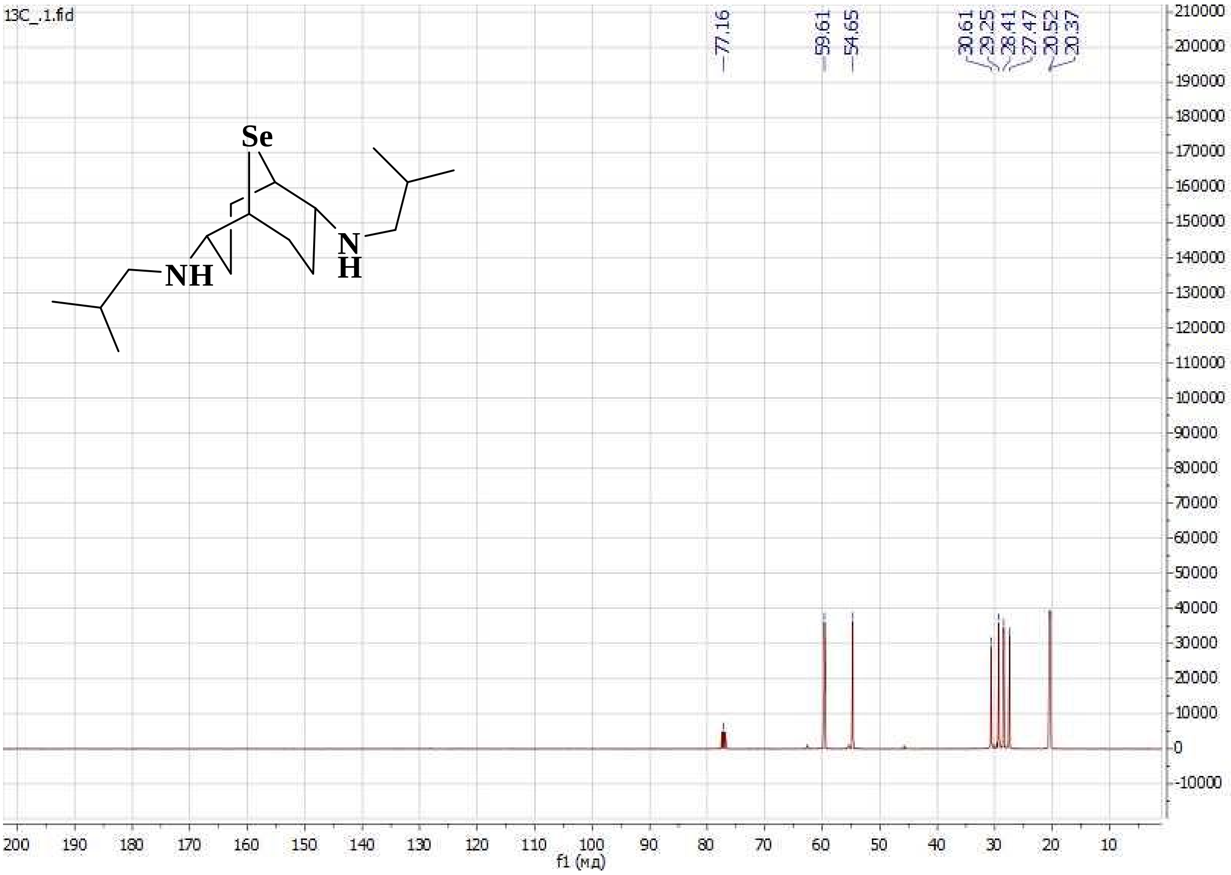

^{1}H- and ^{13}C-NMR spectra of compound 22 ($CDCl_3$)

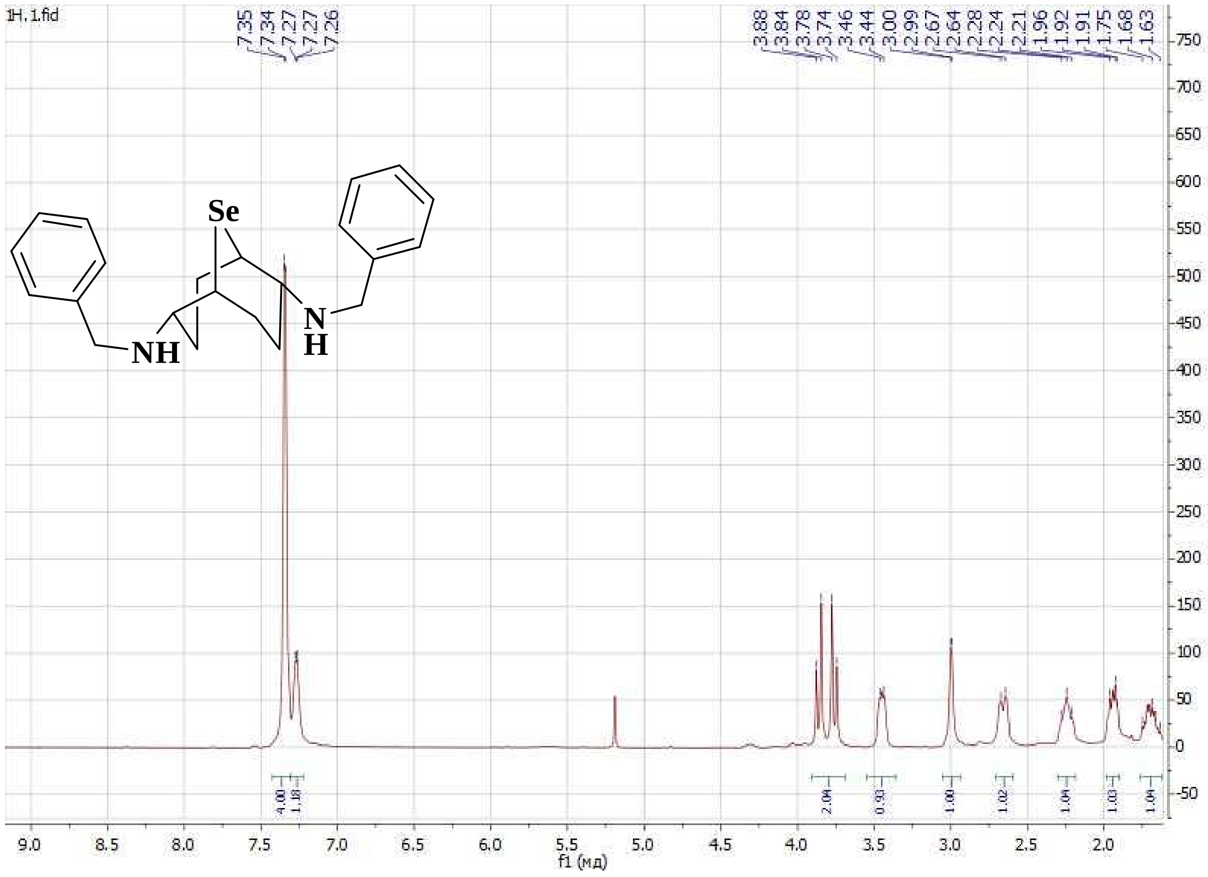

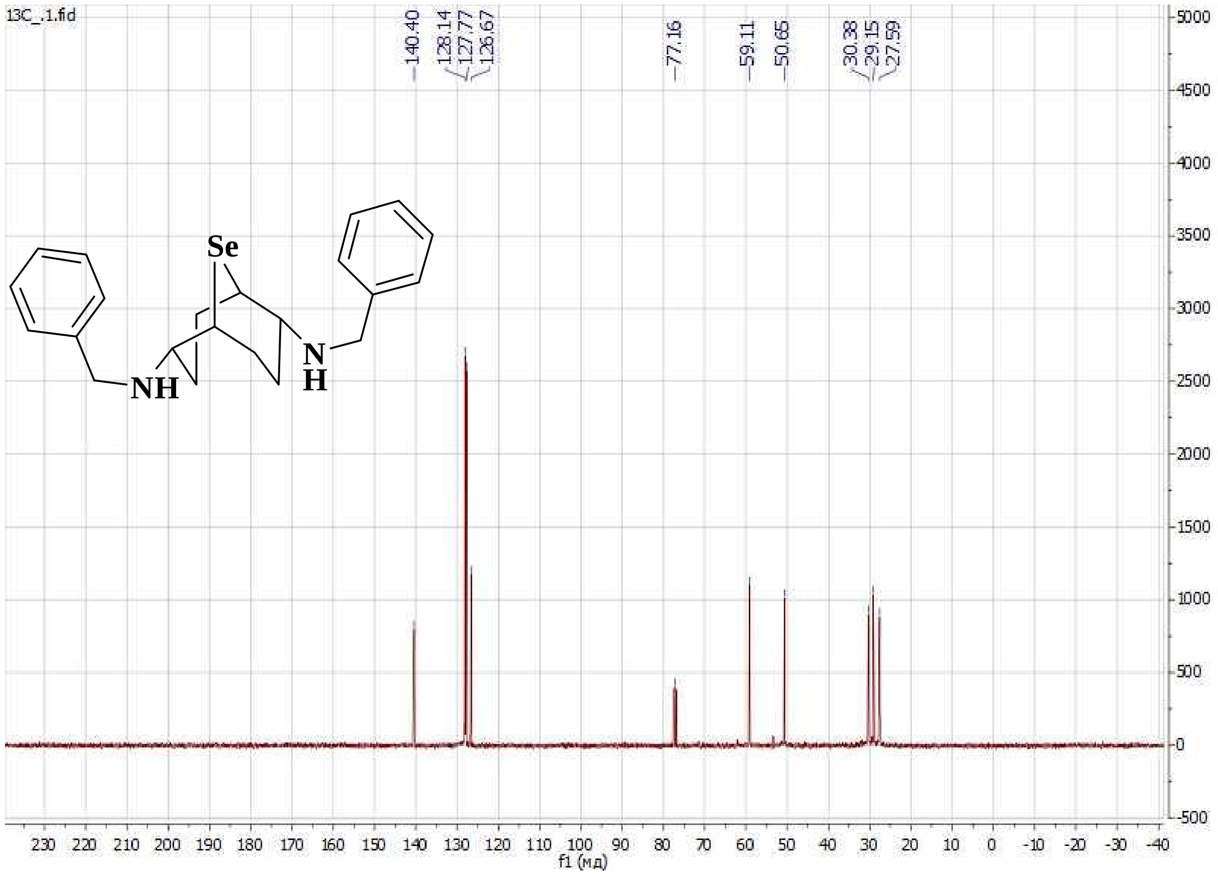

^{1}H- and ^{13}C-NMR spectra of compound 23 ($CDCl_3$)

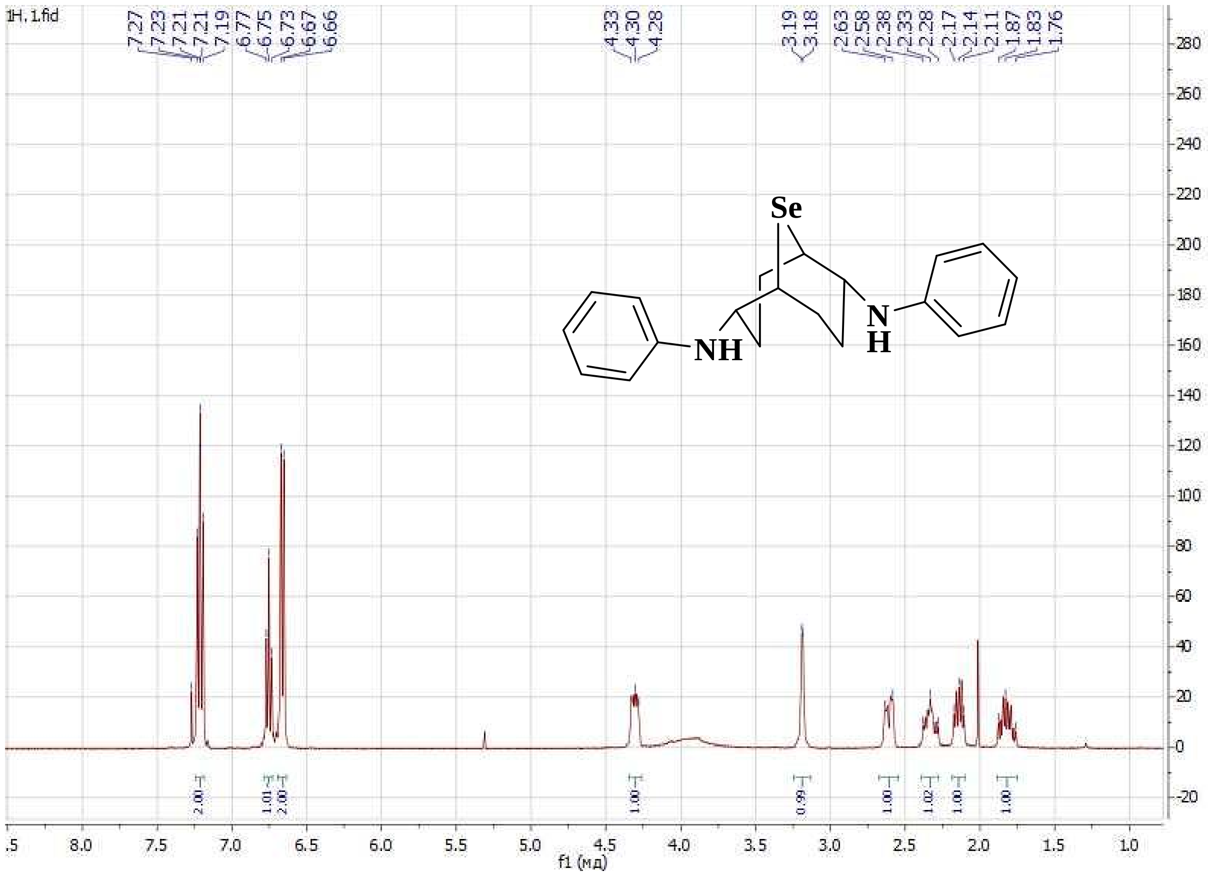

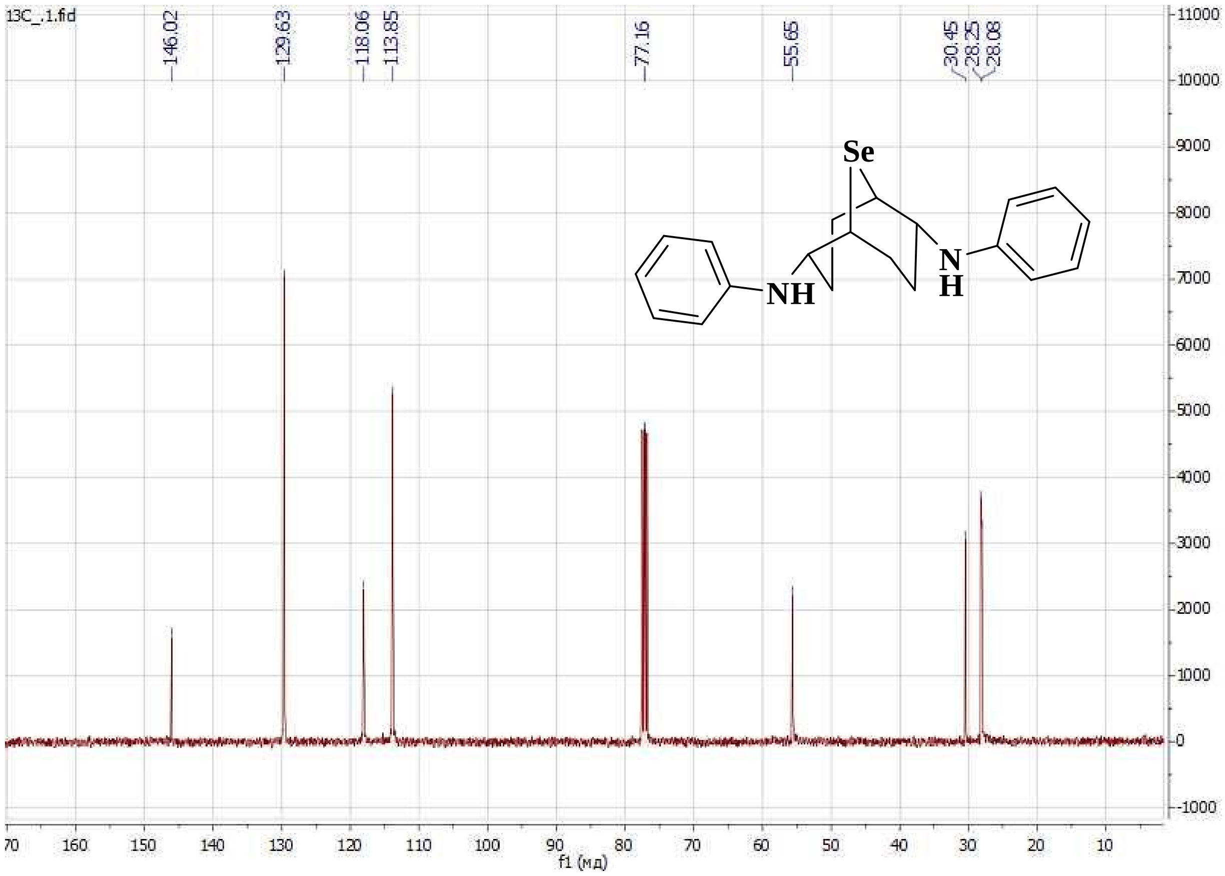

^{1}H- and ^{13}C-NMR spectra of compound 24 ($CDCl_3$)

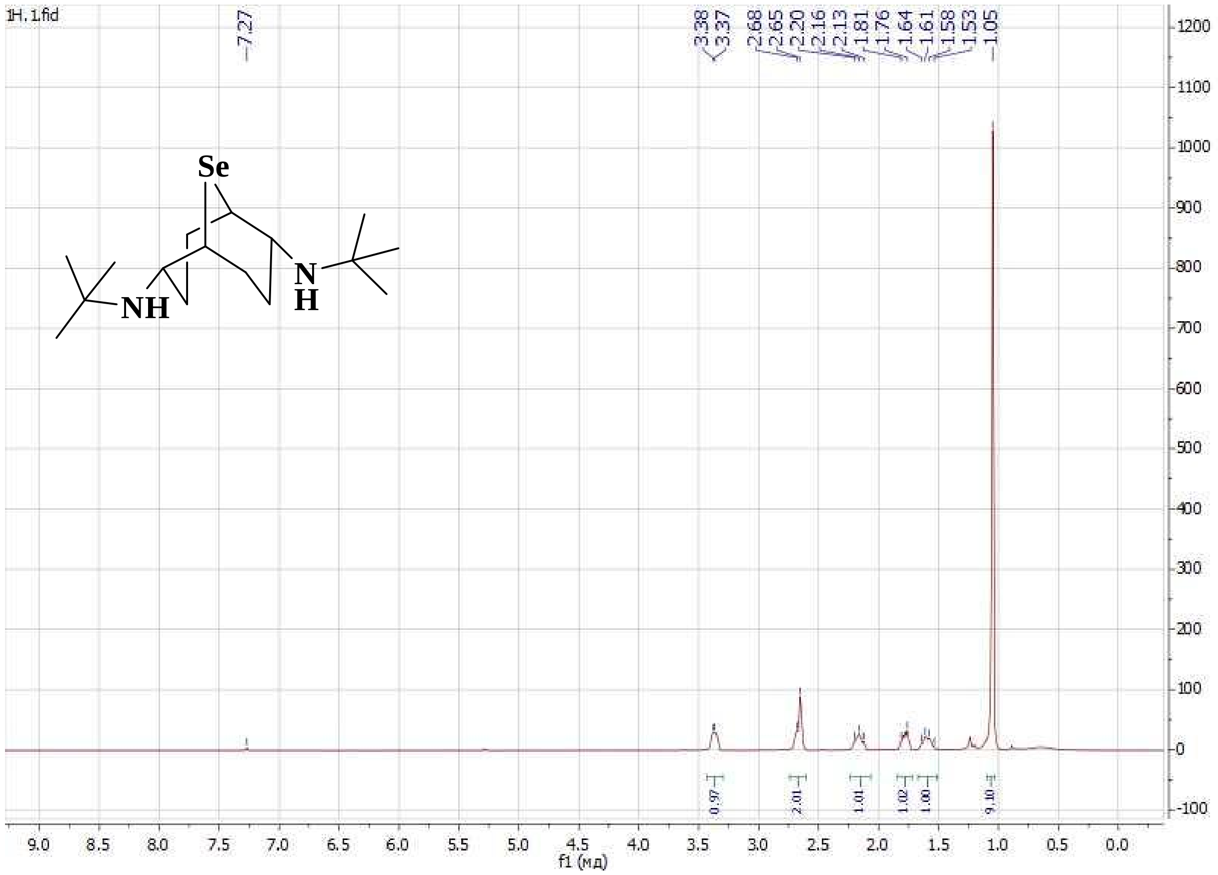

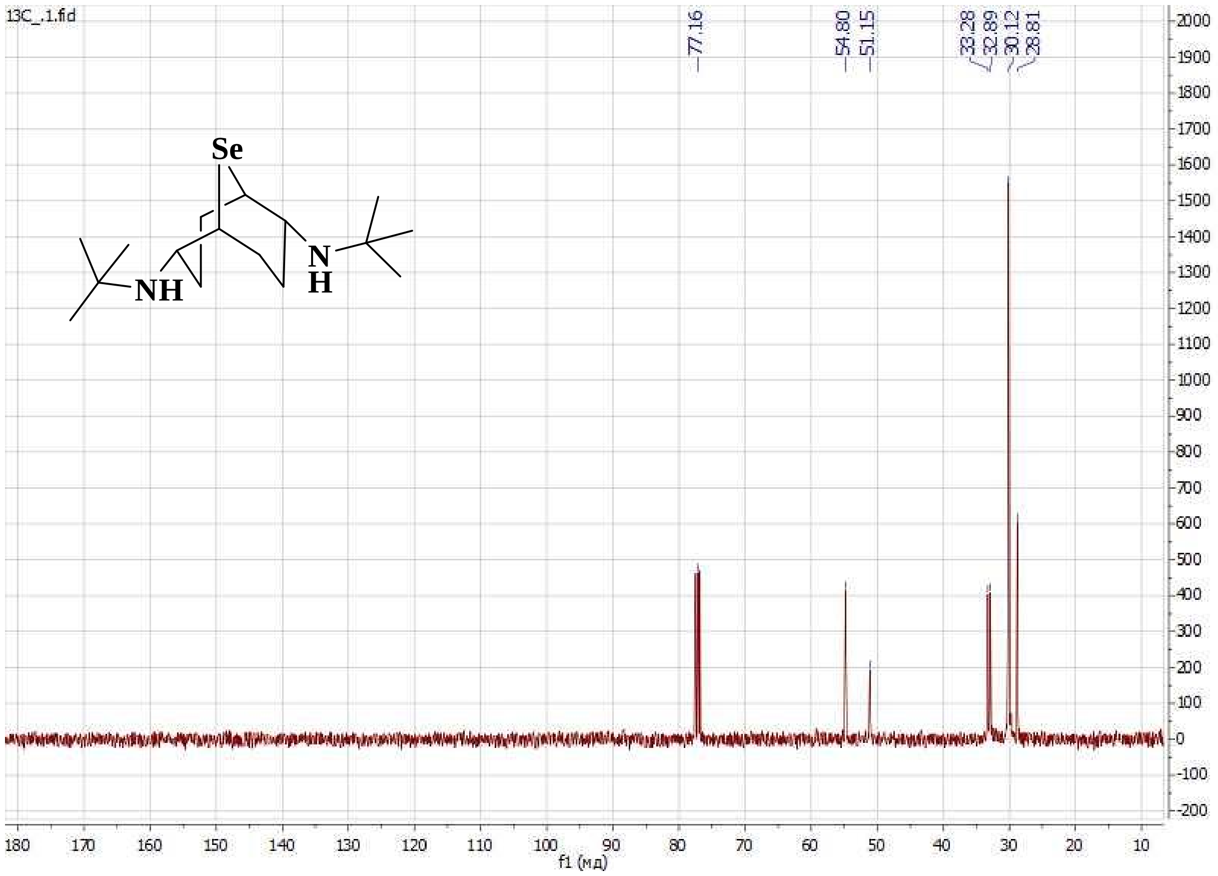

^{1}H- and ^{13}C-NMR spectra of compound 25 ($CDCl_3$)

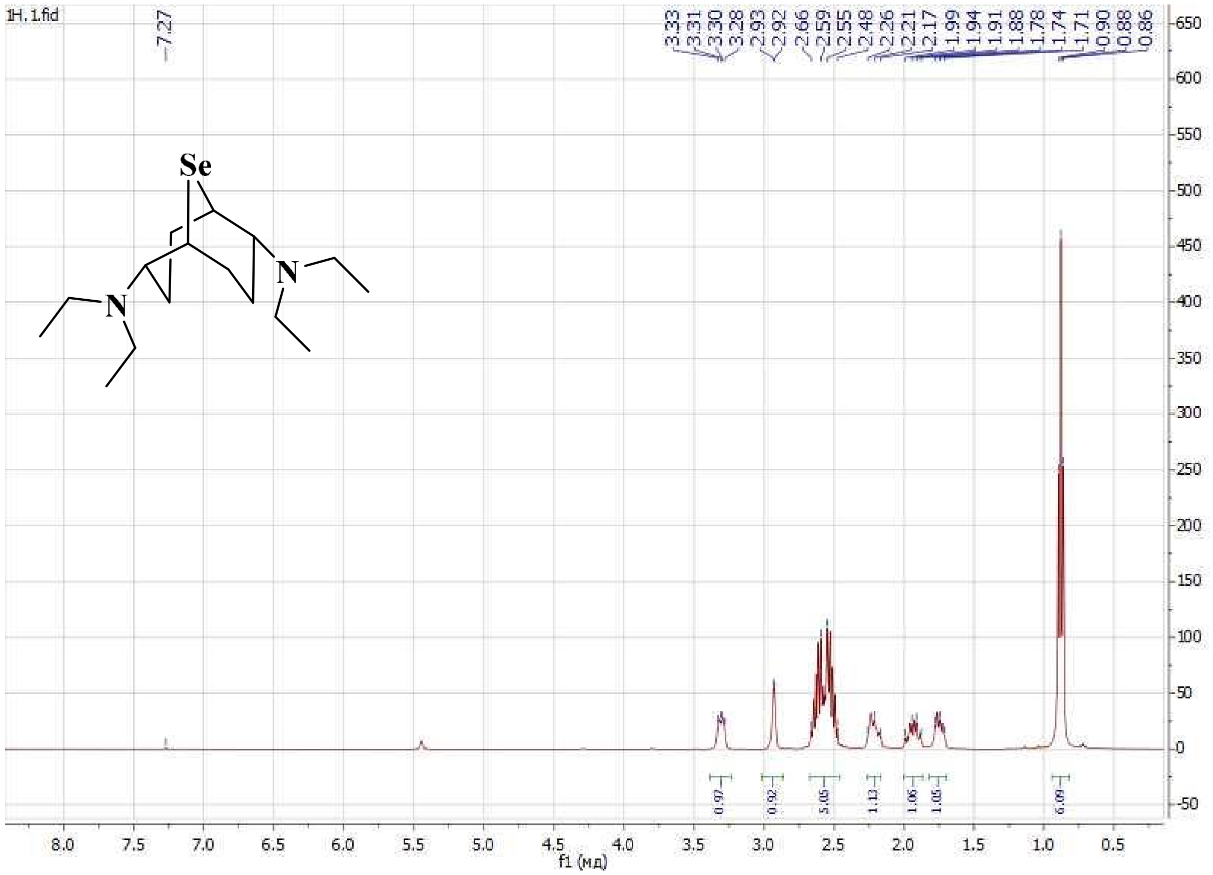

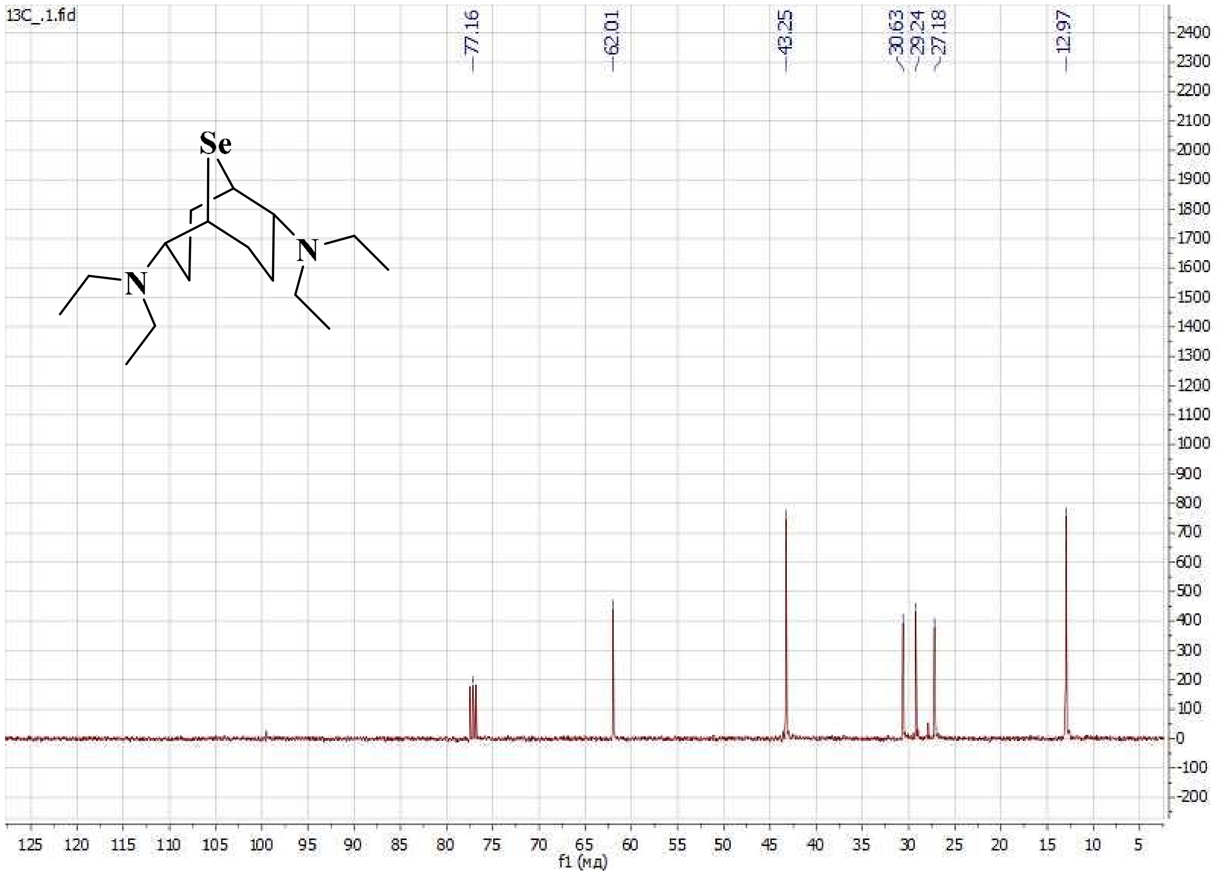

^{1}H- and ^{13}C-NMR spectra of compound 26 ($CDCl_3$)

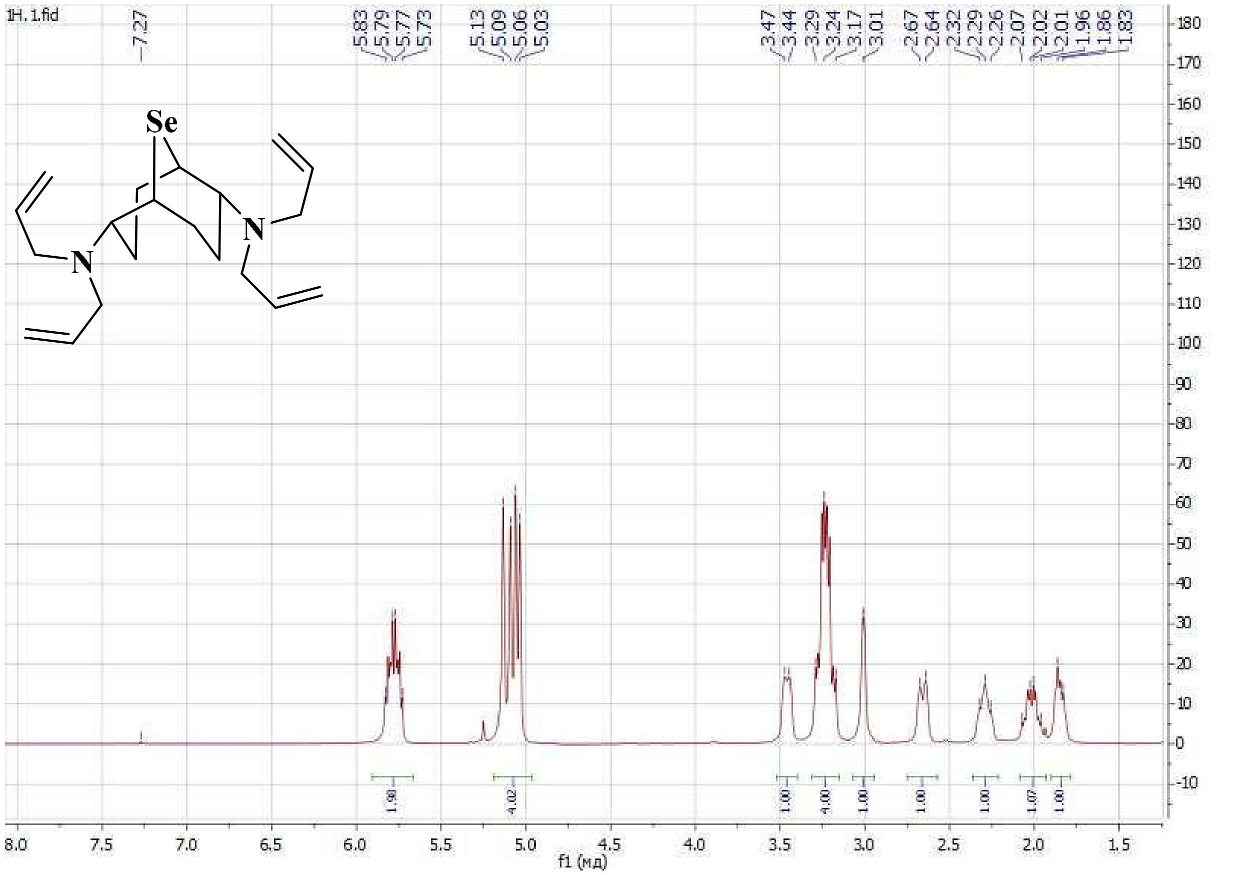

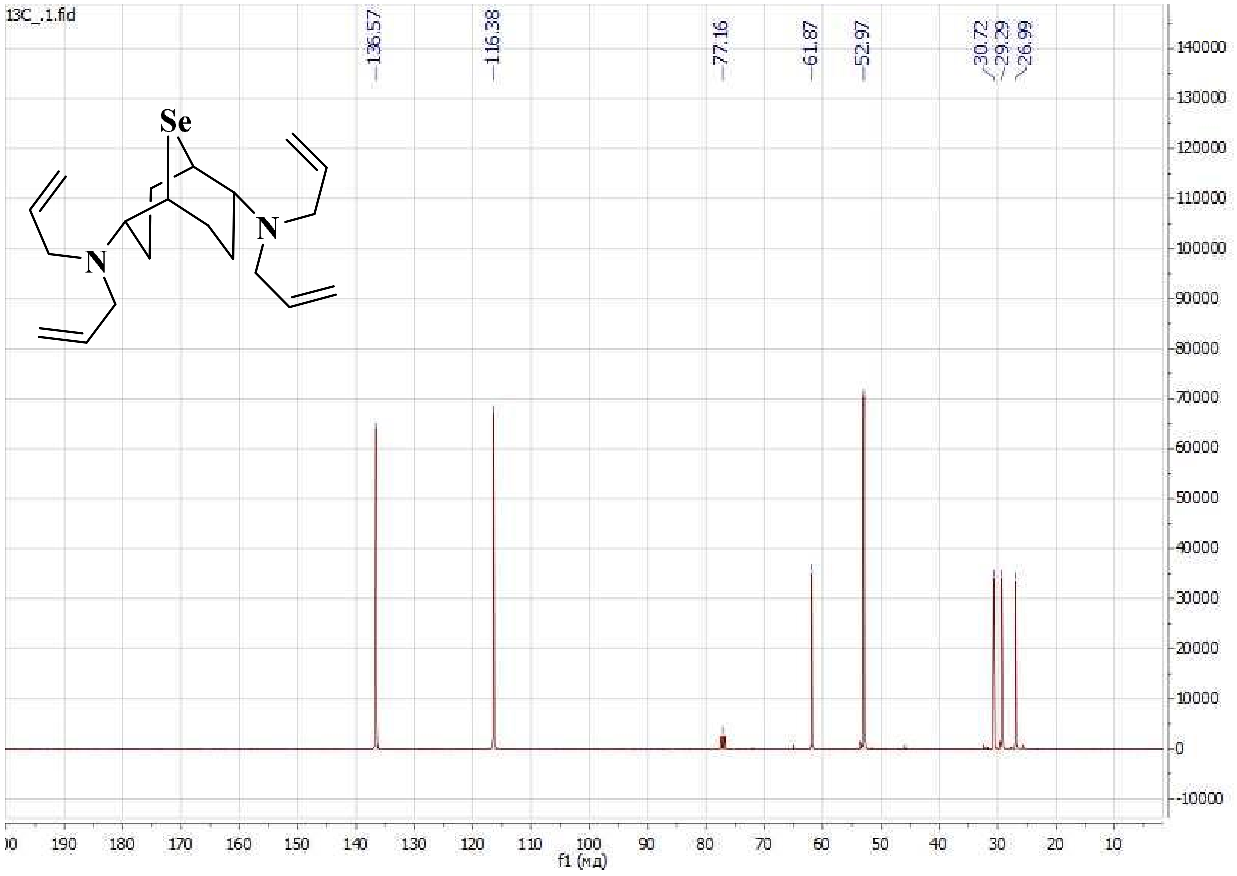

^{1}H- and ^{13}C-NMR spectra of compound 27 ($CDCl_3$)

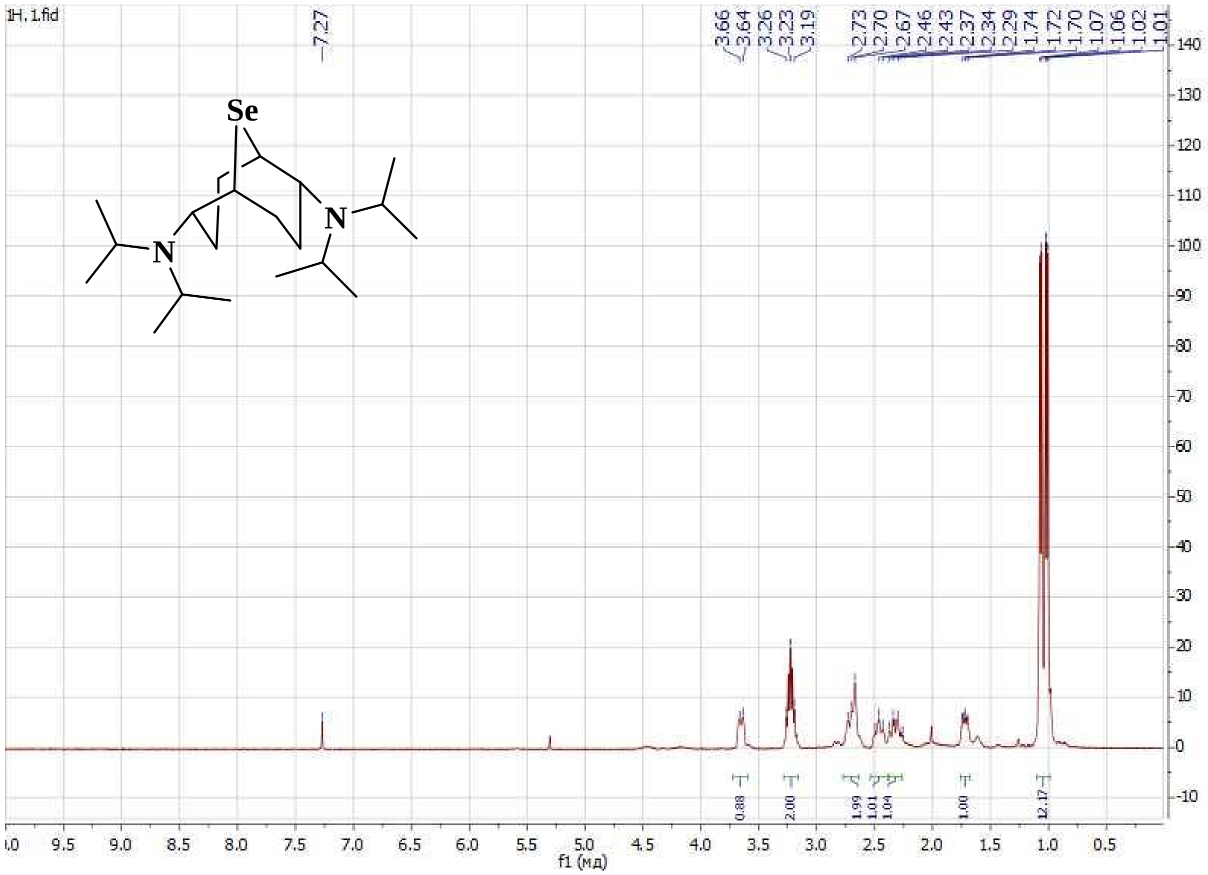

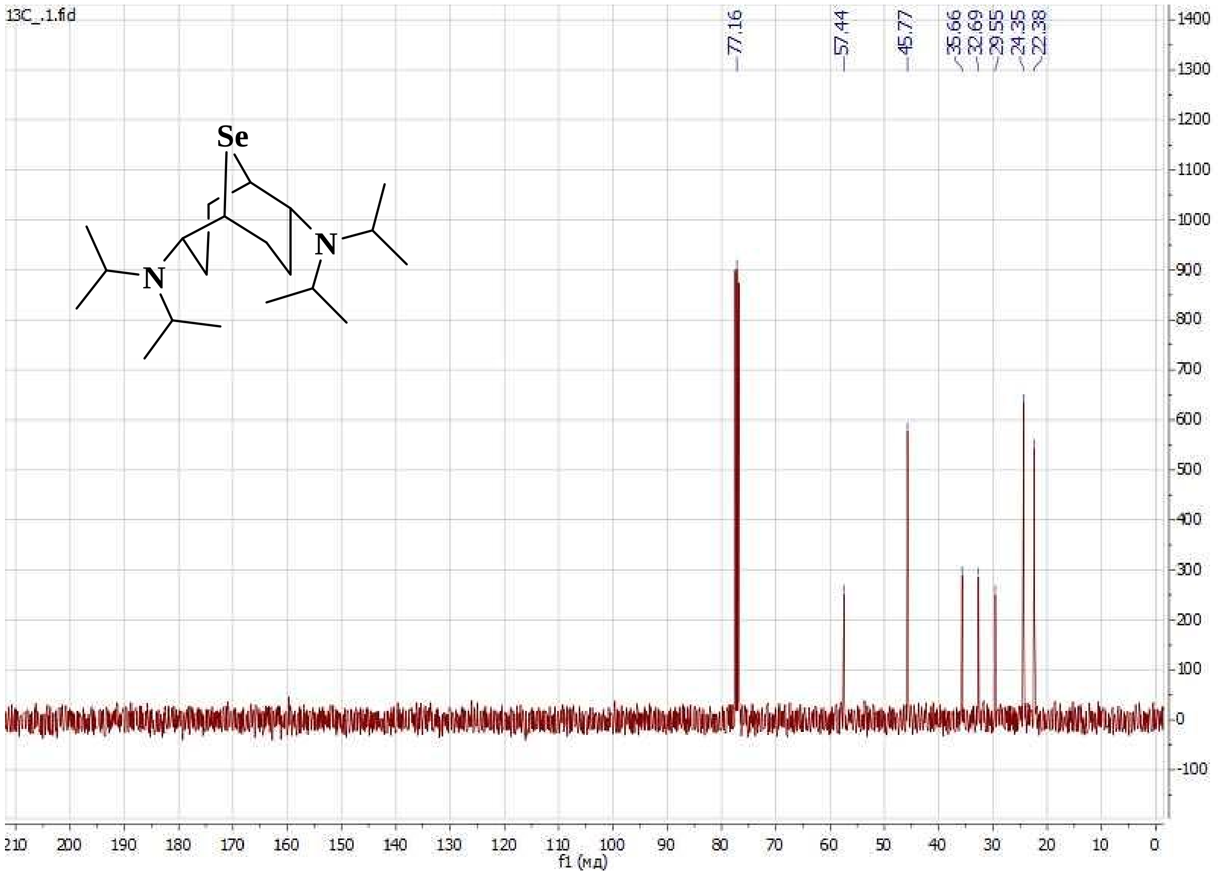

^{1}H- and ^{13}C-NMR spectra of compound 28 (CDCl3)

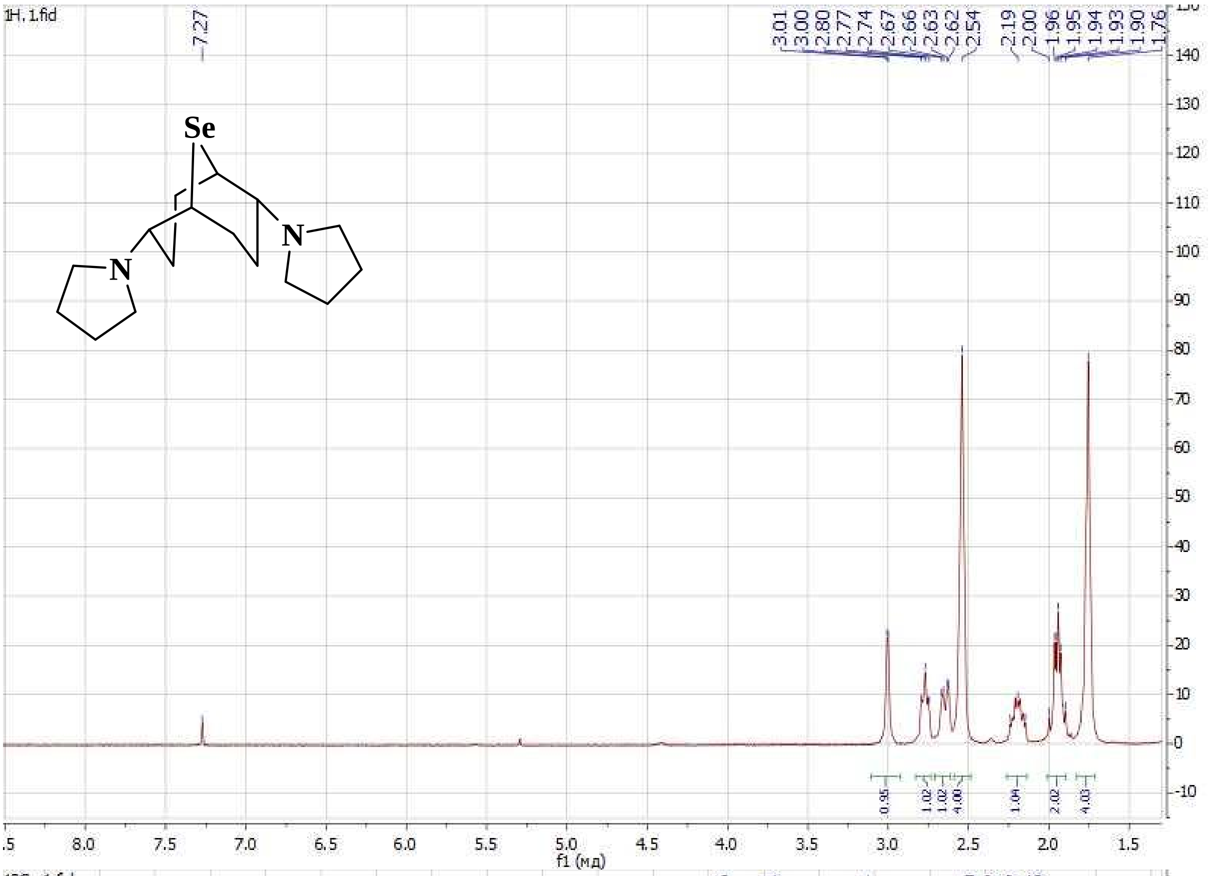

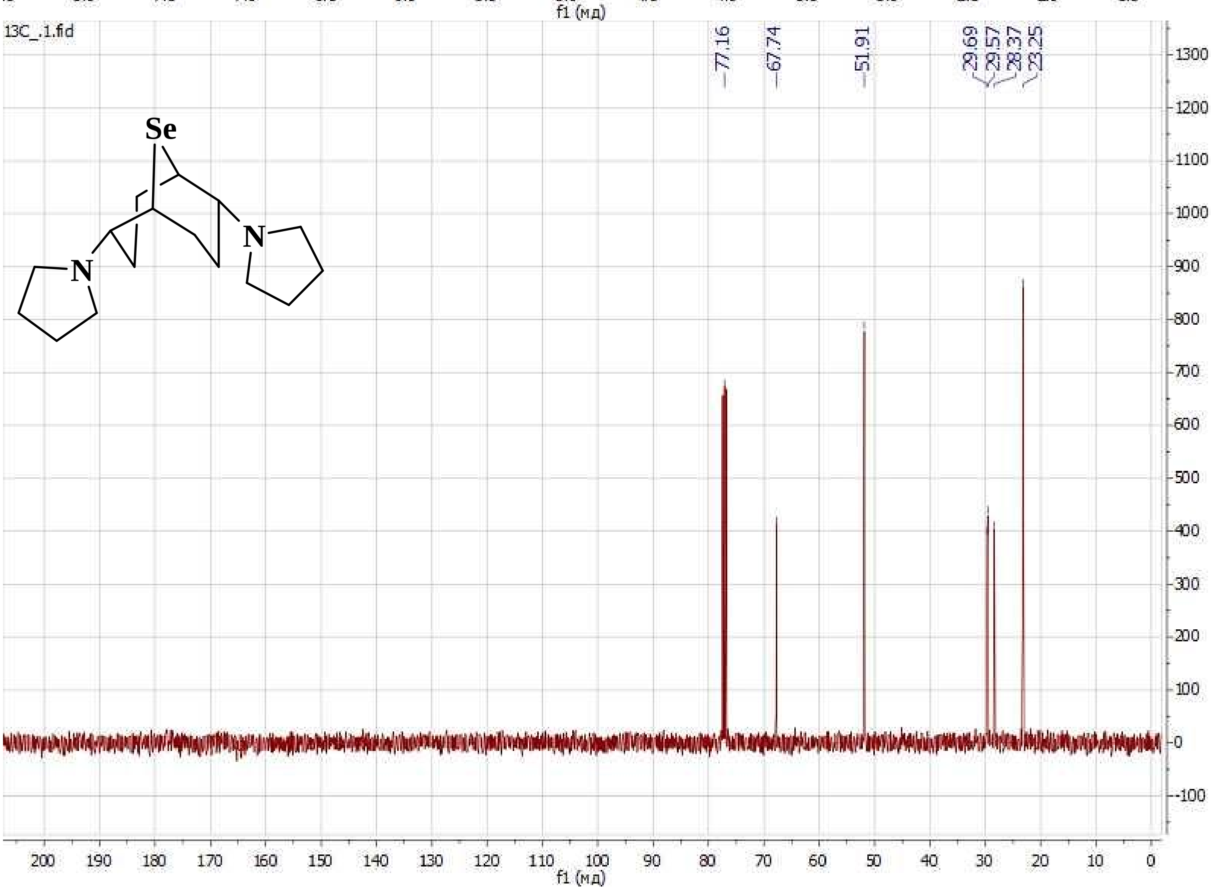

^{1}H- and ^{13}C-NMR spectra of compound 29 ($CDCl_3$)

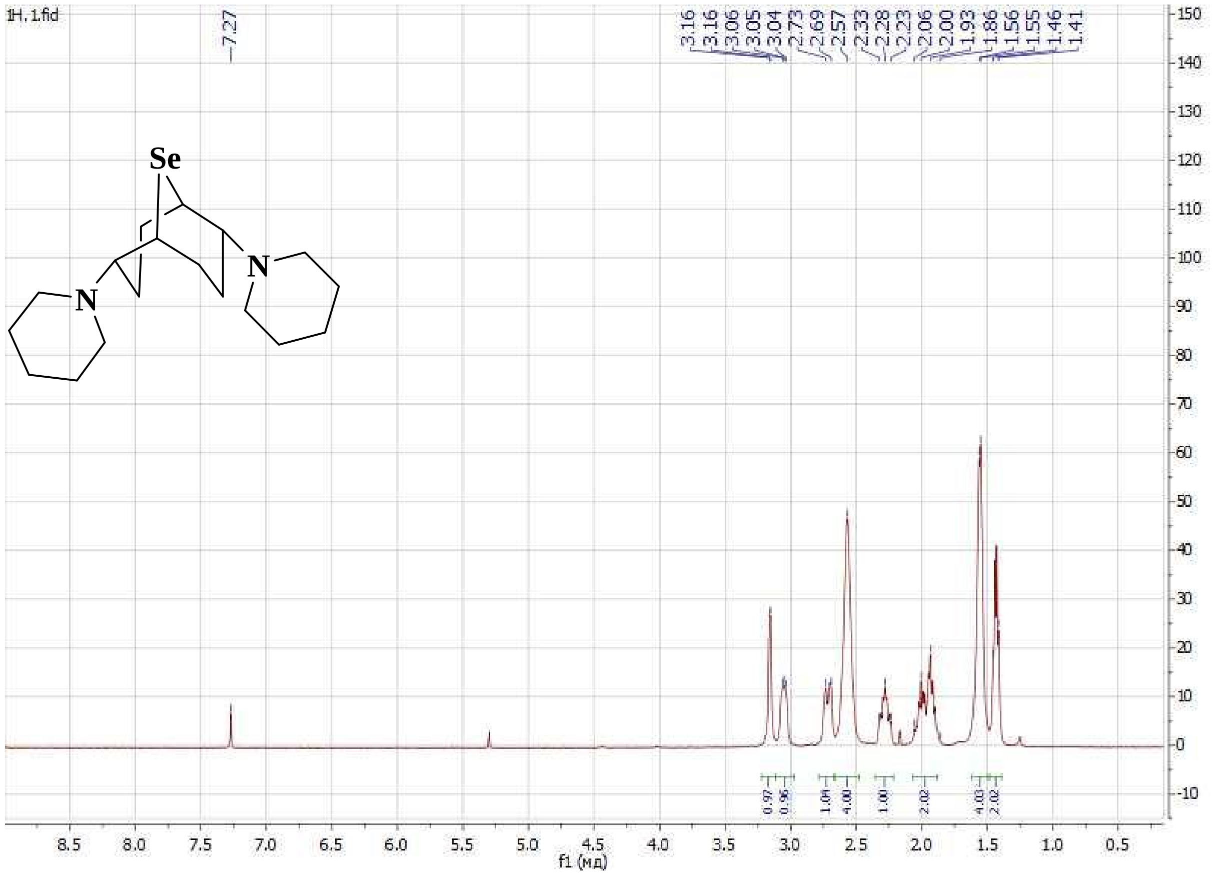

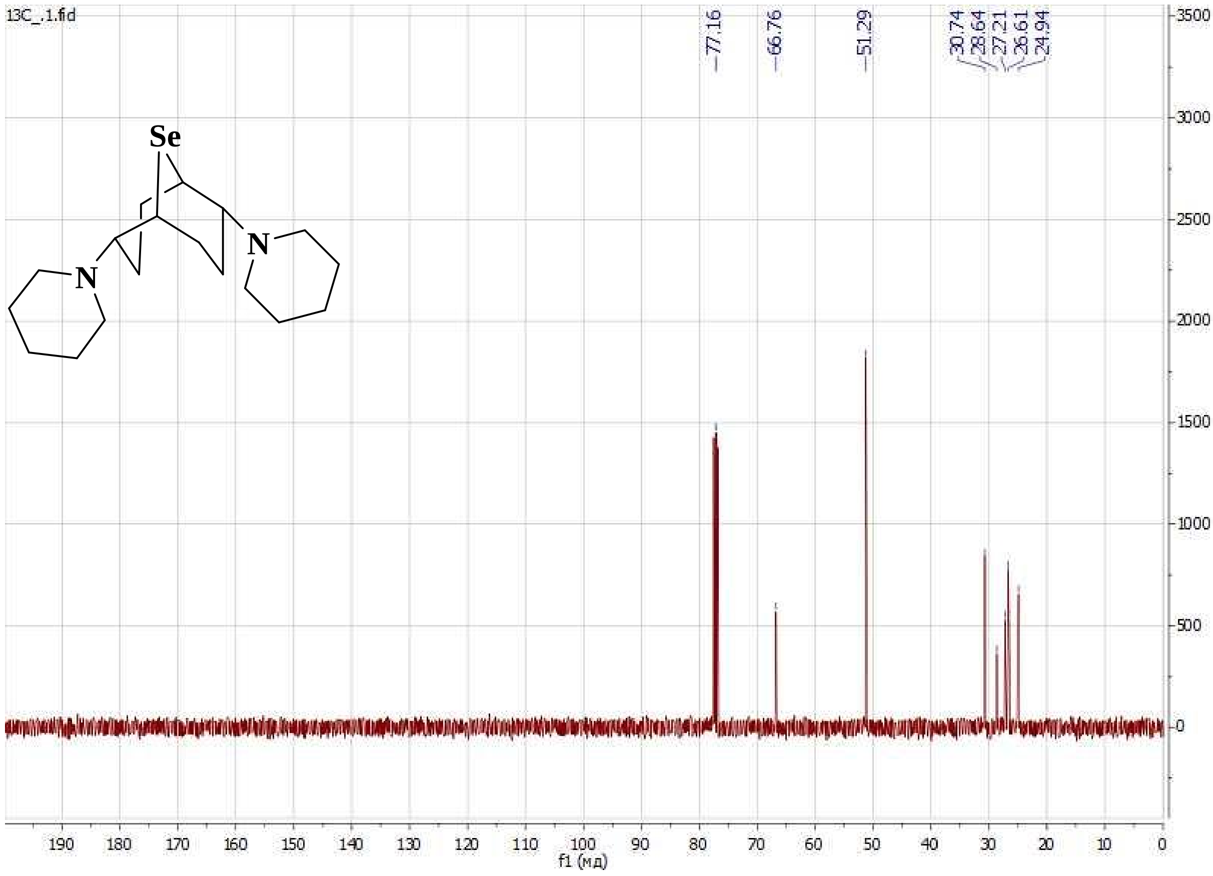

^{1}H- and ^{13}C-NMR spectra of compound 30 ($CDCl_3$)

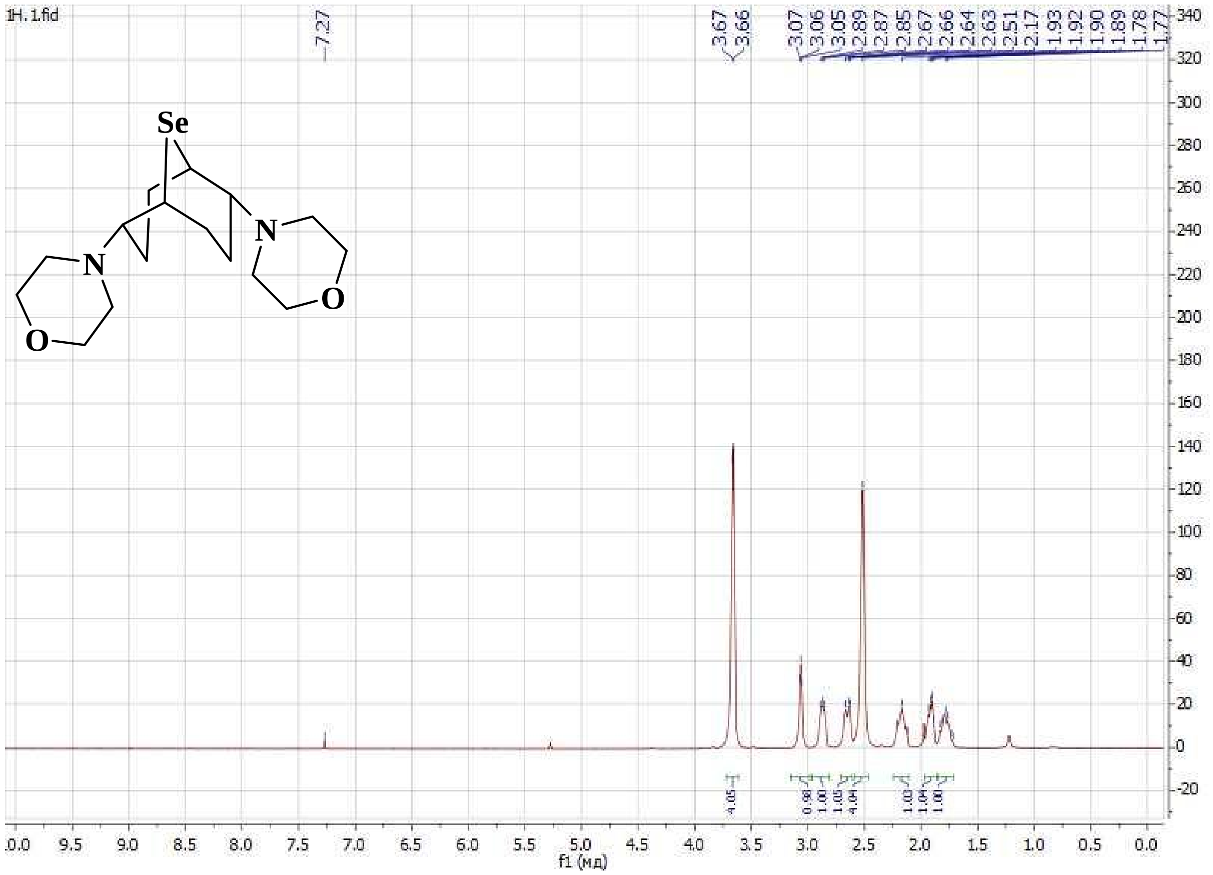

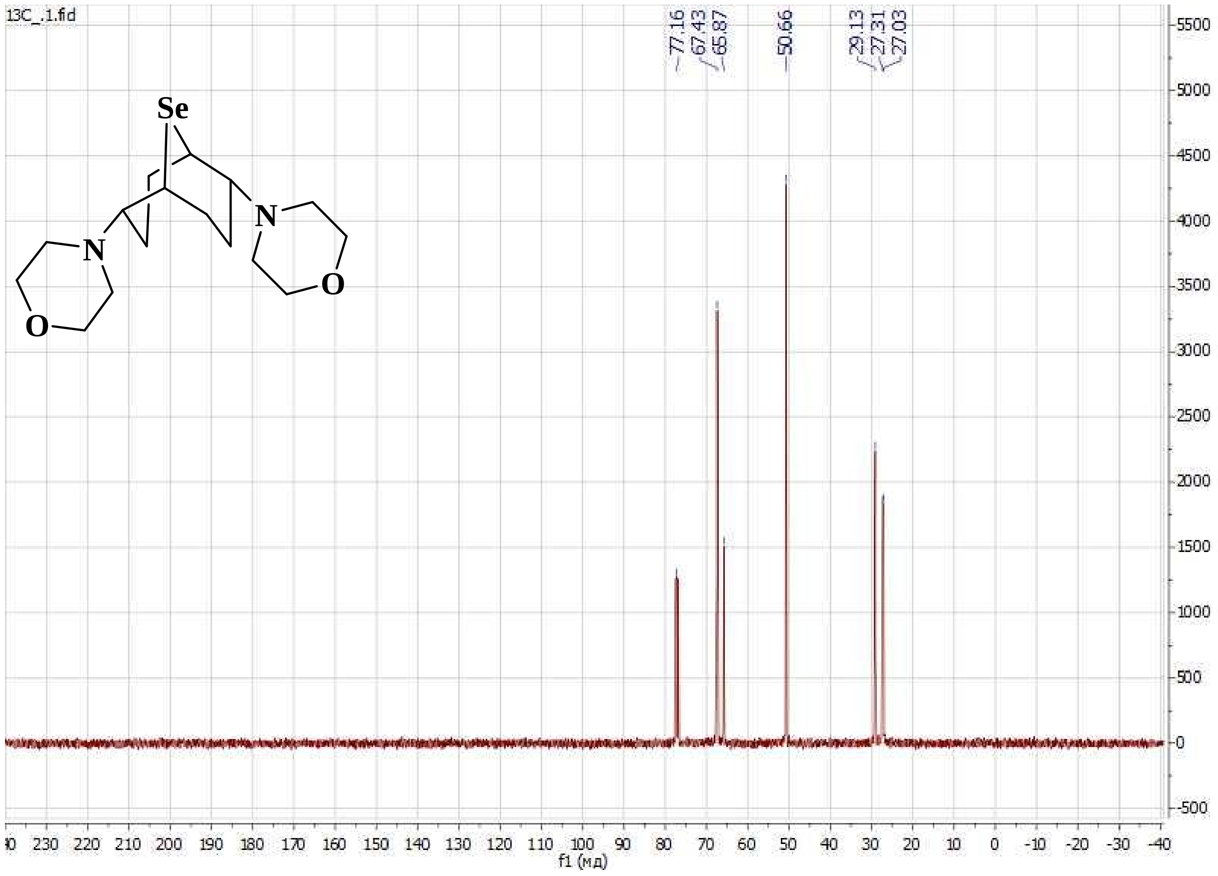

^{1}H- and ^{13}C-NMR spectra of compound 31 ($CDCl_3$)

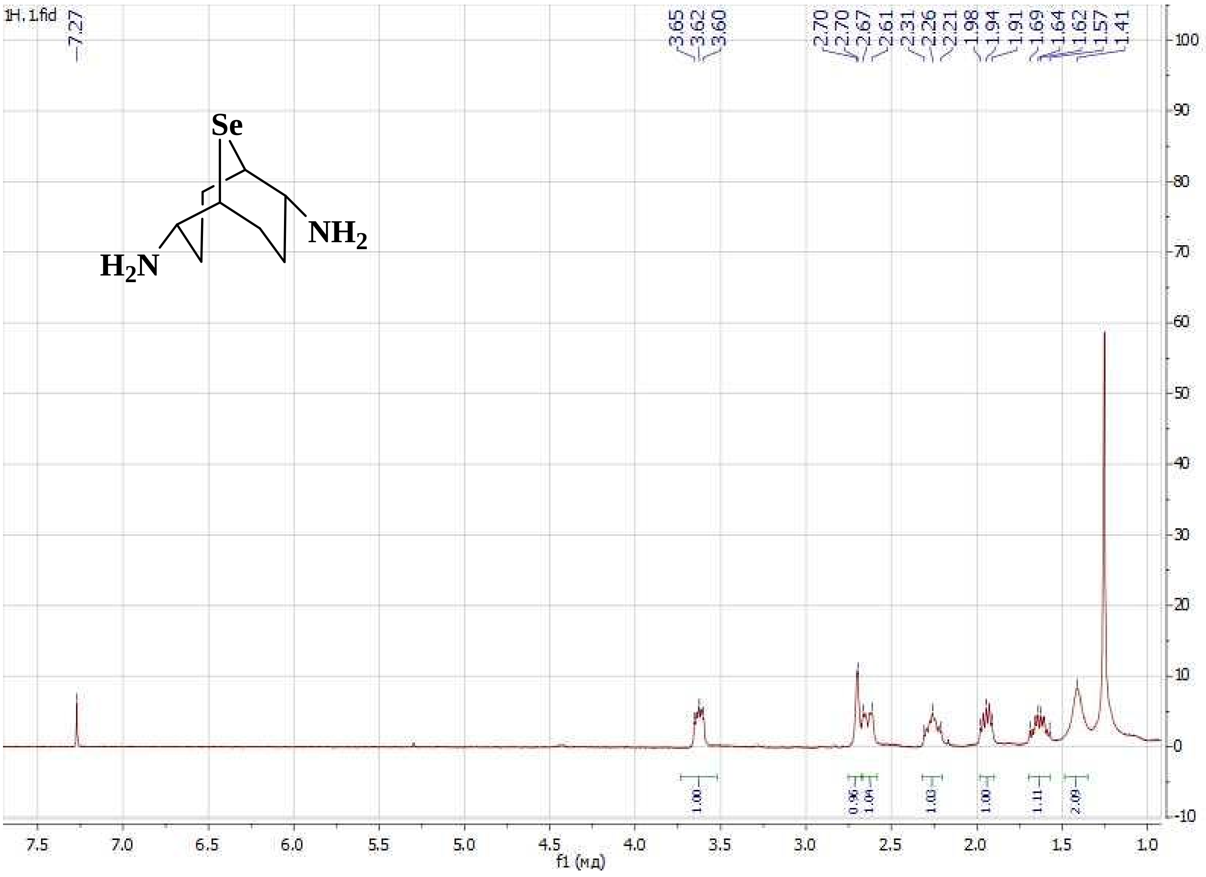

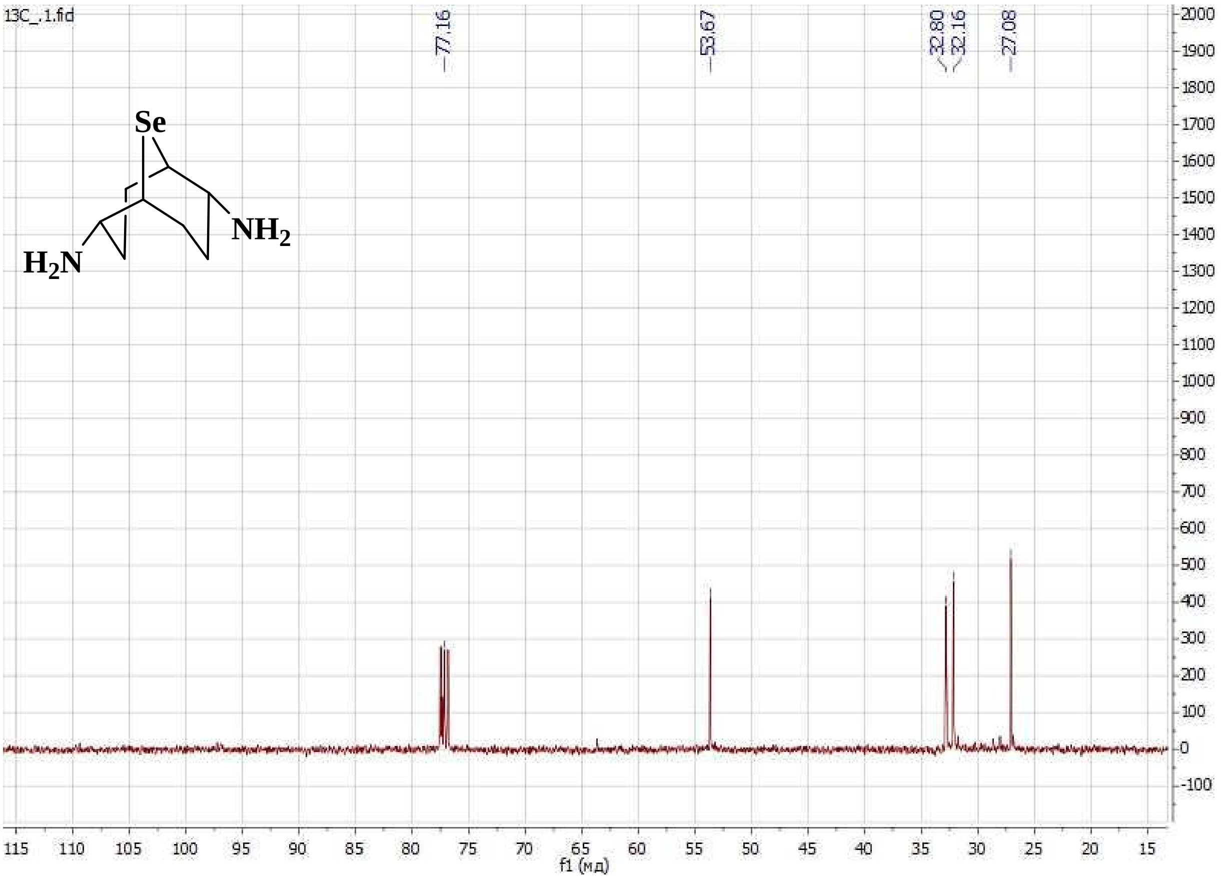

^{1}H- and ^{13}C-NMR spectra of compound 36 ($CDCl_3$)

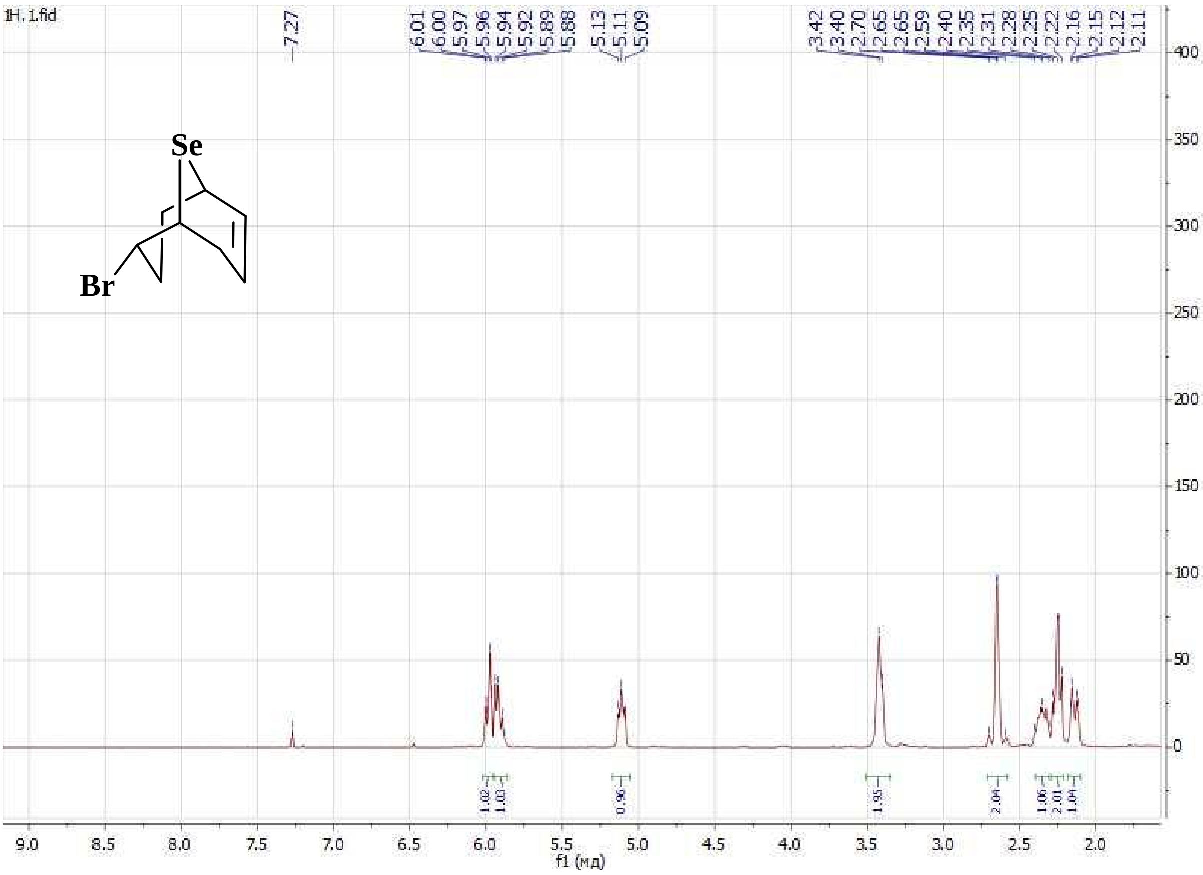

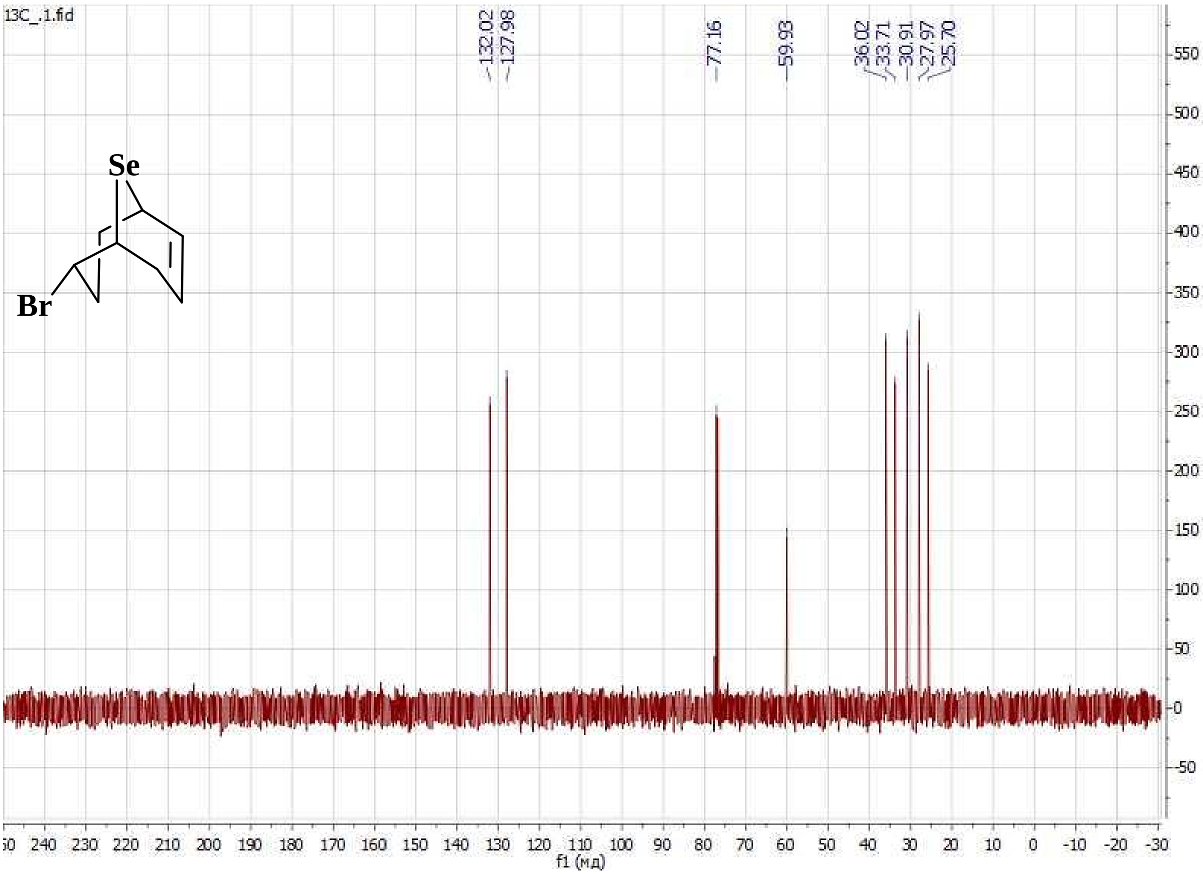

^{1}H- and ^{13}C-NMR spectra of compound 37 ($CDCl_3$)

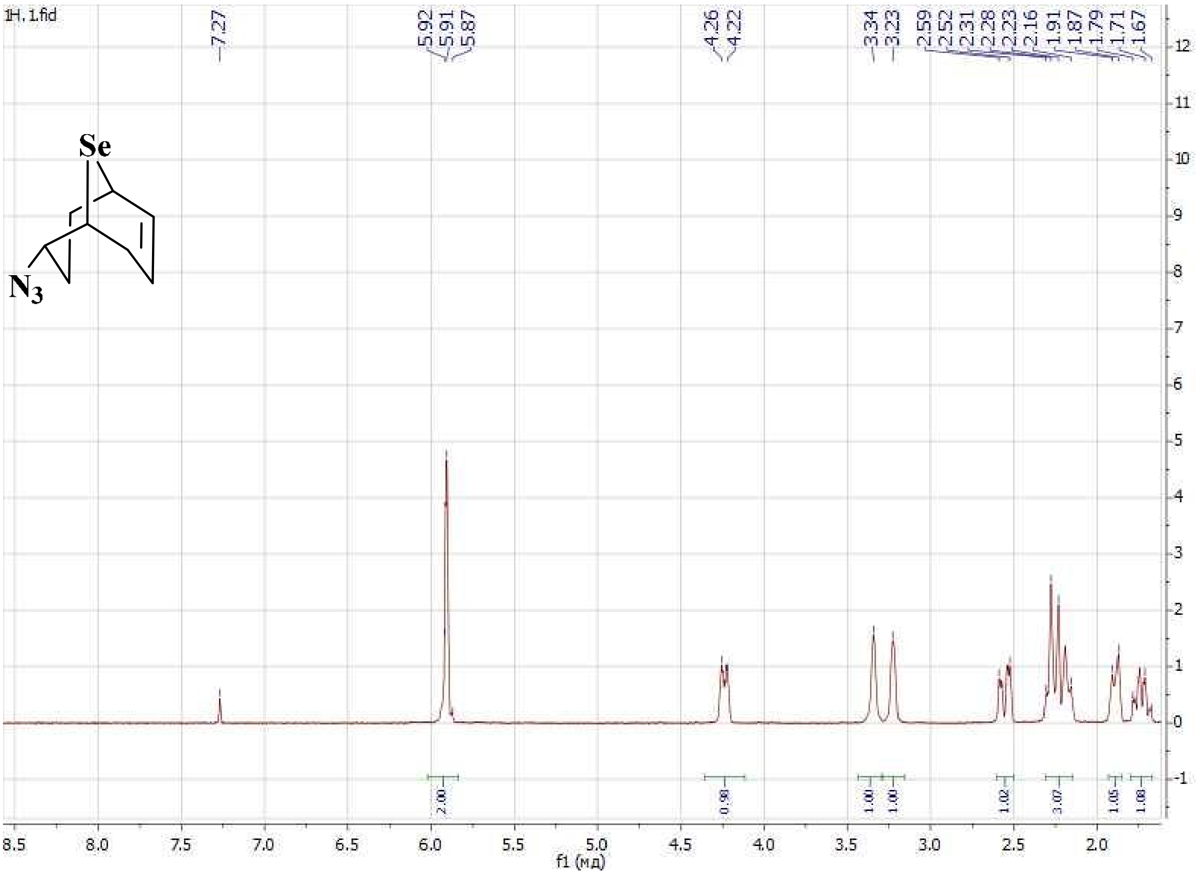

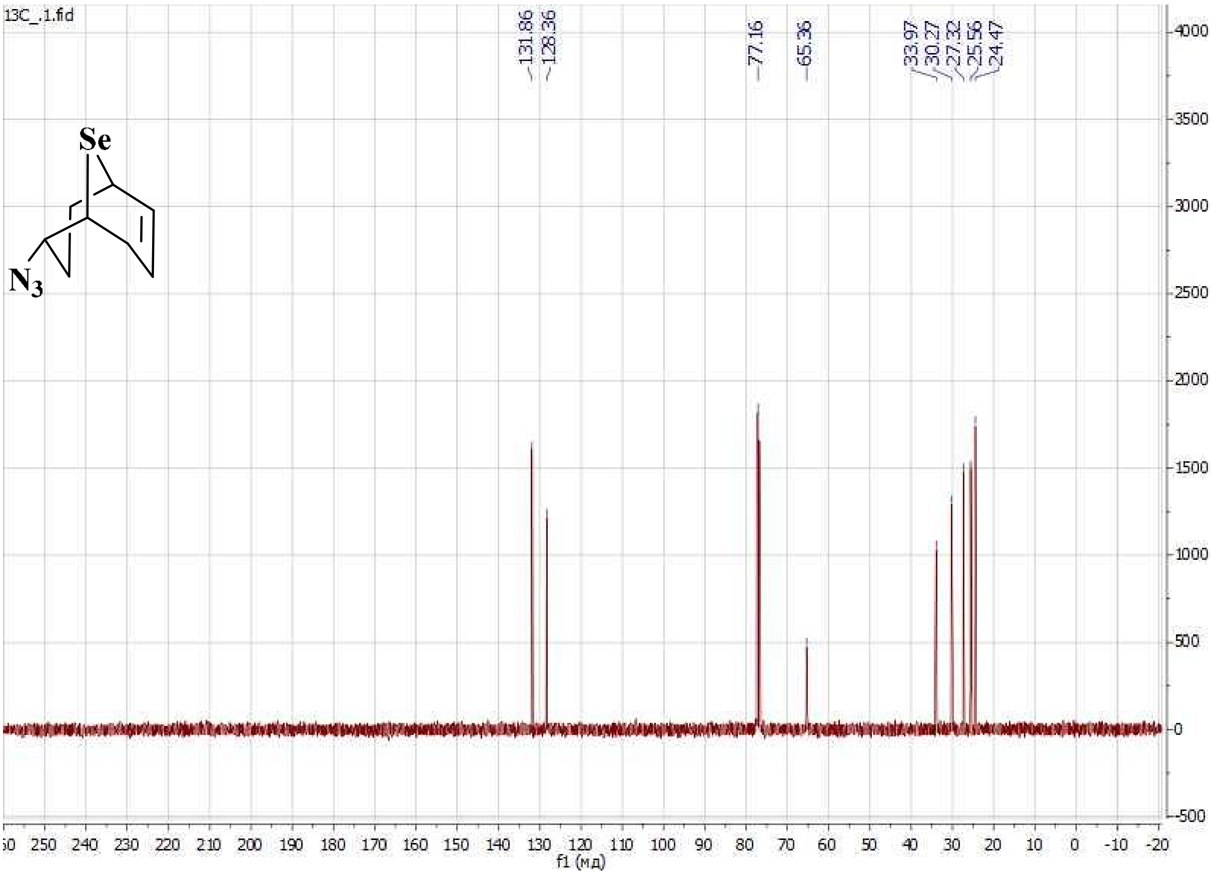

^{1}H- and ^{13}C-NMR spectra of compound 38

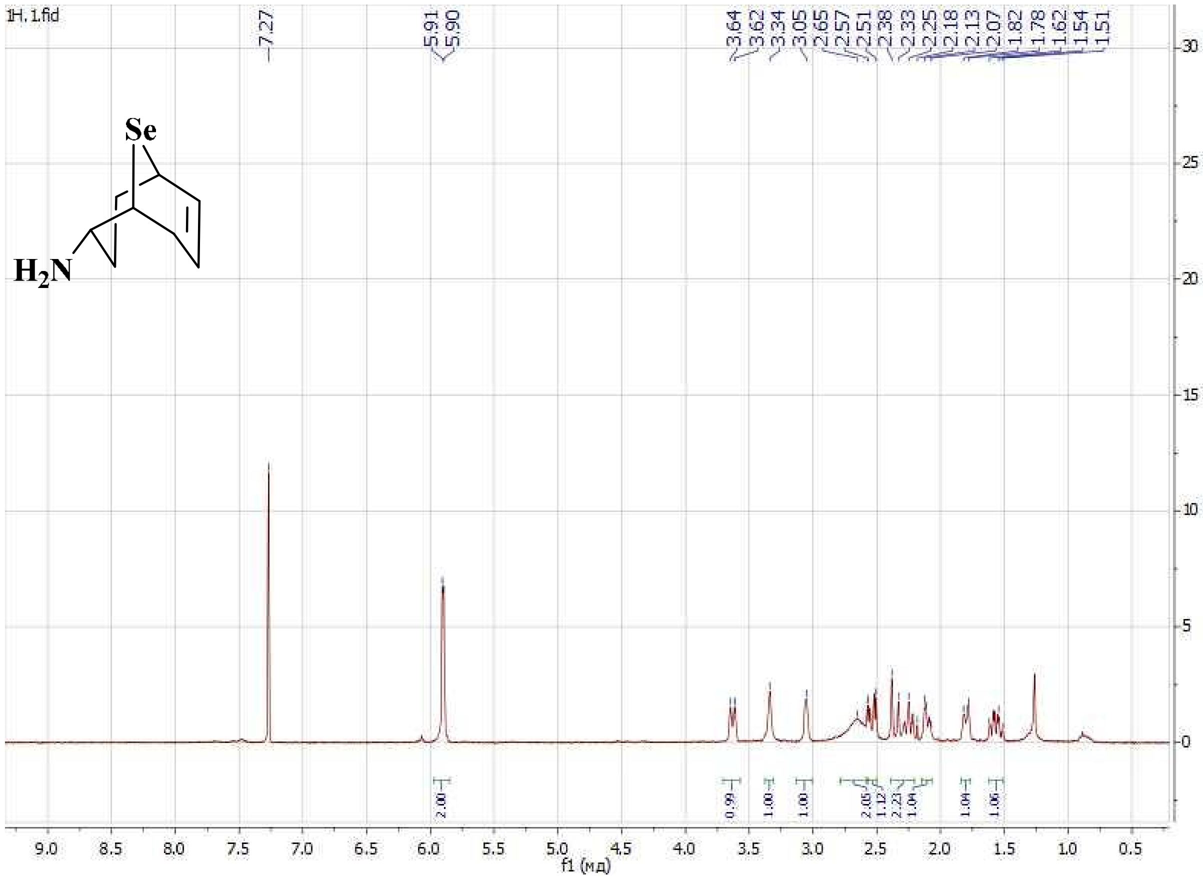

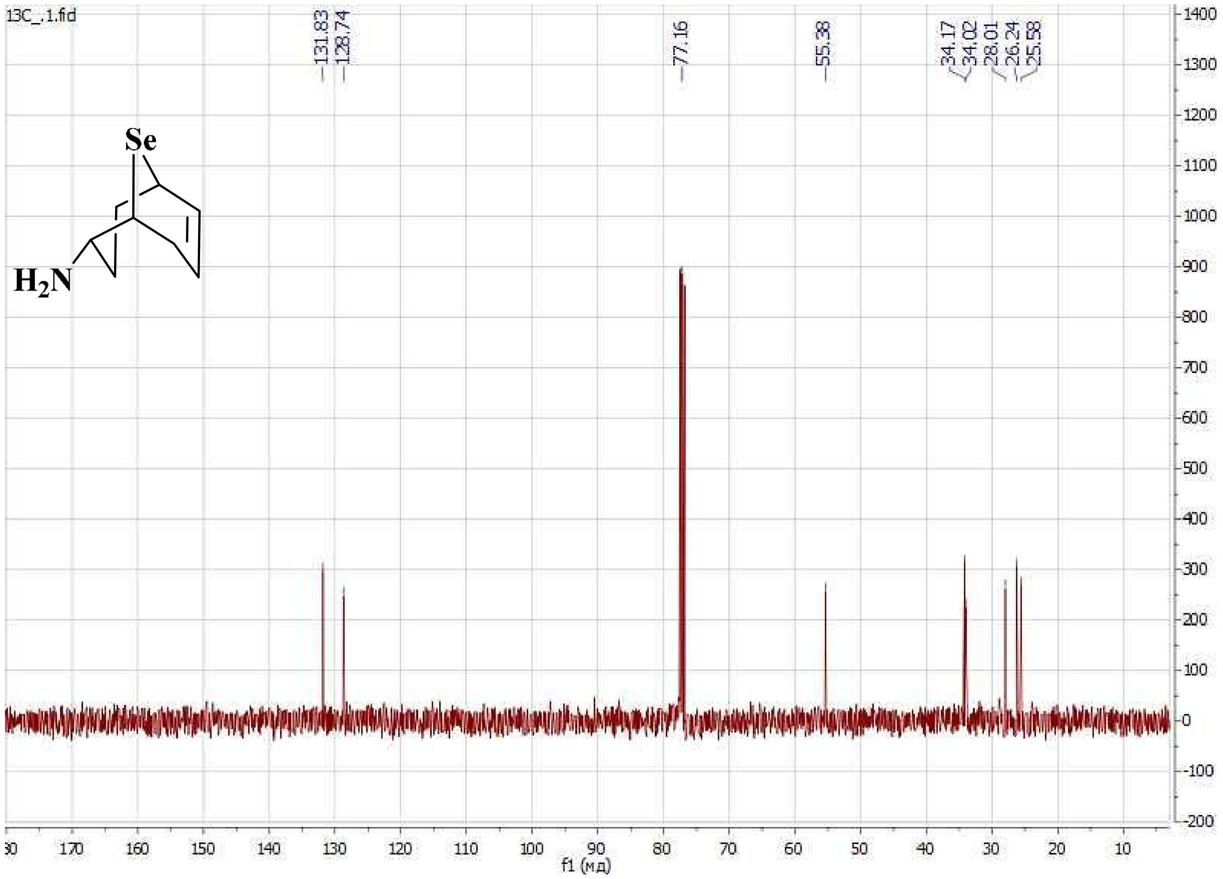

^{1}H- and ^{13}C-NMR spectra of compound 39 ($CDCl_3$)

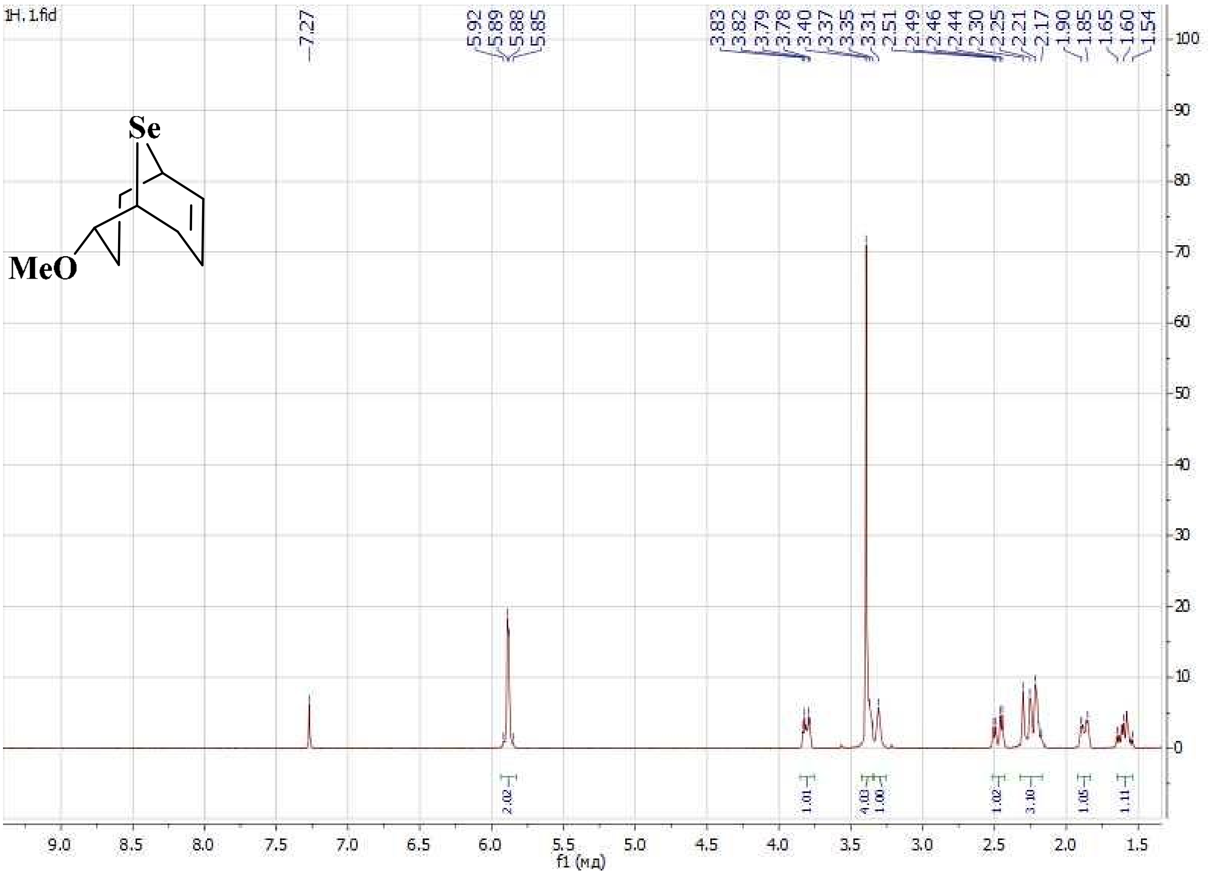

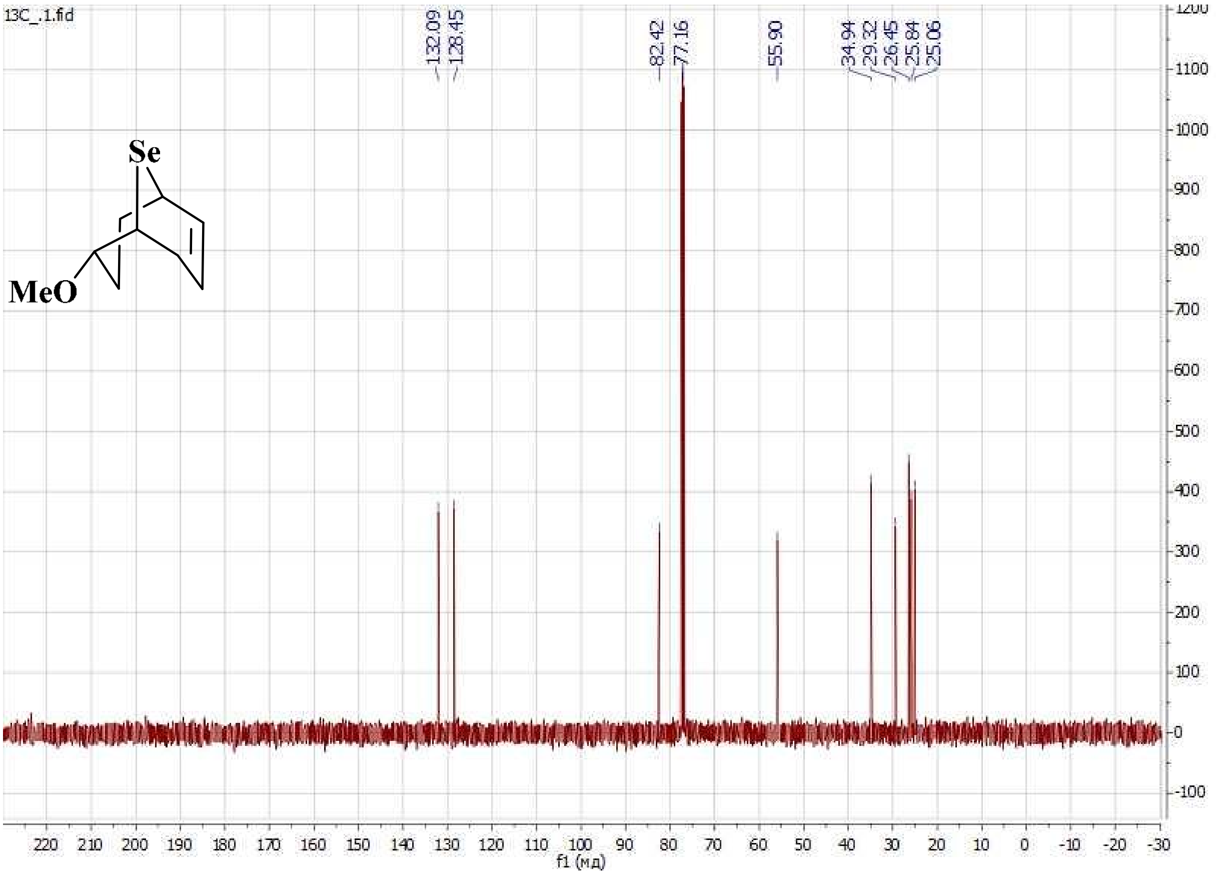

^{1}H- and ^{13}C-NMR spectra of compound 40 ($CDCl_3$)

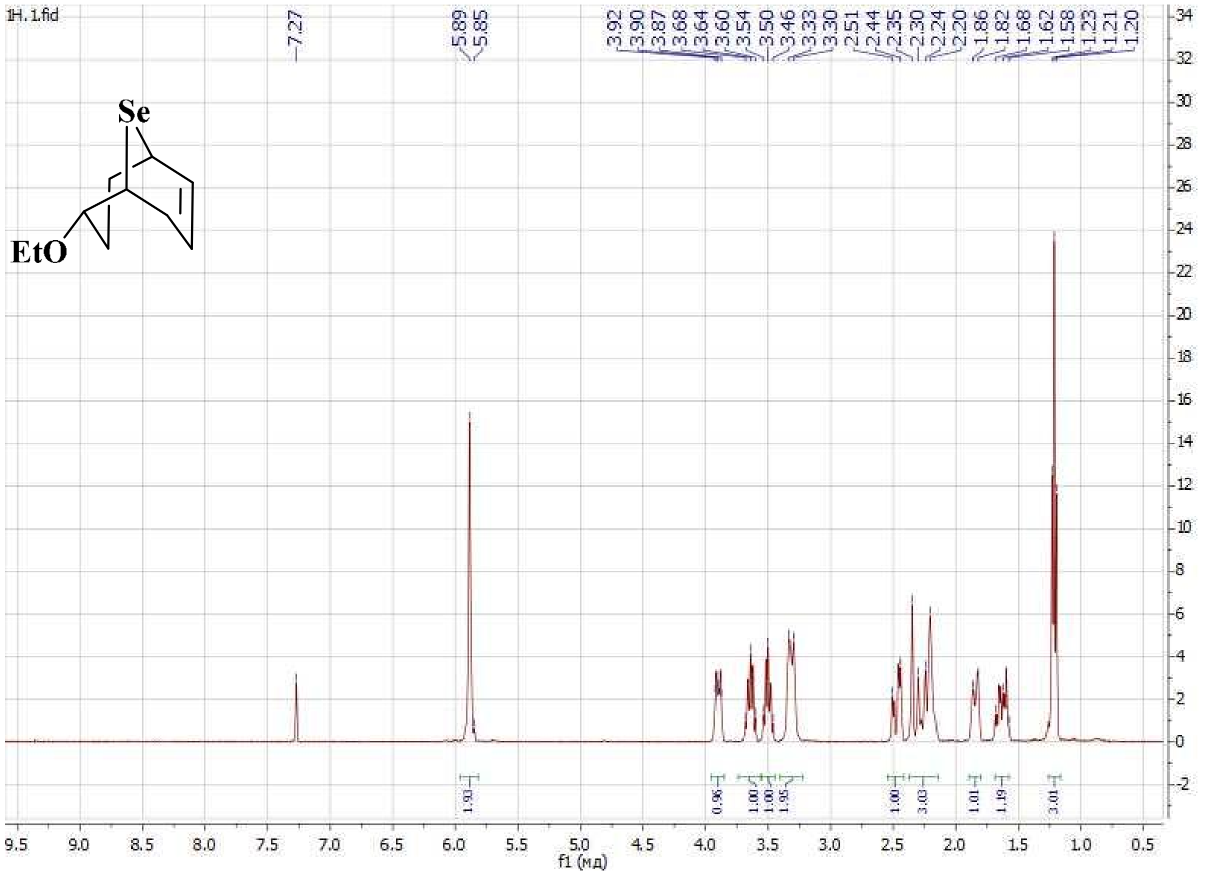

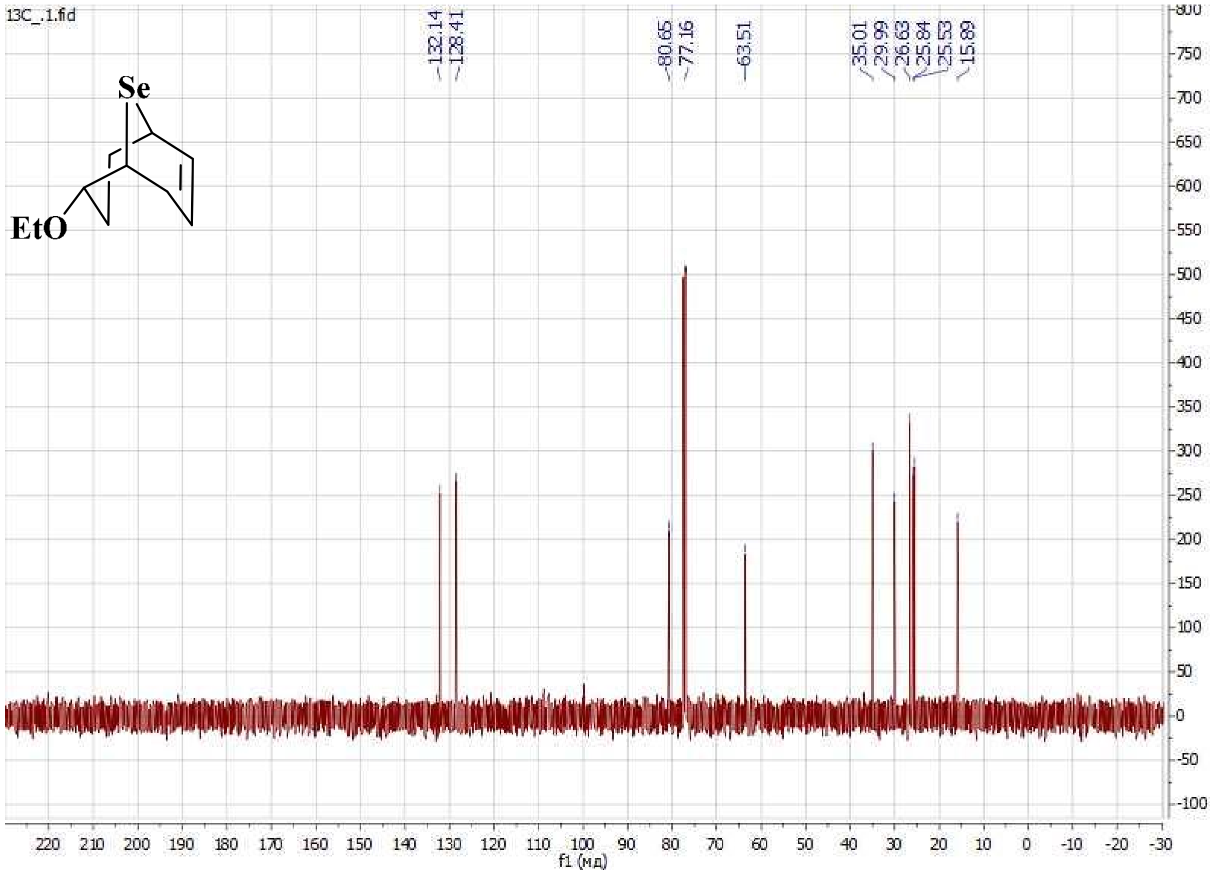

^{1}H- and ^{13}C-NMR spectra of compound 41 ($CDCl_3$)

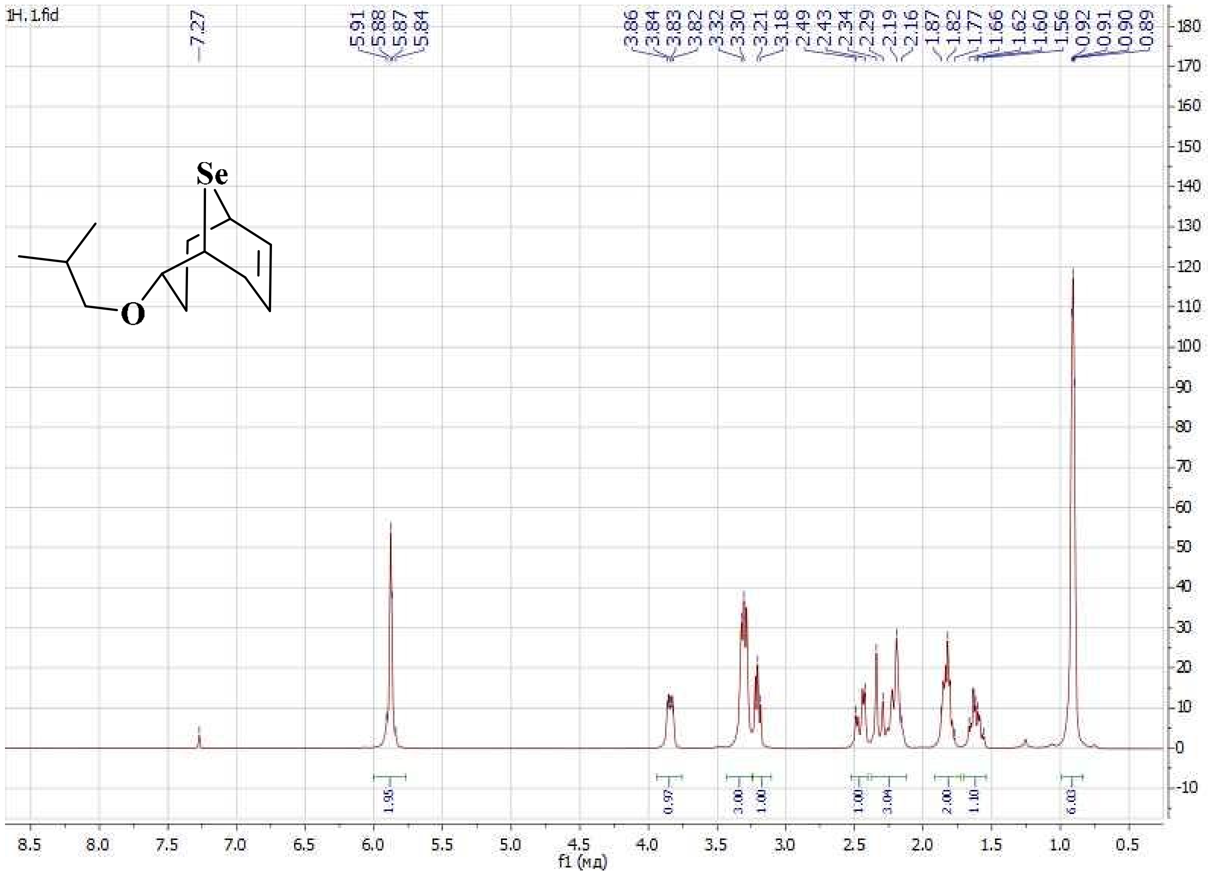

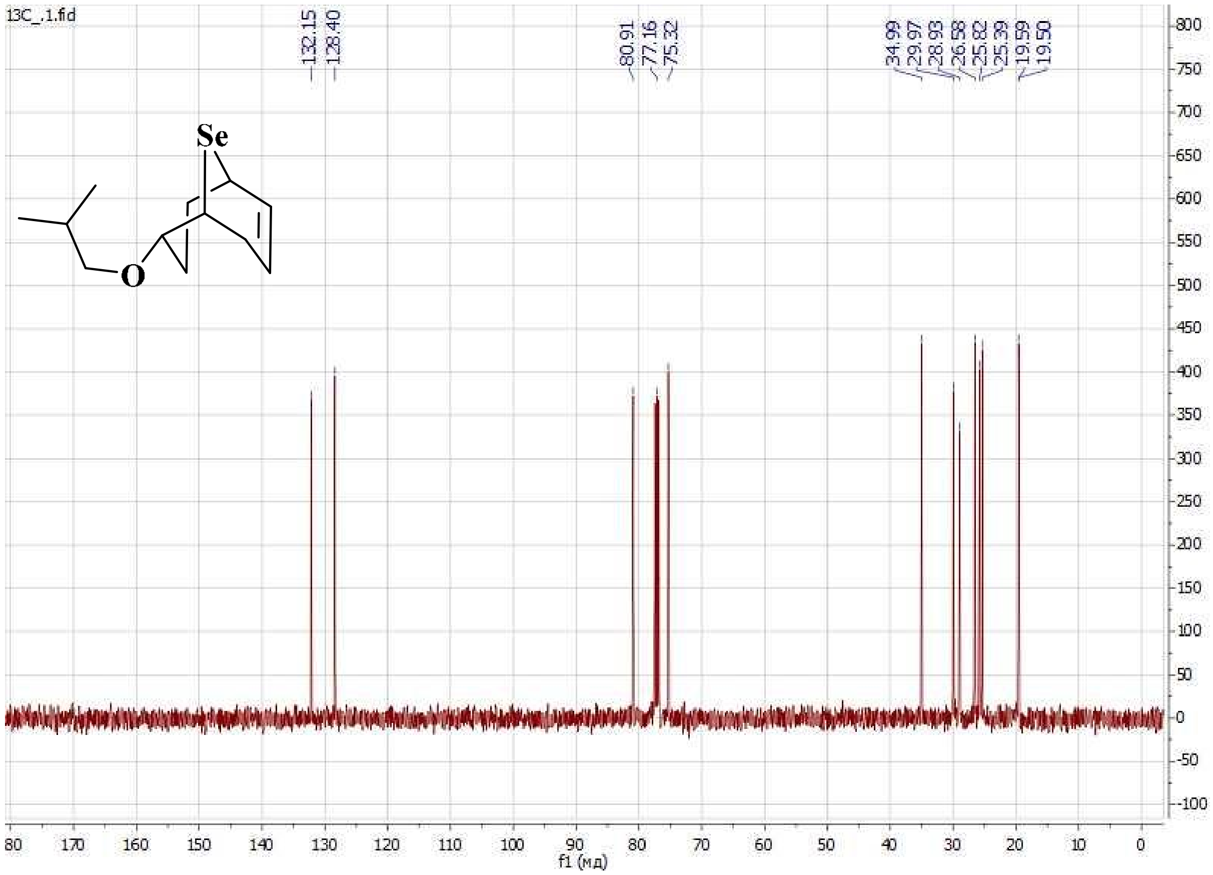

^{1}H- and ^{13}C-NMR spectra of compound 42 ($CDCl_3$)

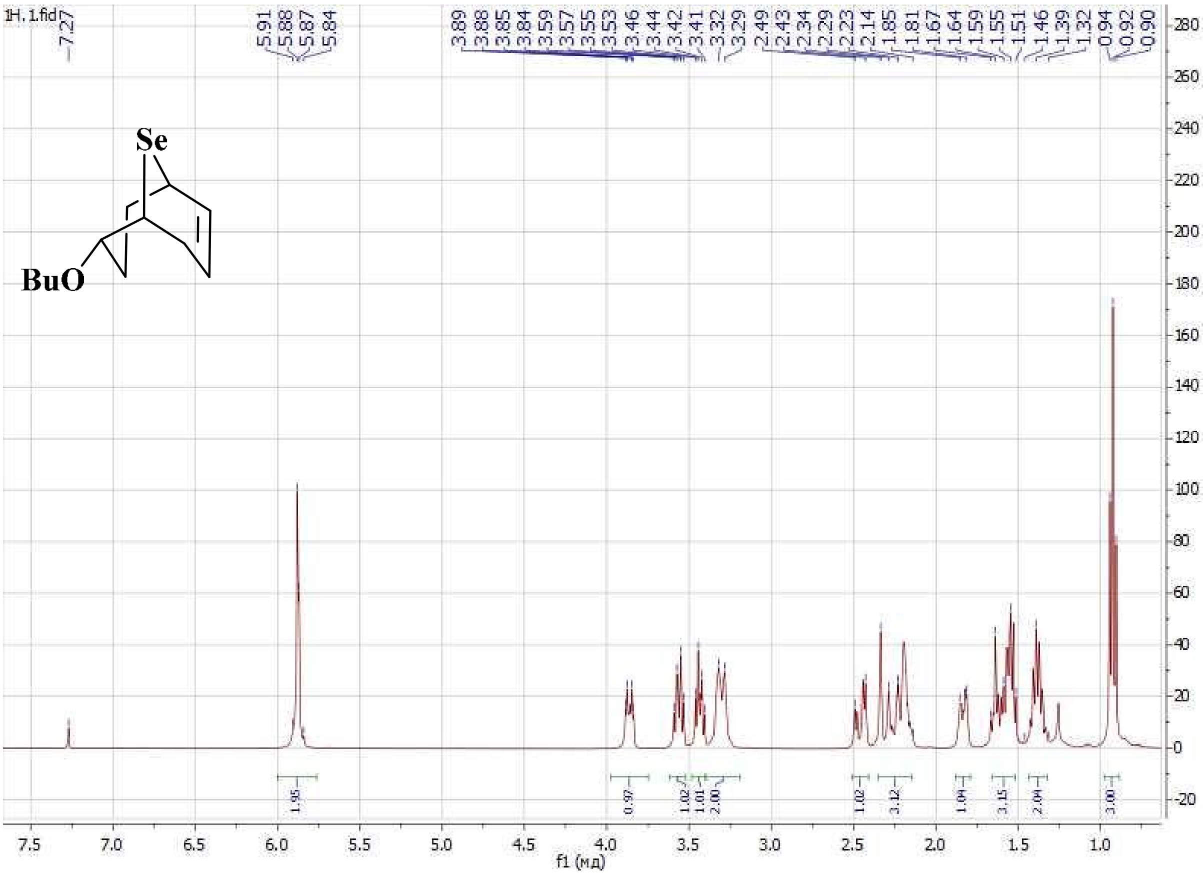

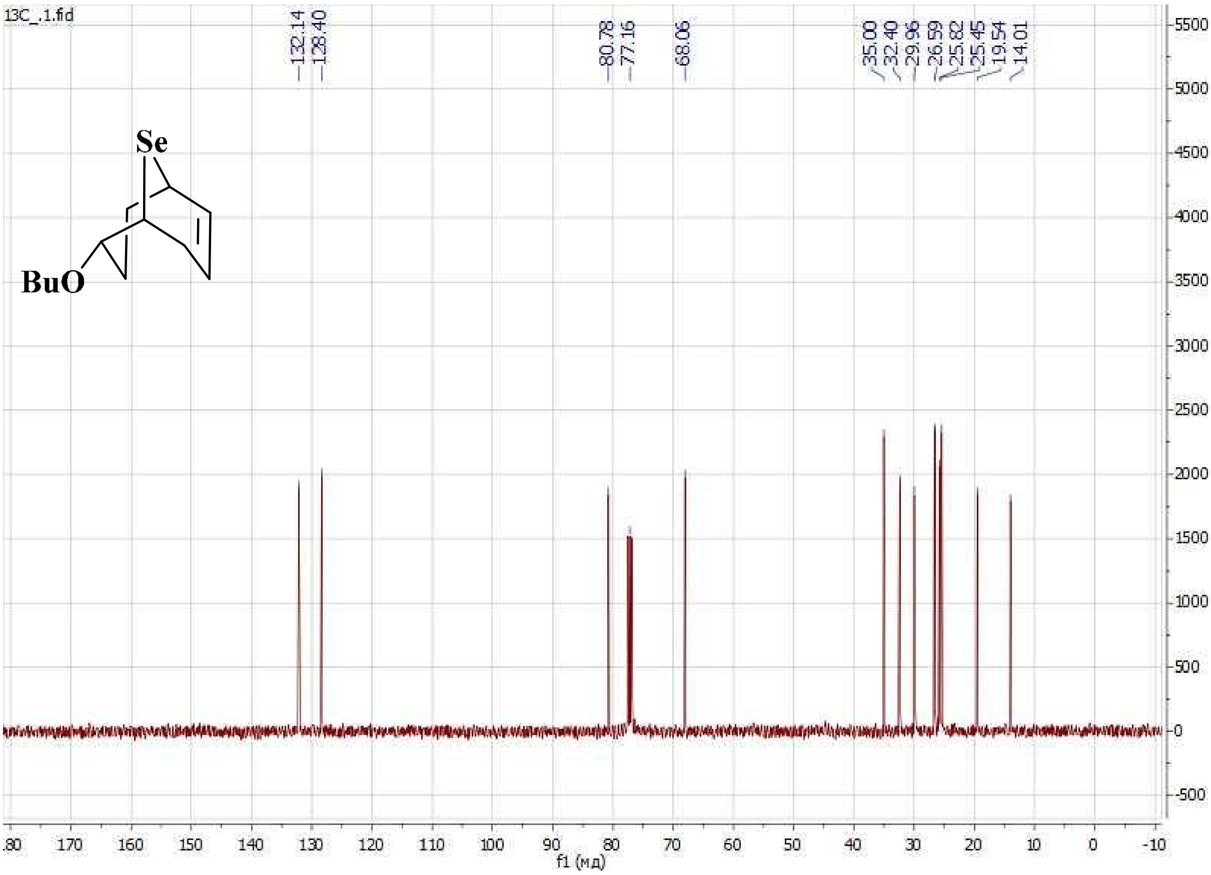

Supplement: Supplementary file 1 [file ijms-24-17485-s001.zip › ijms-2767427-supplementary.pdf]
